# Supplementary material for: N-Phenacyldibromobenzimidazoles—Synthesis Optimization and Evaluation of Their Cytotoxic Activity
Source: Molecules. 2022 Jul 7;27(14):4349. doi: 10.3390/molecules27144349 (PMC9315981; doi:10.3390/molecules27144349)

N-Phenacyldibromobenzimidazoles – synthesis optimization and evaluation of their cytotoxic activity

Anna Kowalkowska[1,*], Konrad Chojnacki[1], Maciej Multan[1], Jan K. Maurin[2], Edyta Łukowska-Chojnacka[1] and Patrycja Wińska[1]

[1] Warsaw University of Technology, Faculty of Chemistry, Noakowskiego St 3, 00-664 Warsaw, Poland, e-mail: anna.kowalkowska@pw.edu.pl, konrad.chojnacki@pw.edu.pl, edyta.chojnacka@pw.edu.pl, patrycja.winska.pw.edu.pl

[2] National Medicines Institute, Chełmska St. 30/34, 00-725 Warsaw, Poland

National Centre for Nuclear Research, Andrzeja Sołtana St. 7, 05-400 Otwock, Poland, e-mail: j.maurin@nil.gov.pl

1. **HPLC analysis - general remarks** **2**
2. **HPLC chromatograms of optimization reactions (Table 1, Entry 1-35)** **3**

1. HPLC analysis - general remarks

HPLC analyses were performed on PE NELSON NCI900 chromatograph (Waltham, MA, USA) equipped with a UV-VIS (Perkin Elmer 785A detector, Waltham, MA, USA) and a reverse-phase column Hichrom HI-5C18-3959 (250 x 1.0 mm, 5 µm). 10 µl of sample was injected. A gradient method comprising water/ acetonitrile was applied as follows: 0-20 min 20/80 (1 ml/min), 20-25 min 20/80 – 0/100 (1 ml/min) 25-60 min 0/100 (1 ml/min). UV–VIS detector was set at a wavelength of 254 nm.

The calibration curve was prepared using seven concentrations of the model compound, 2-(5,6-dibromo-1*H*-benzimidazol-1-yl)-1-(2,4-dichlorophenyl)ethenone **4e** (1.520, 1.013, 0.676, 0.450, 0.300, 0.200, 0.133 mg/ml).

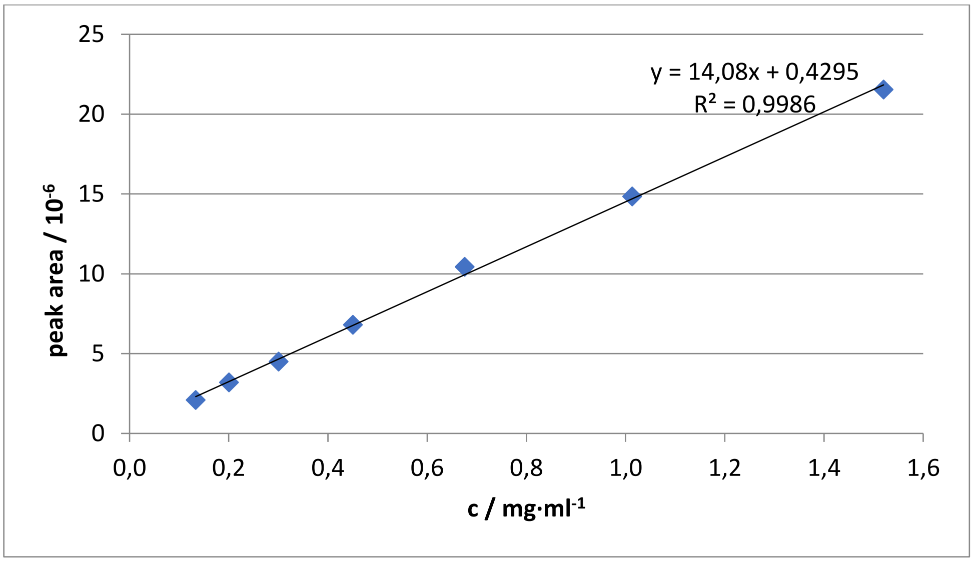

Figure S1. The calibration curve.

Procedure for optimization reactions (see Table 1 in the manuscript).

To the 5,6-dibromobenzimidazole **1** (0.1 mmol, 27.6 mg) in a solvent (5 ml, see Table 1) 2,4-dichlorophenacyl chloride **3e** (see Table 1) was added, followed by a base (see Table 1). The mixture was magnetically stirred (temperature and time given in Table 1). The solvent was evaporated, H_2O (10 ml) and CH_2Cl_2 (10 ml) were added, the phases were separated, the water phase was extracted with CH_2Cl_2 (2 x 10 ml), the combined organic phases were washed with H_2O (2 x 10 ml), dried with $MgSO_4$ and evaporated to dryness. The residue was dissolved in the volumetric flask (5 ml) in THF and diluted with MeCN (50 µl of THF solution + 250 µl of MeCN). The concentration of the product **4e** was determined by HPLC, injecting 10 µl of a sample.

1. HPLC chromatograms of optimizations reactions presented in Table 1.

Table 1, Entry 1

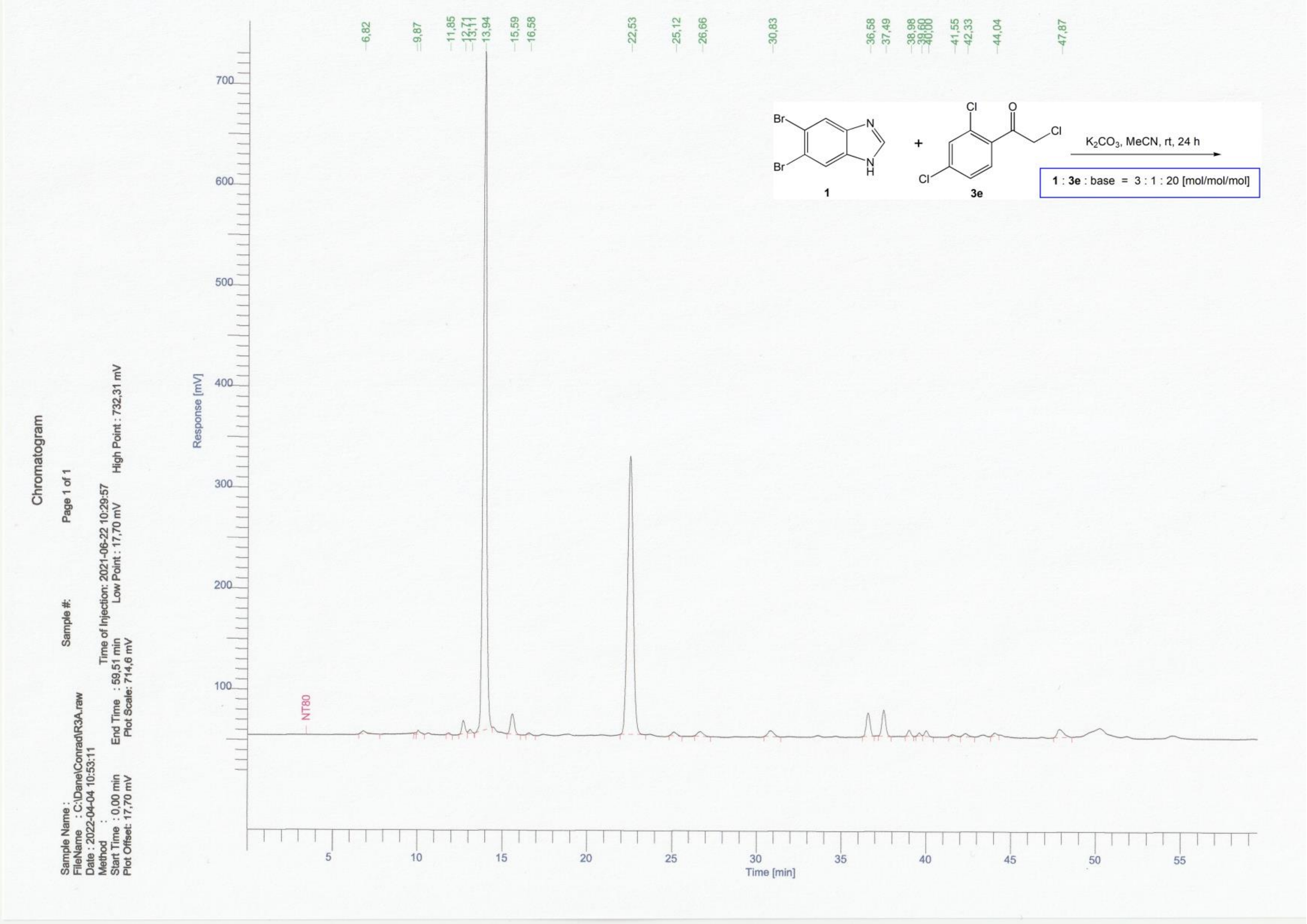

Table 1, Entry 2

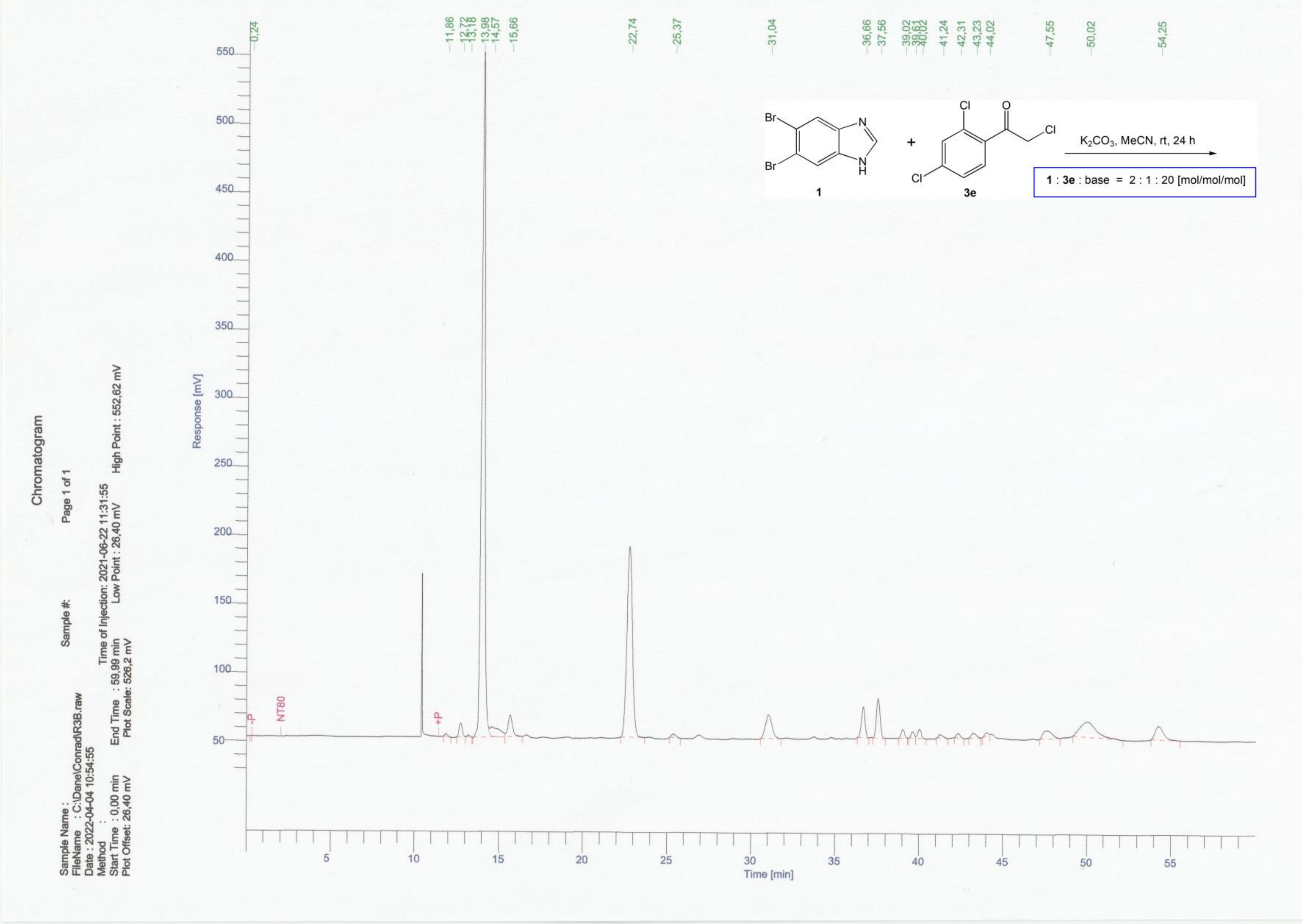

Table 1, Entry 3

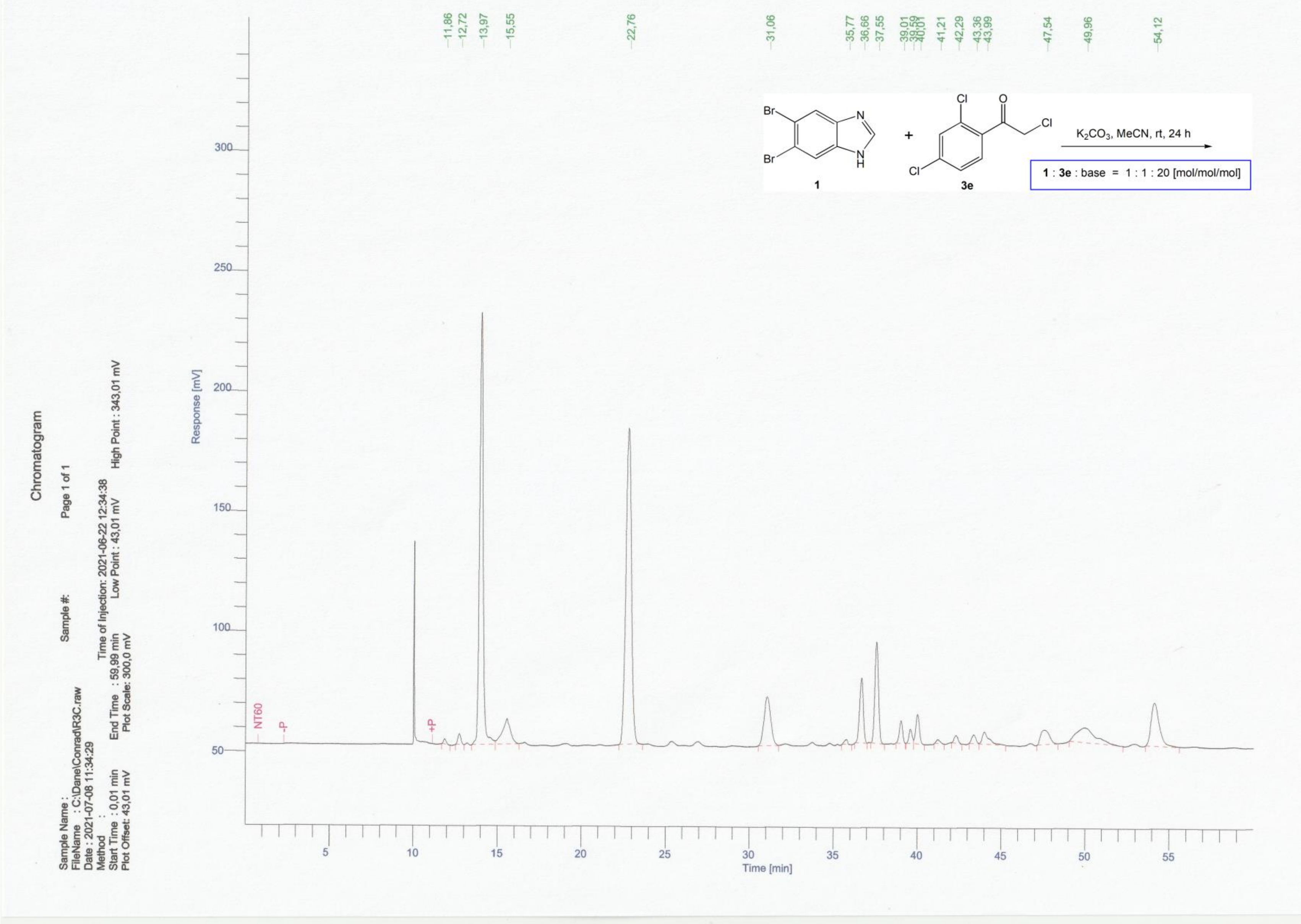

Table 1, Entry 4

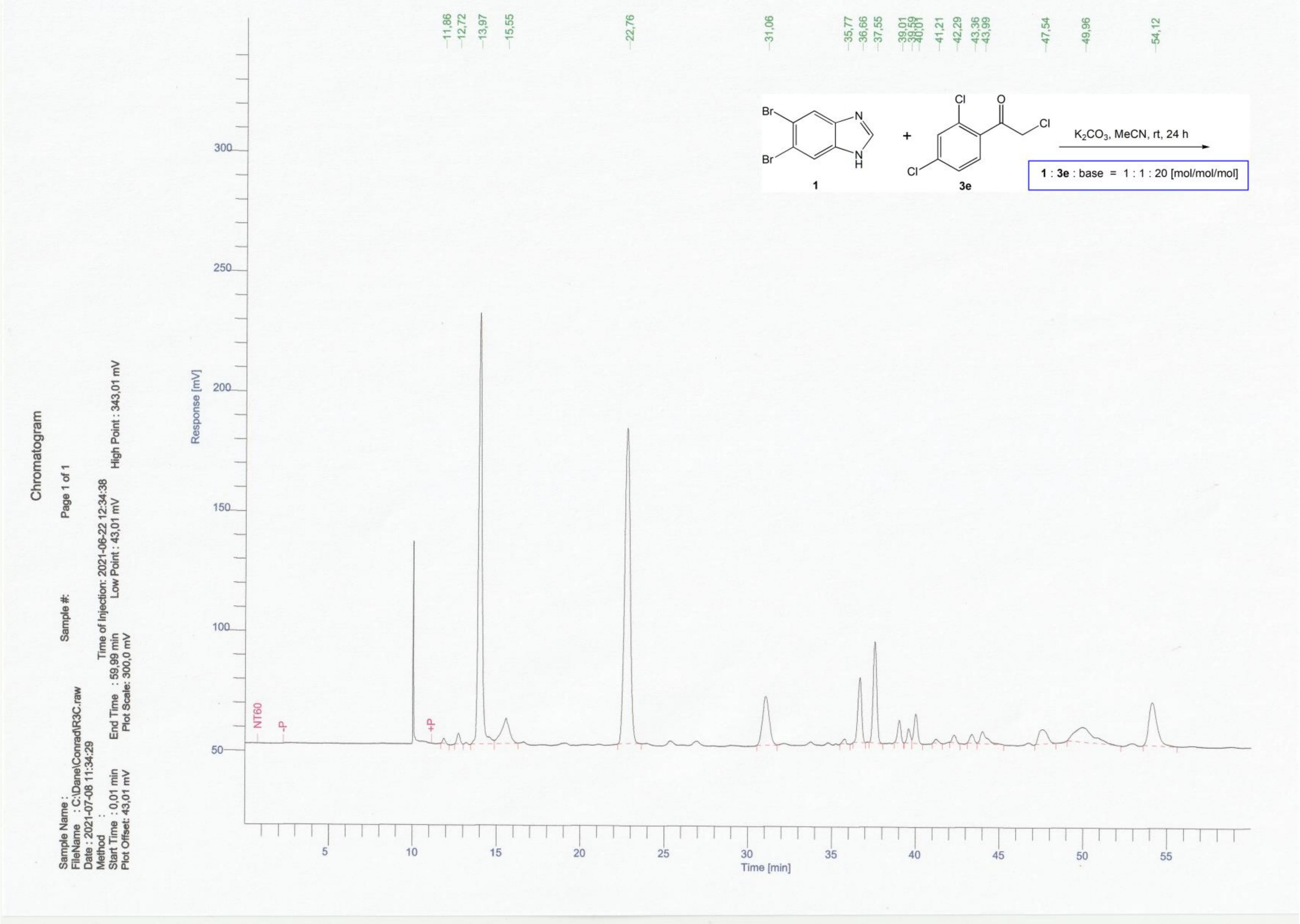

Table 1, Entry 5

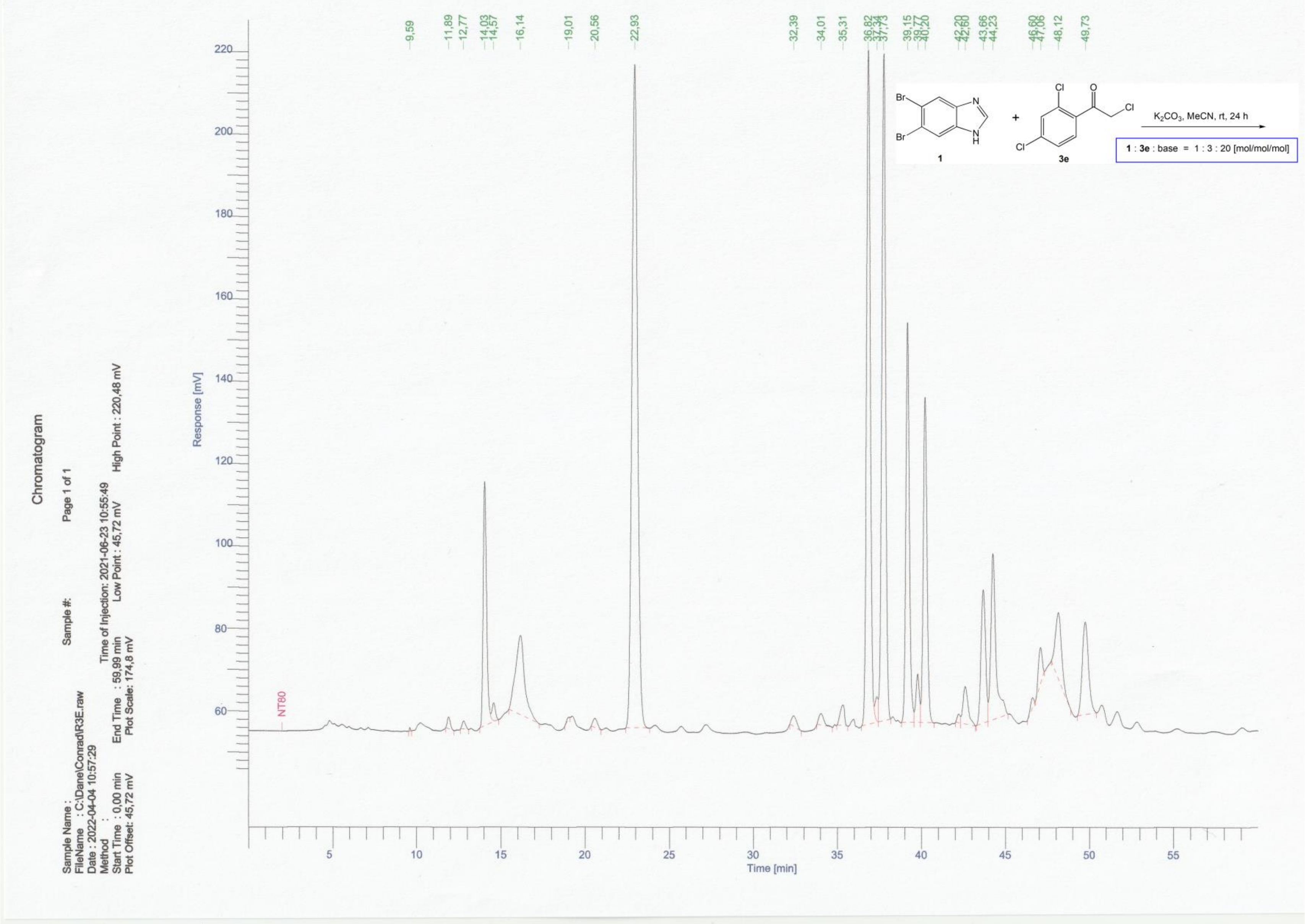

Table 1, Entry 6

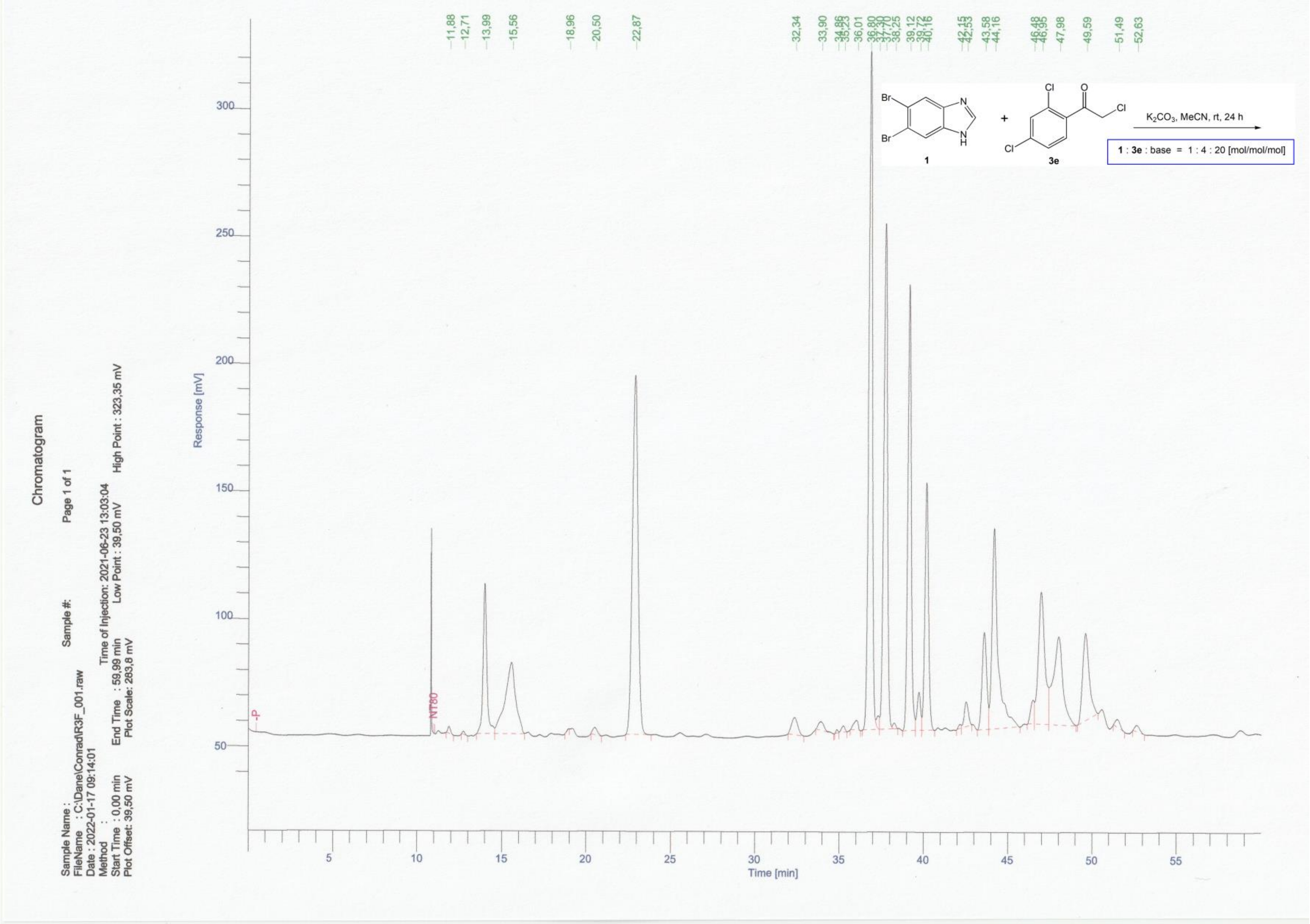

Table 1, Entry 7

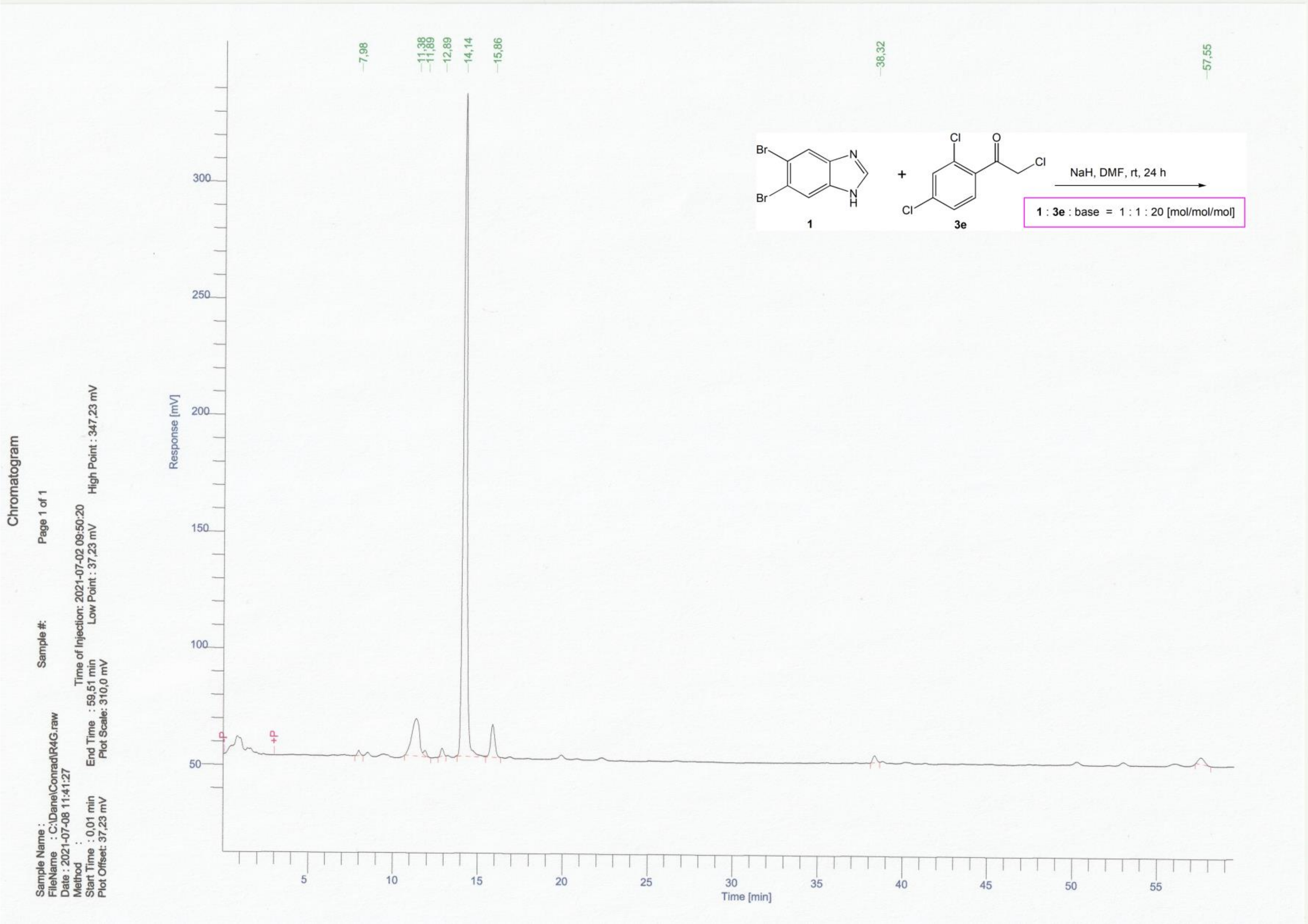

Table 1, Entry 8

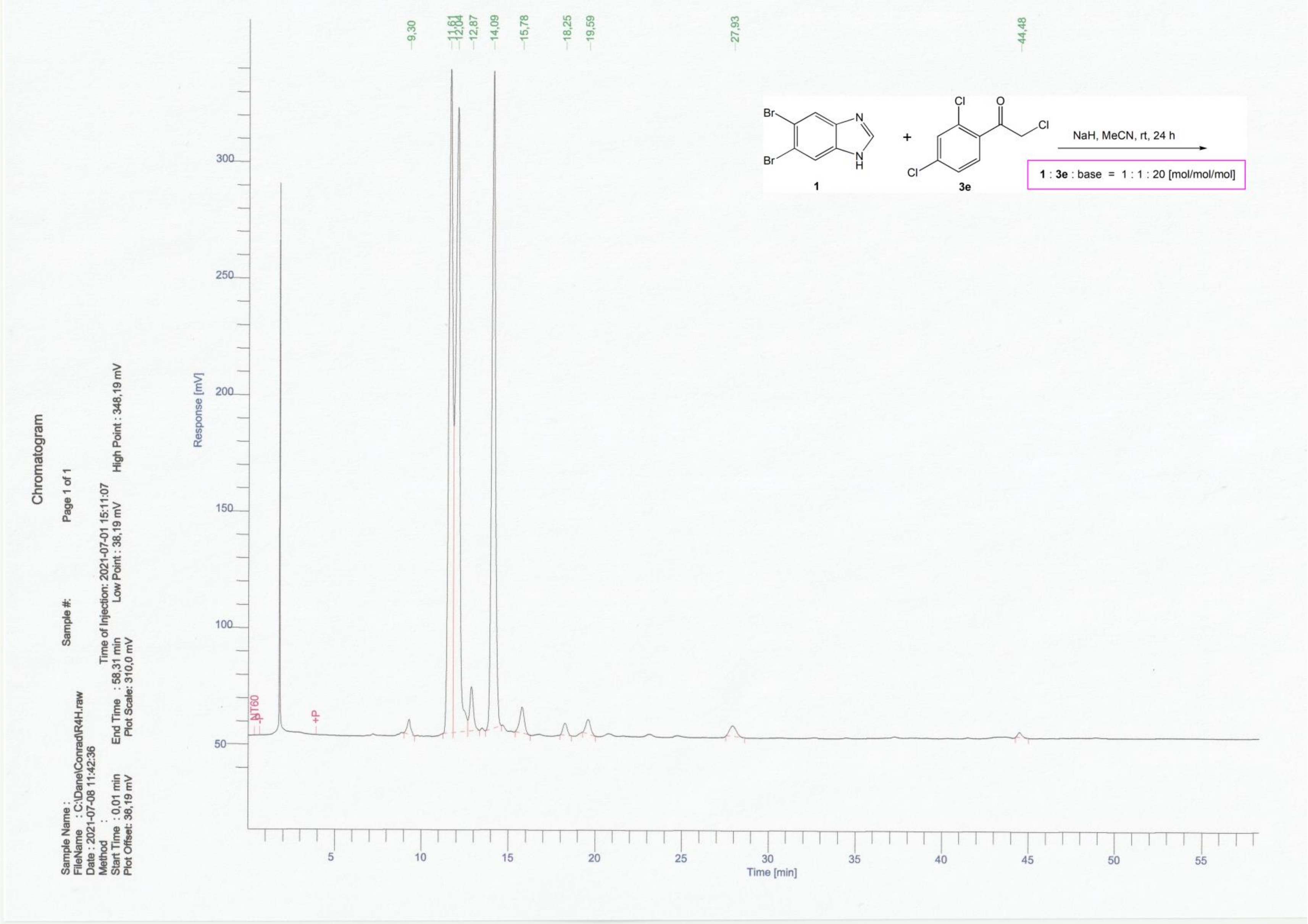

Table 1, Entry 9

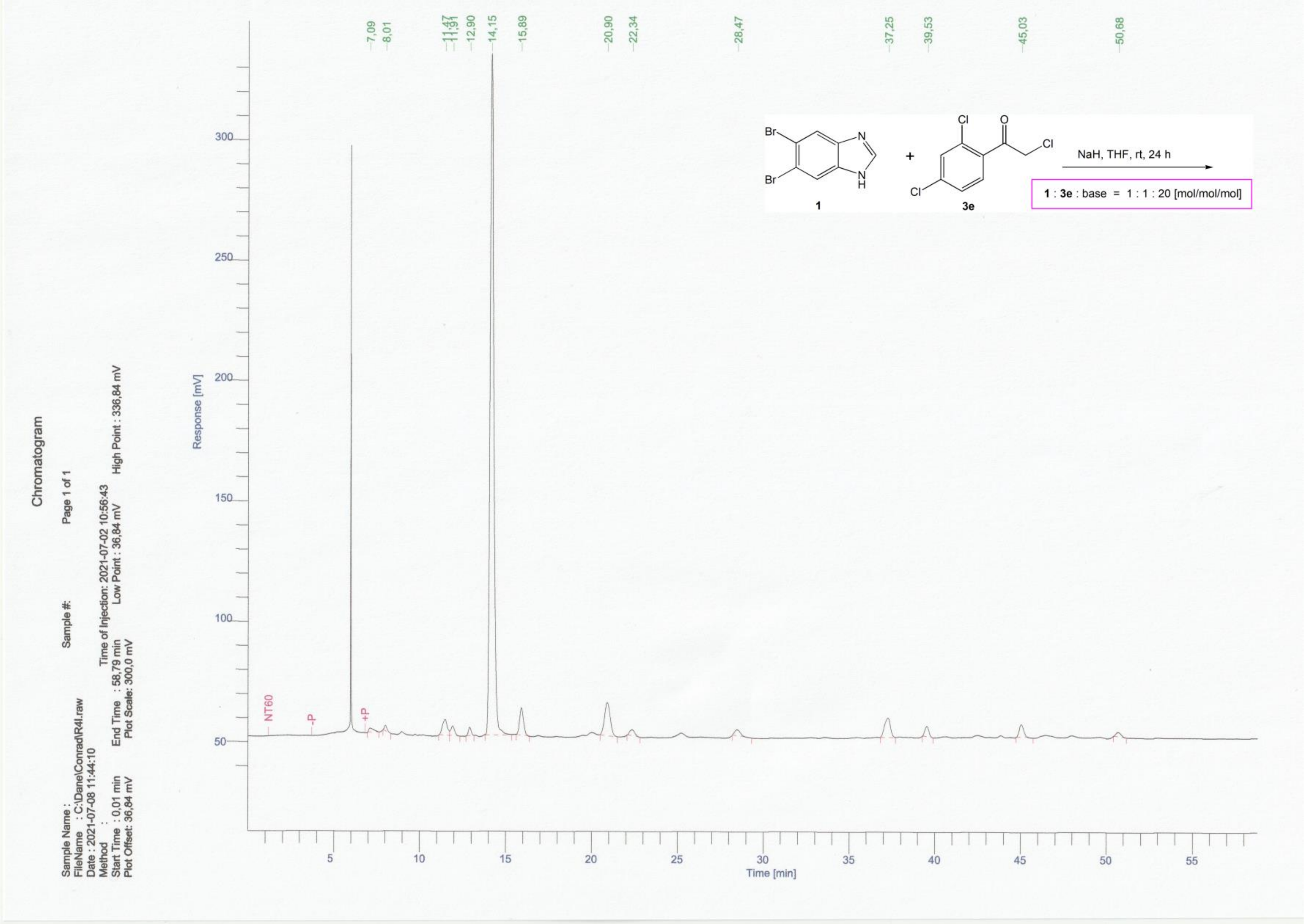

Table 1, Entry 10

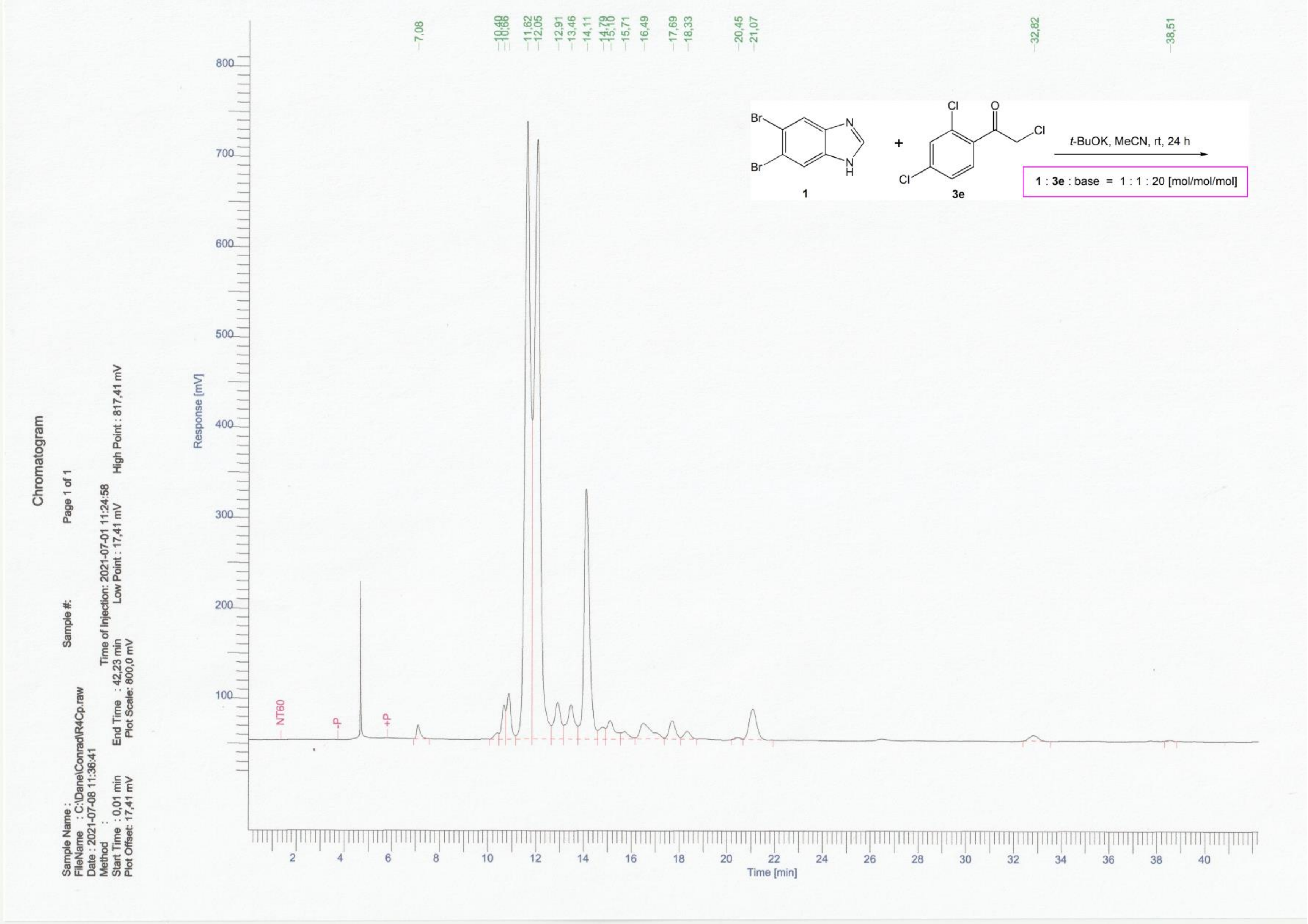

Table 1, Entry 11

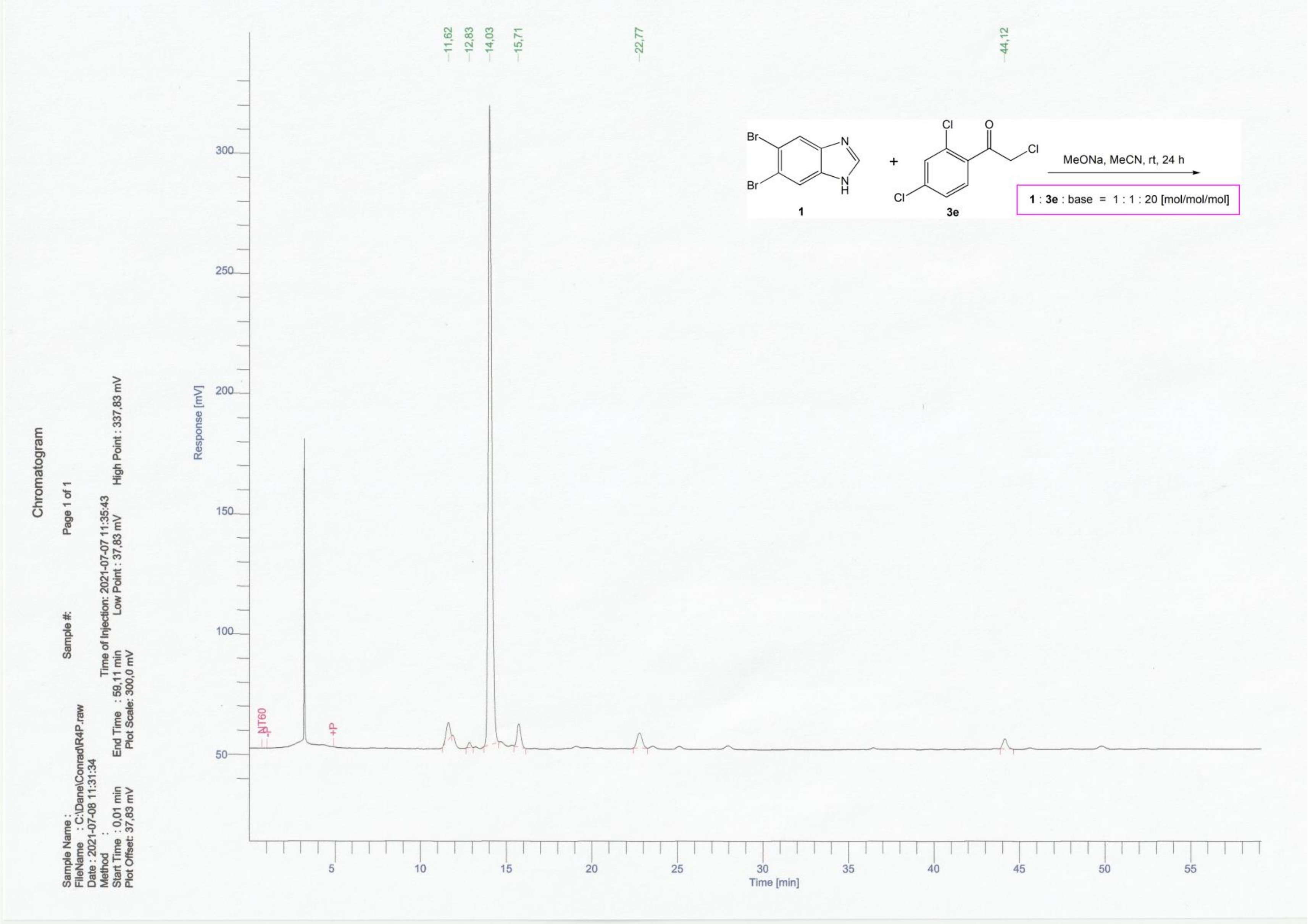

Table 1, Entry 12

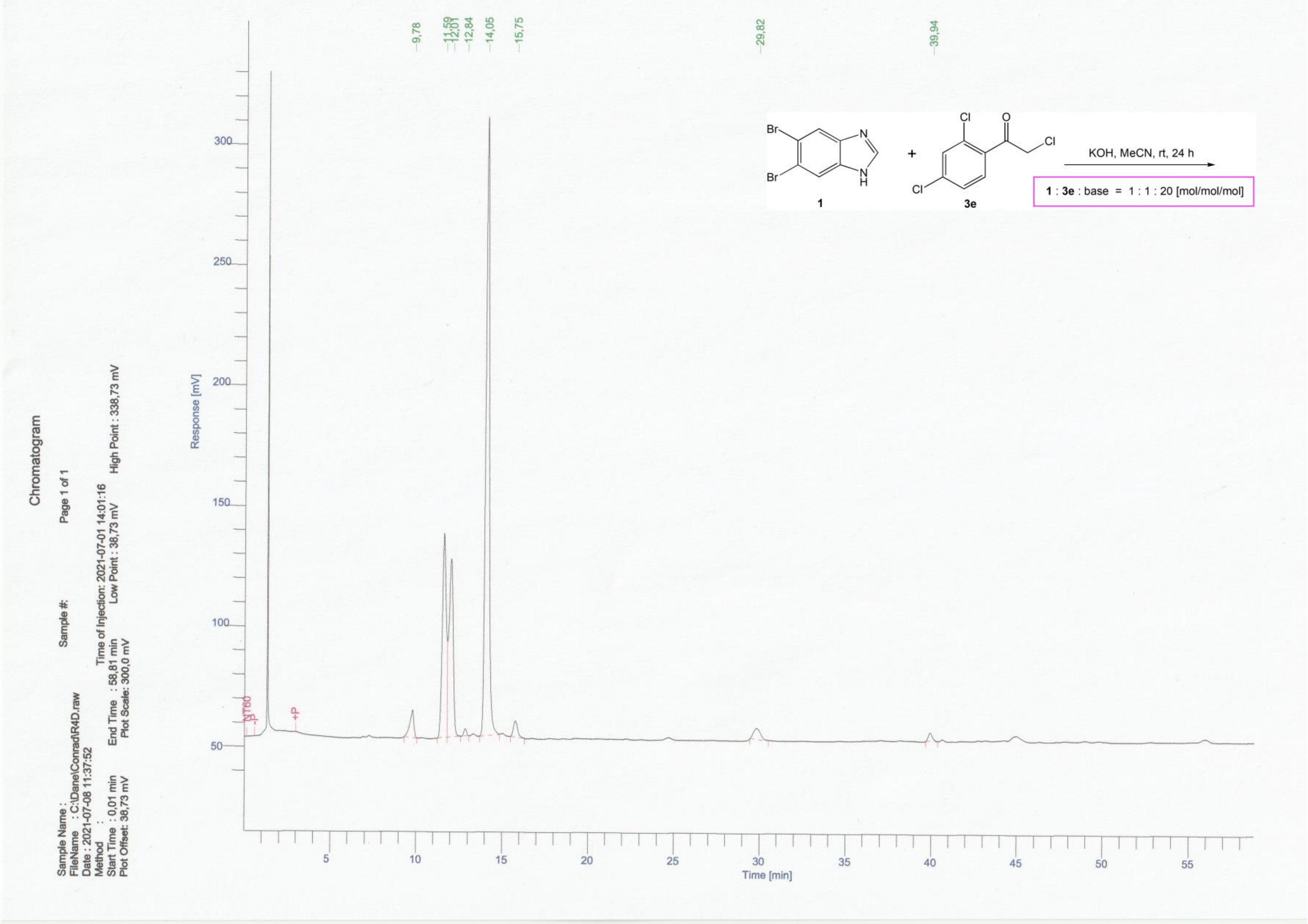

Table 1, Entry 13

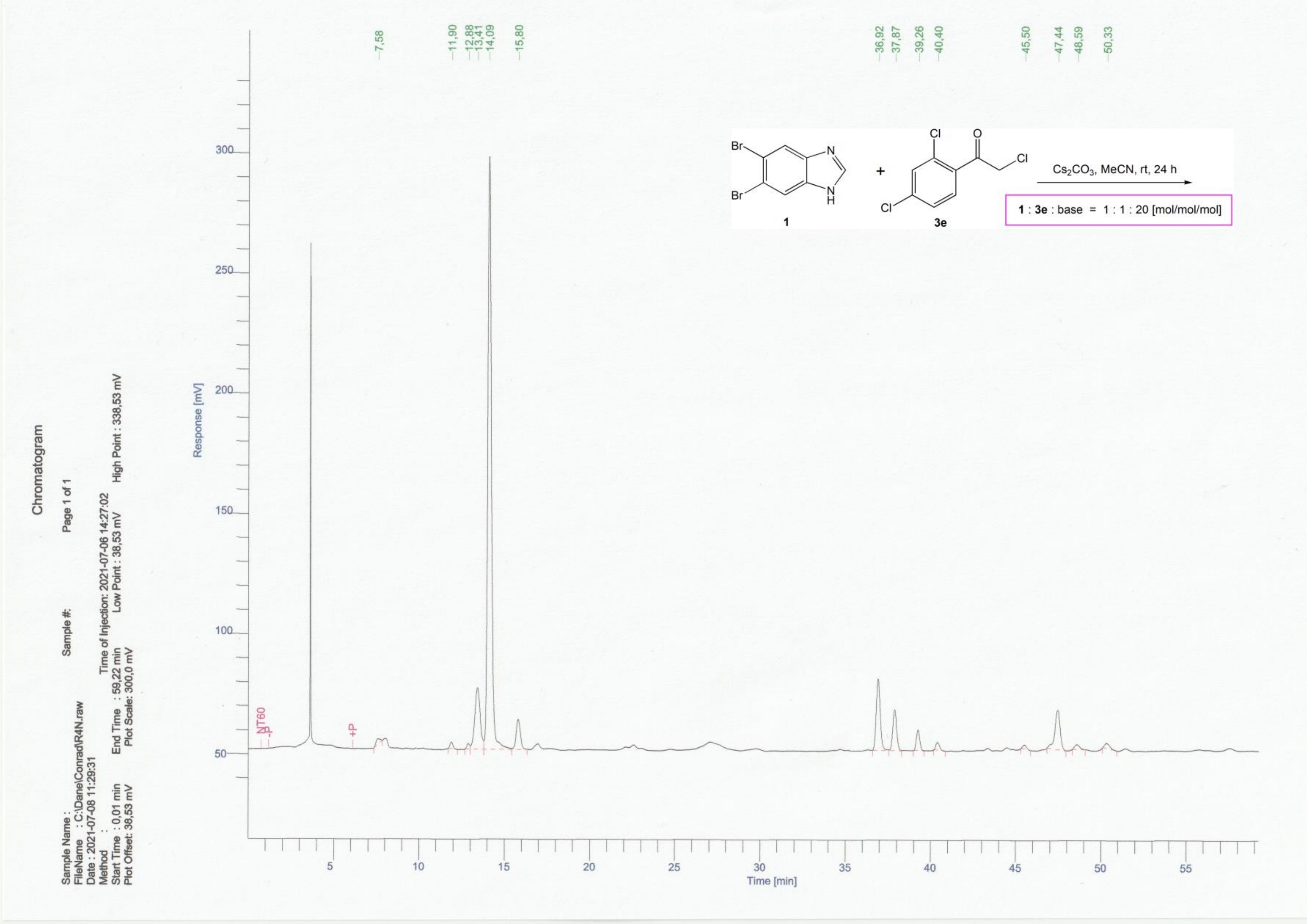

Table 1, Entry 14

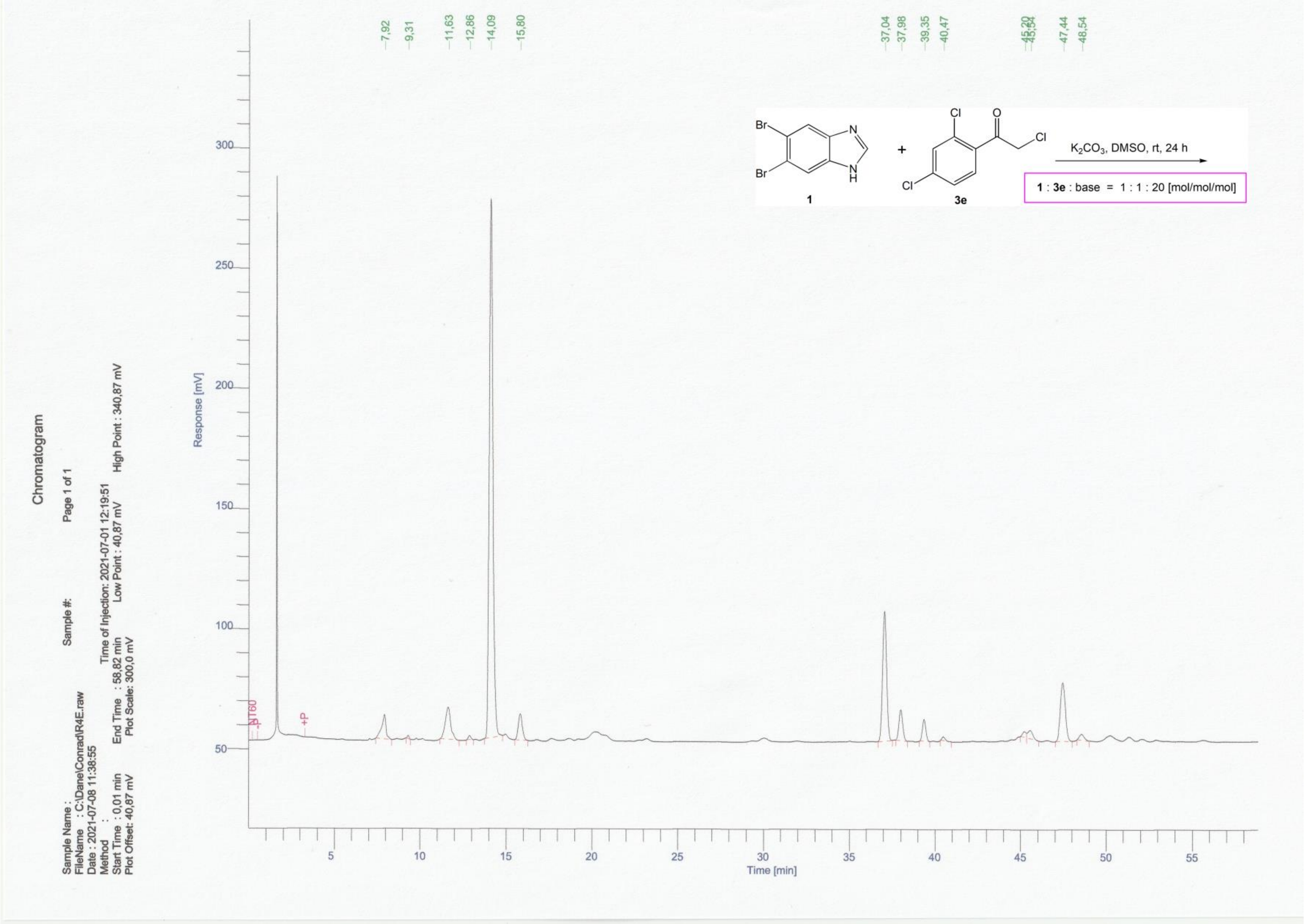

Table 1, Entry 15

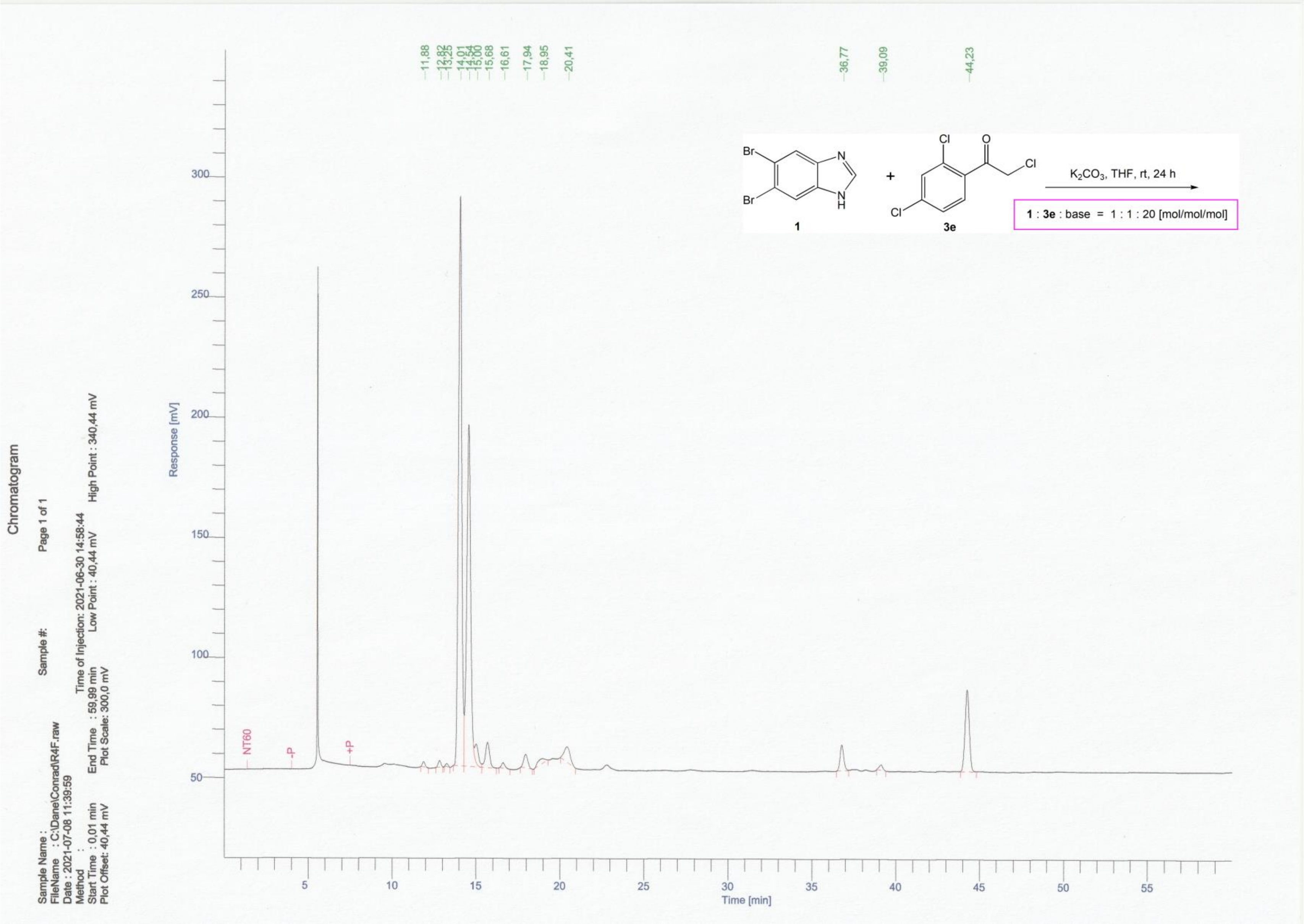

Table 1, Entry 16

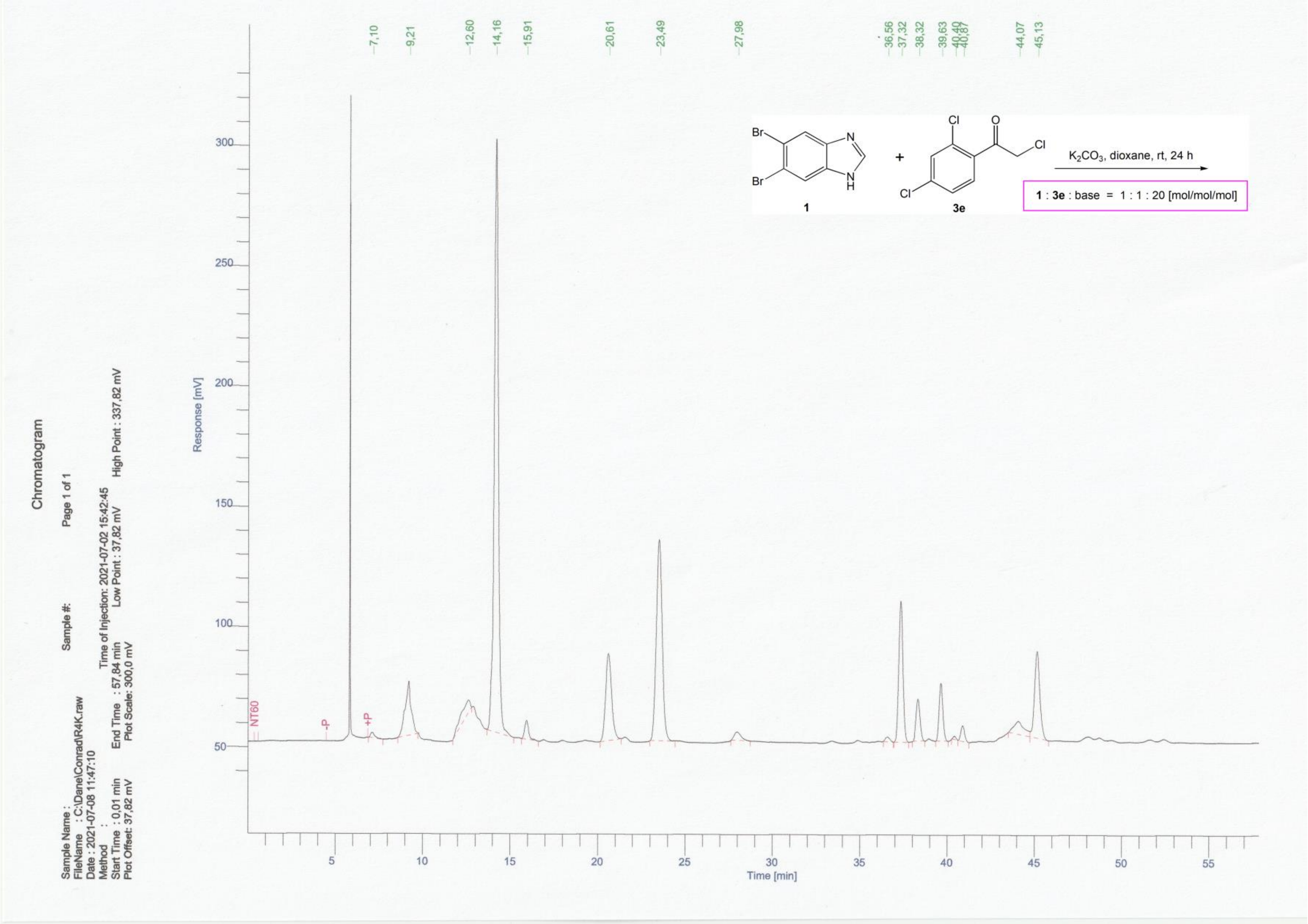

Table 1, Entry 17

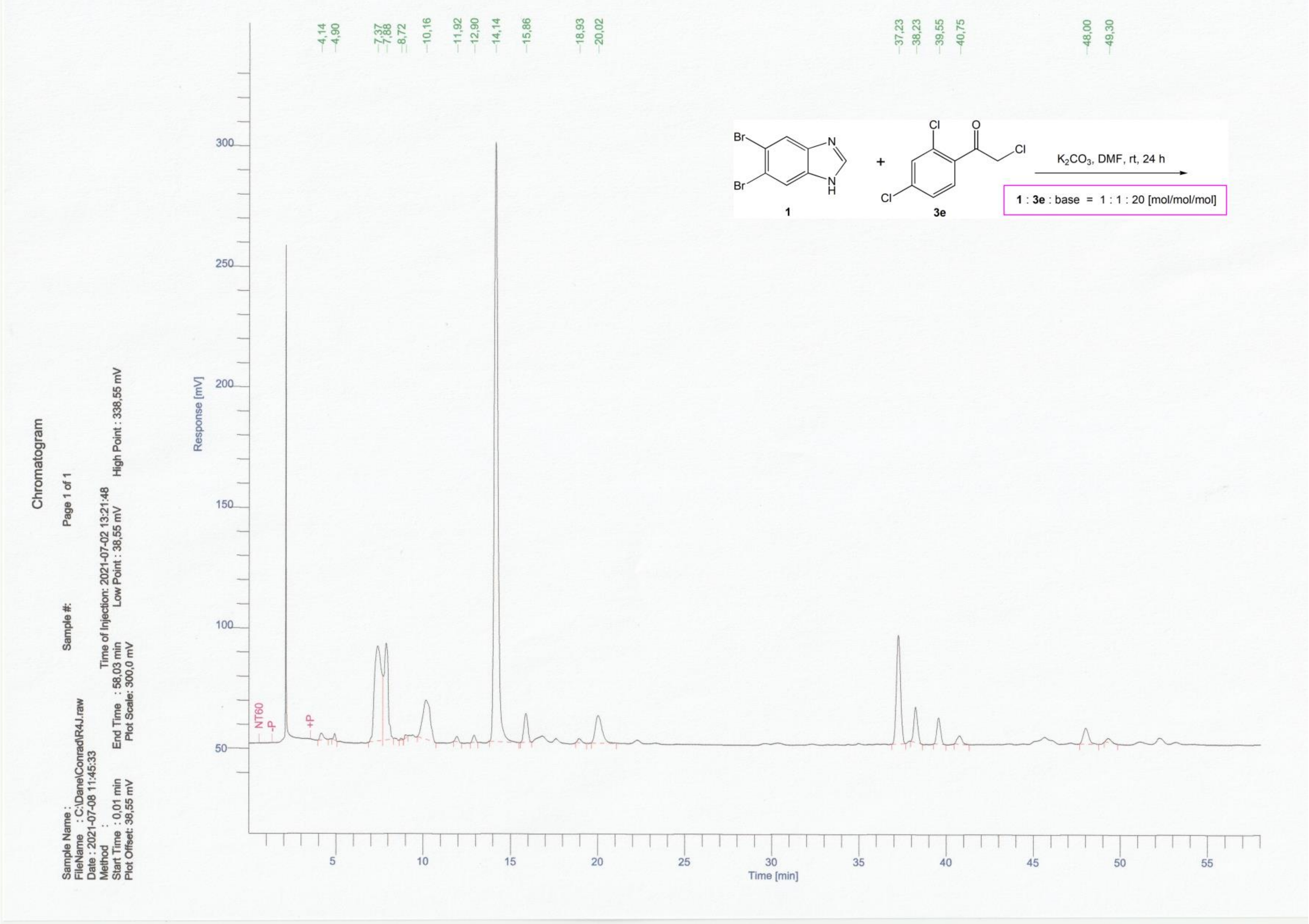

Table 1, Entry 18

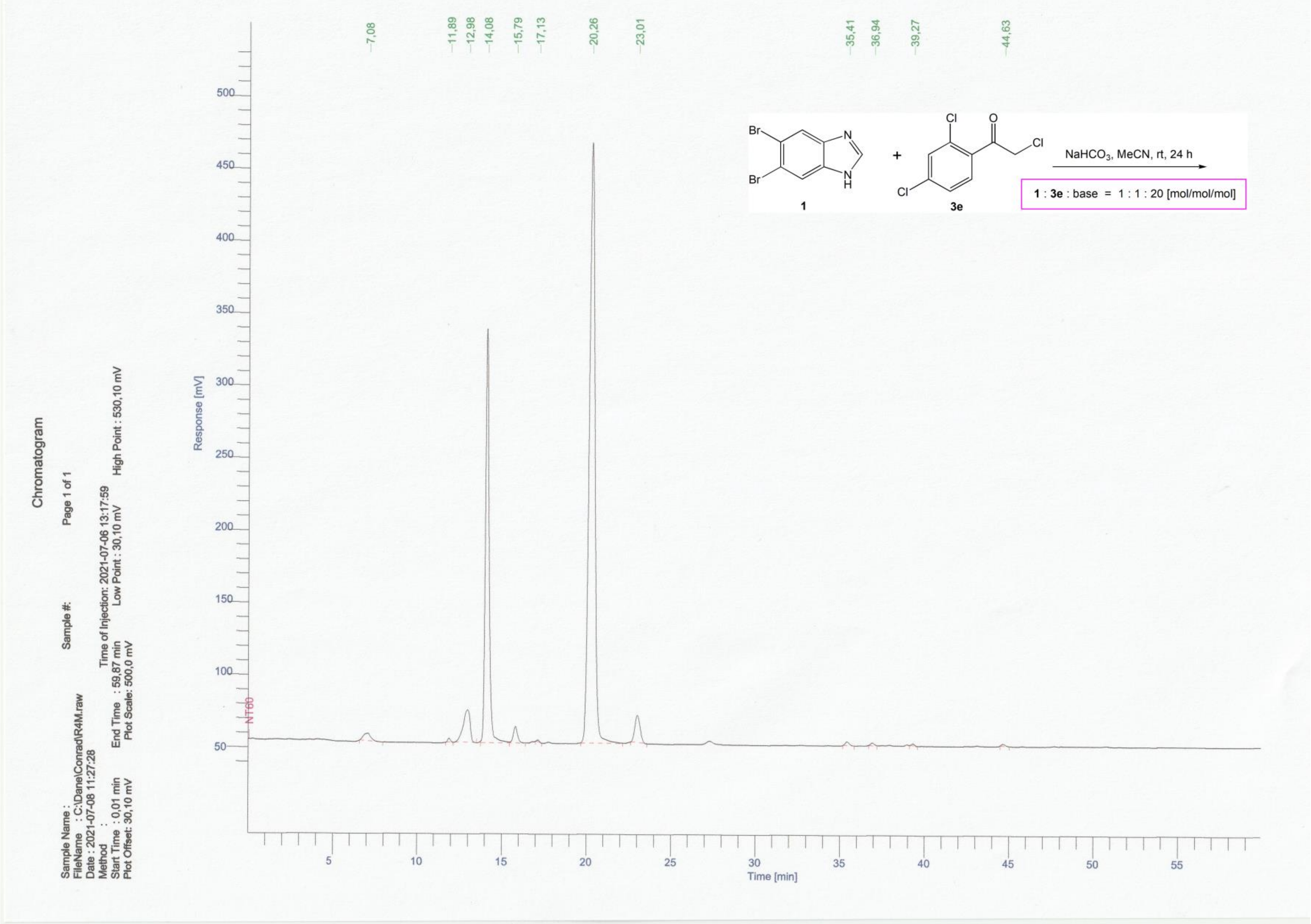

Table 1, Entry 19

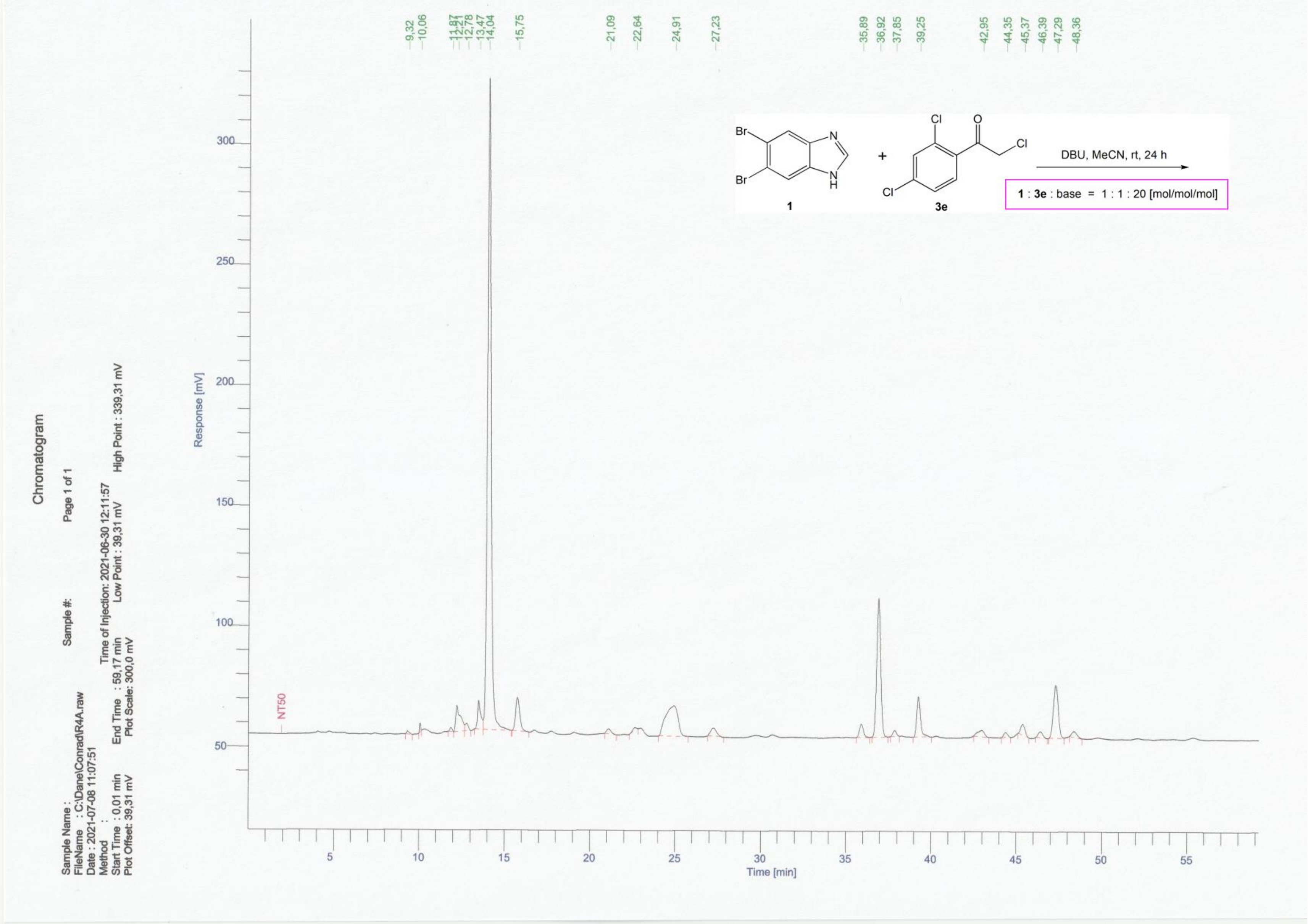

Table 1, Entry 20

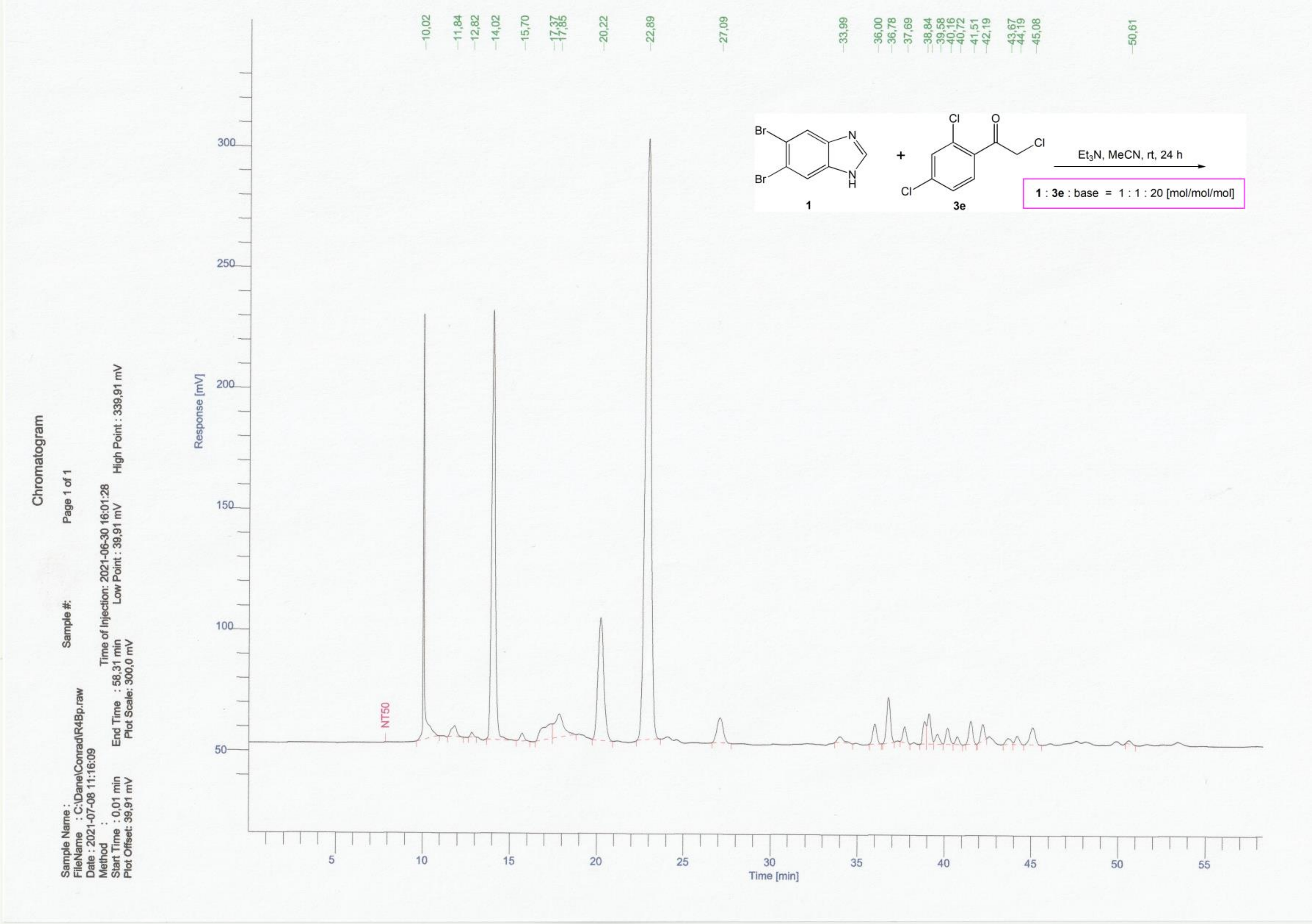

Table 1, Entry 21

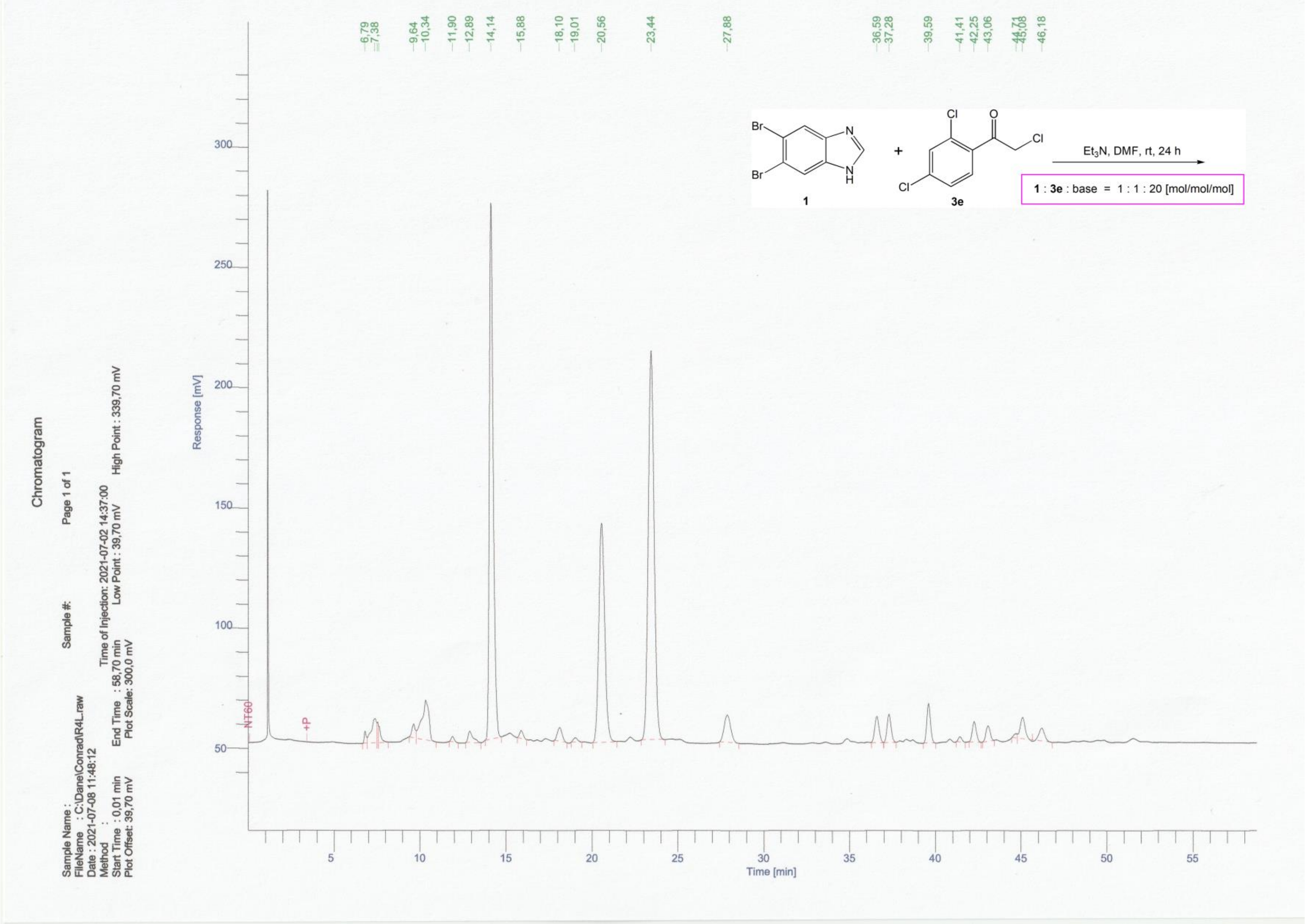

Table 1, Entry 22

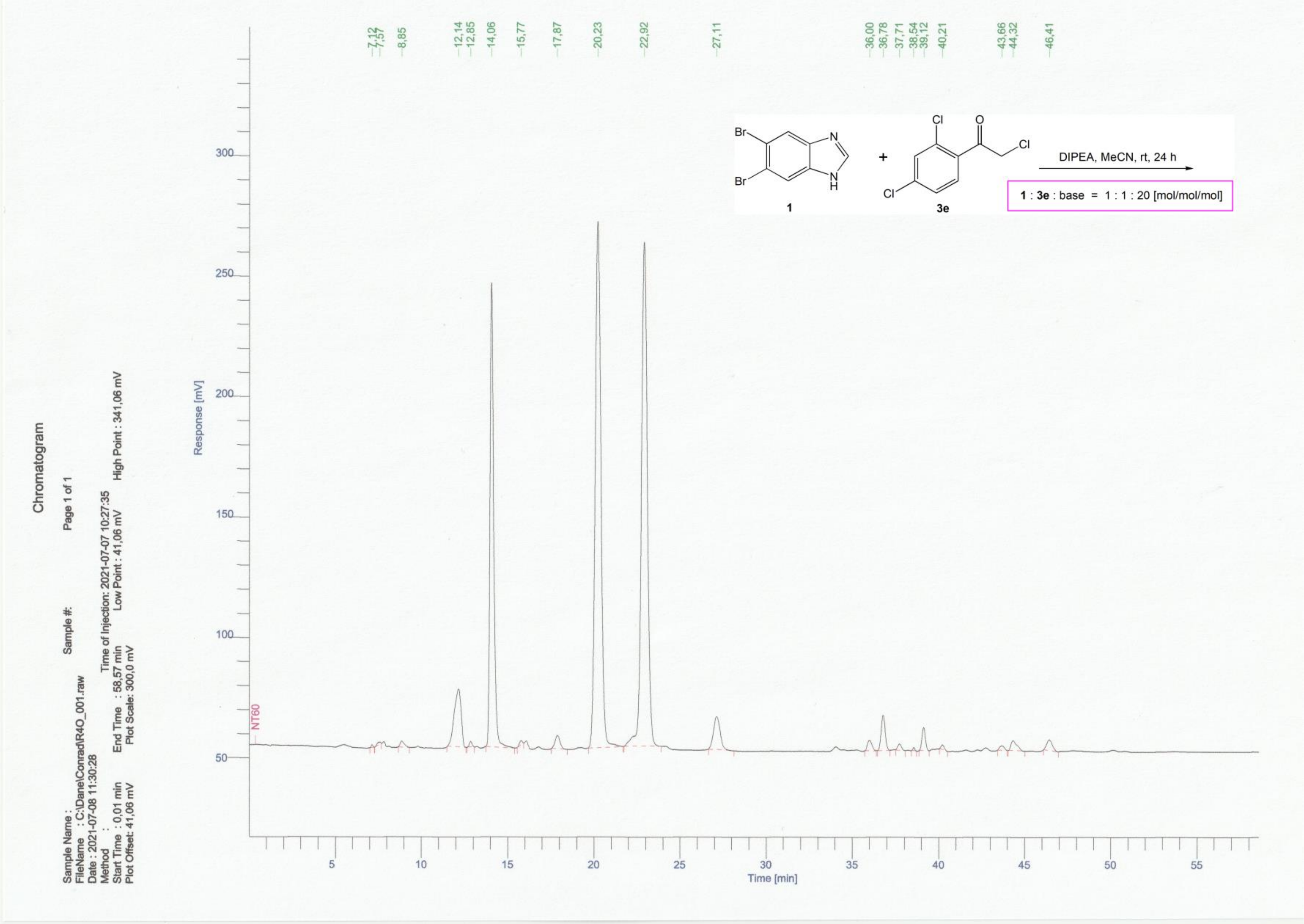

Table 1, Entry 23

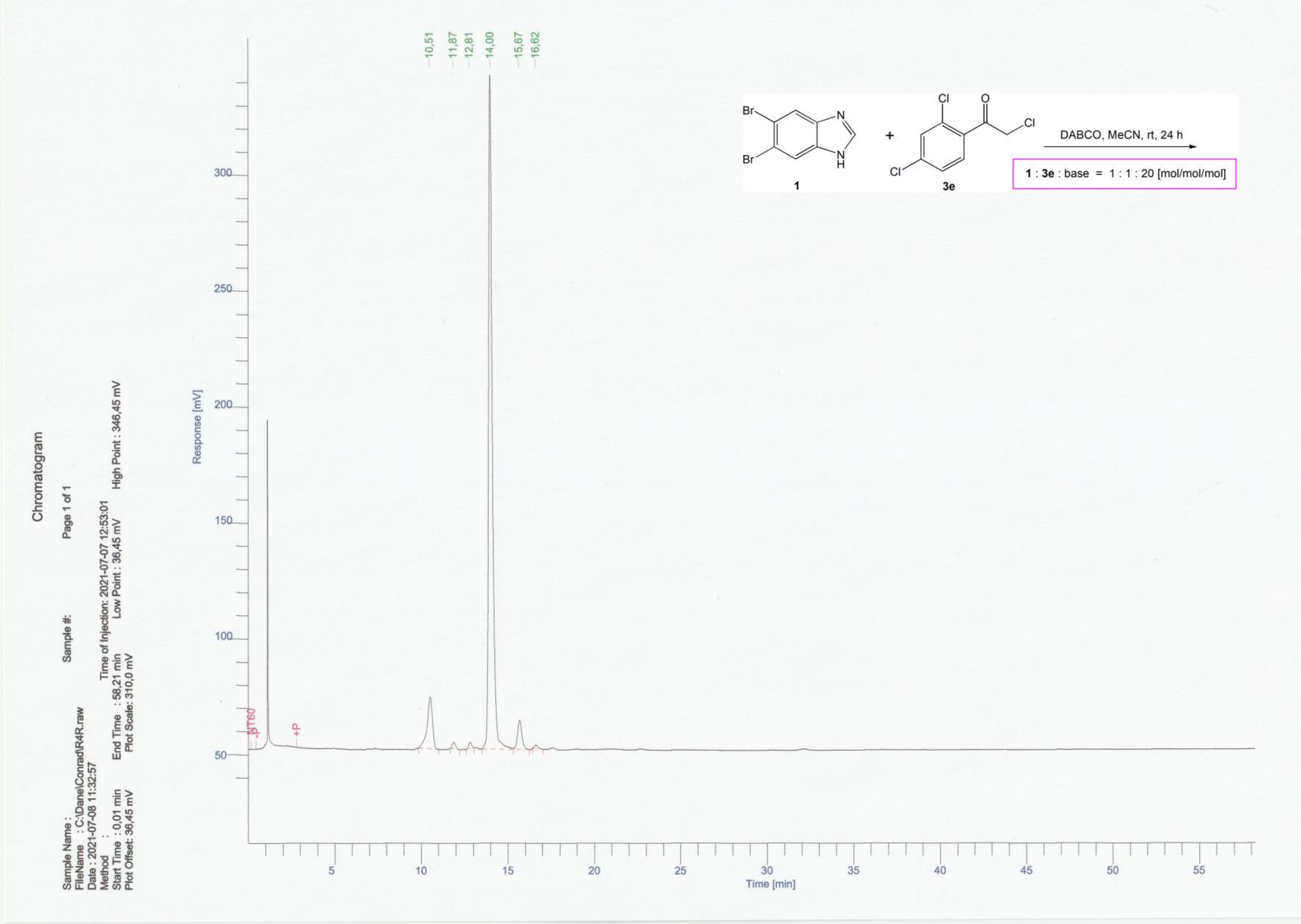

Table 1, Entry 24

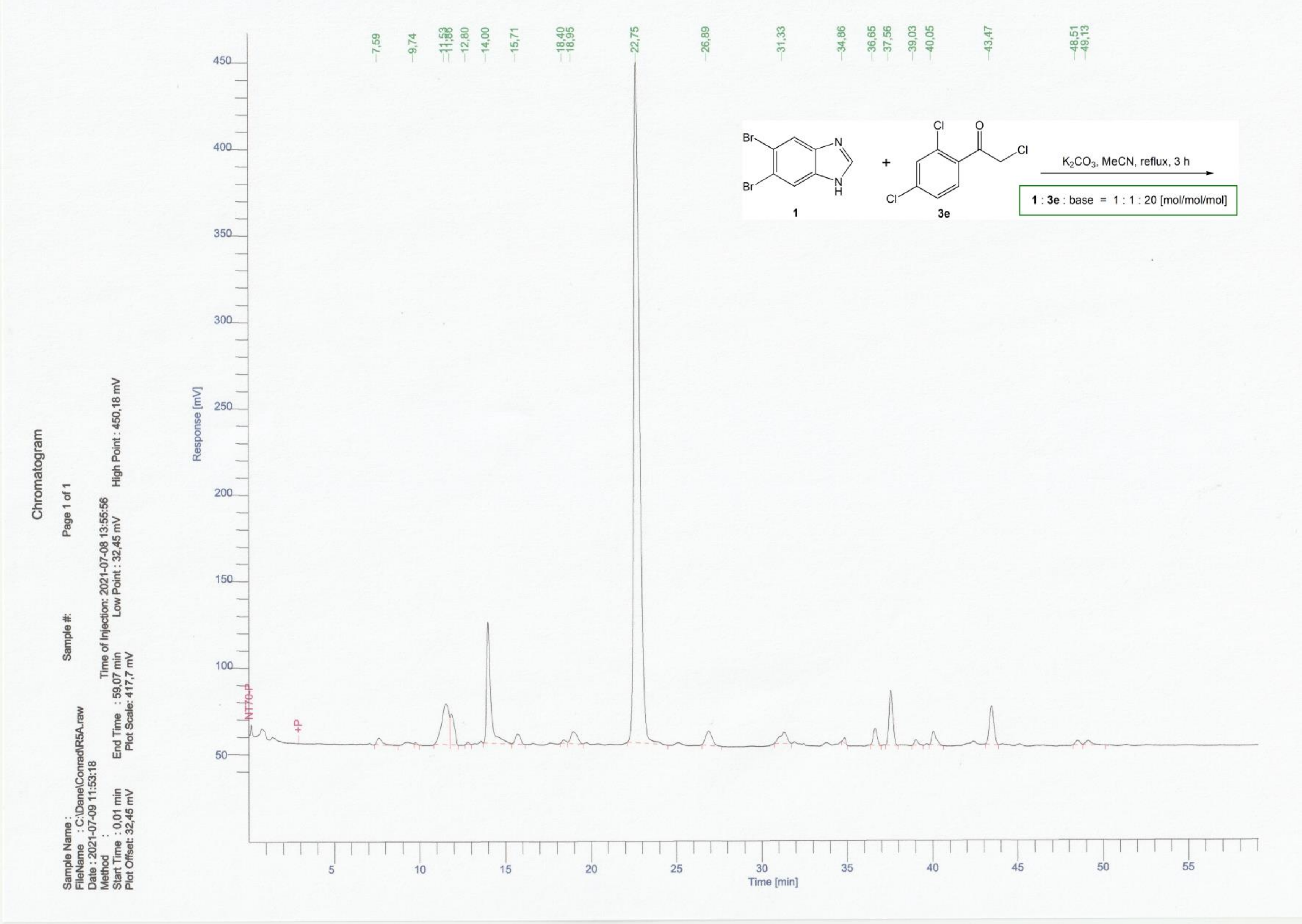

Table 1, Entry 25

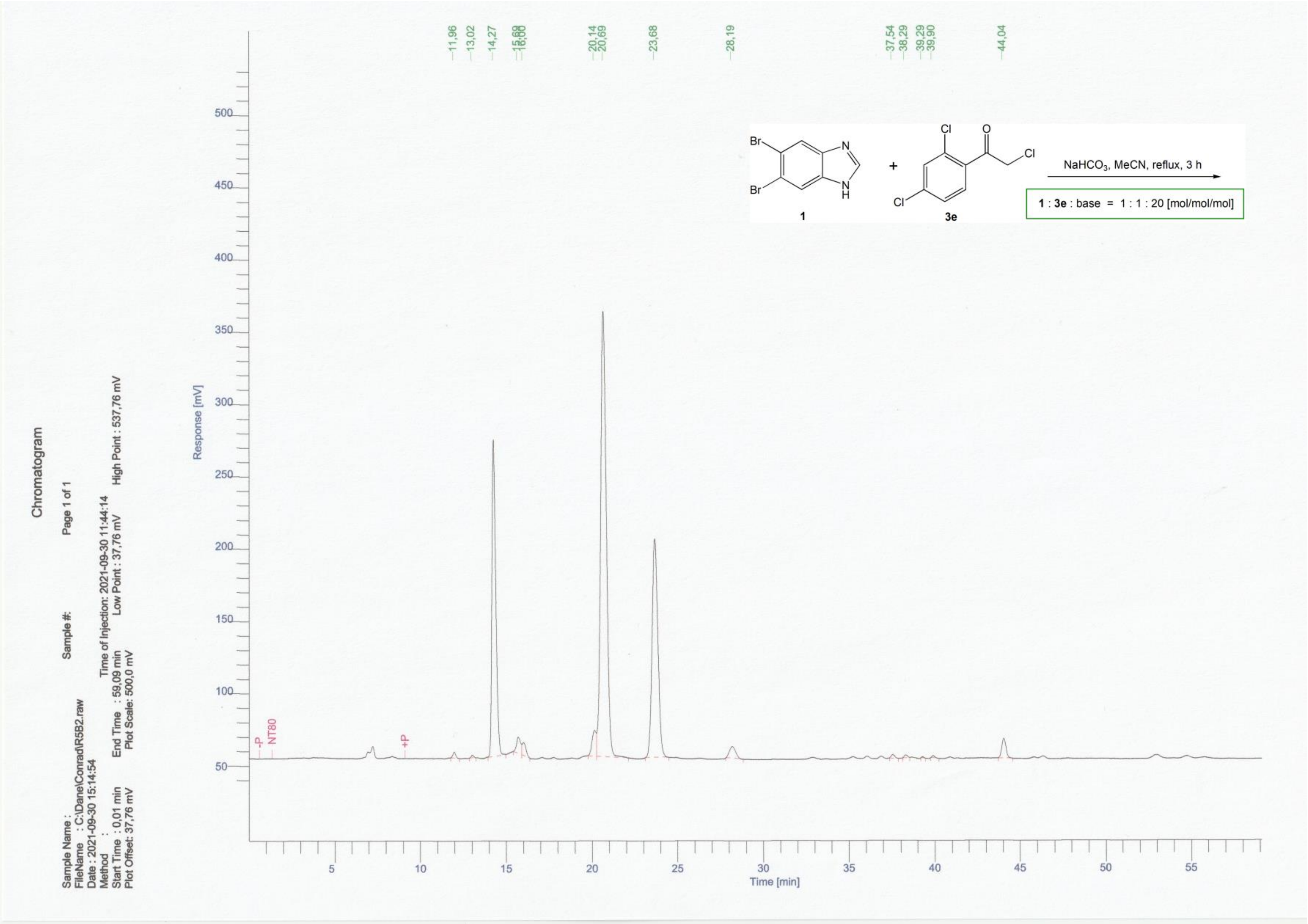

Table 1, Entry 26

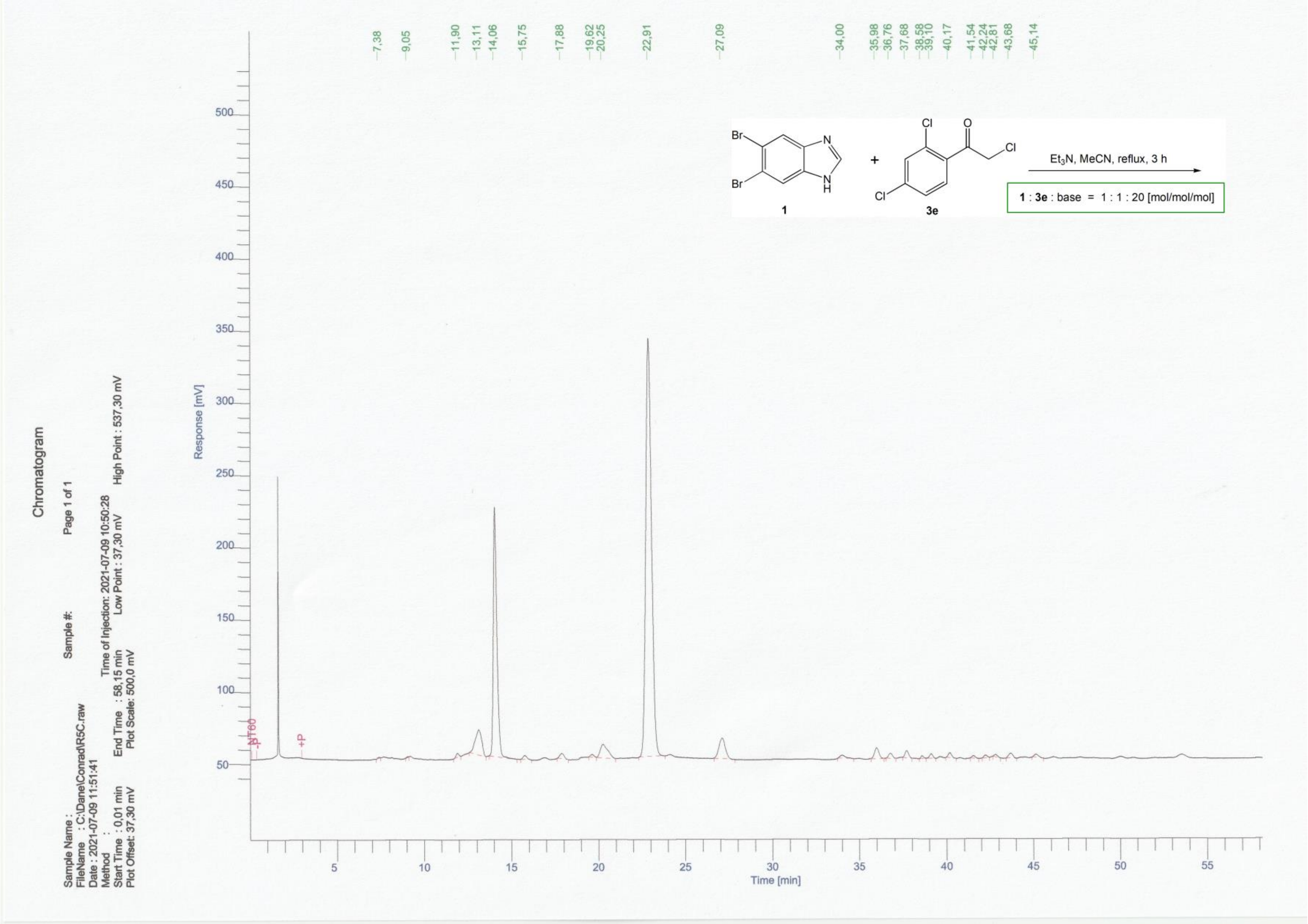

Table 1, Entry 27

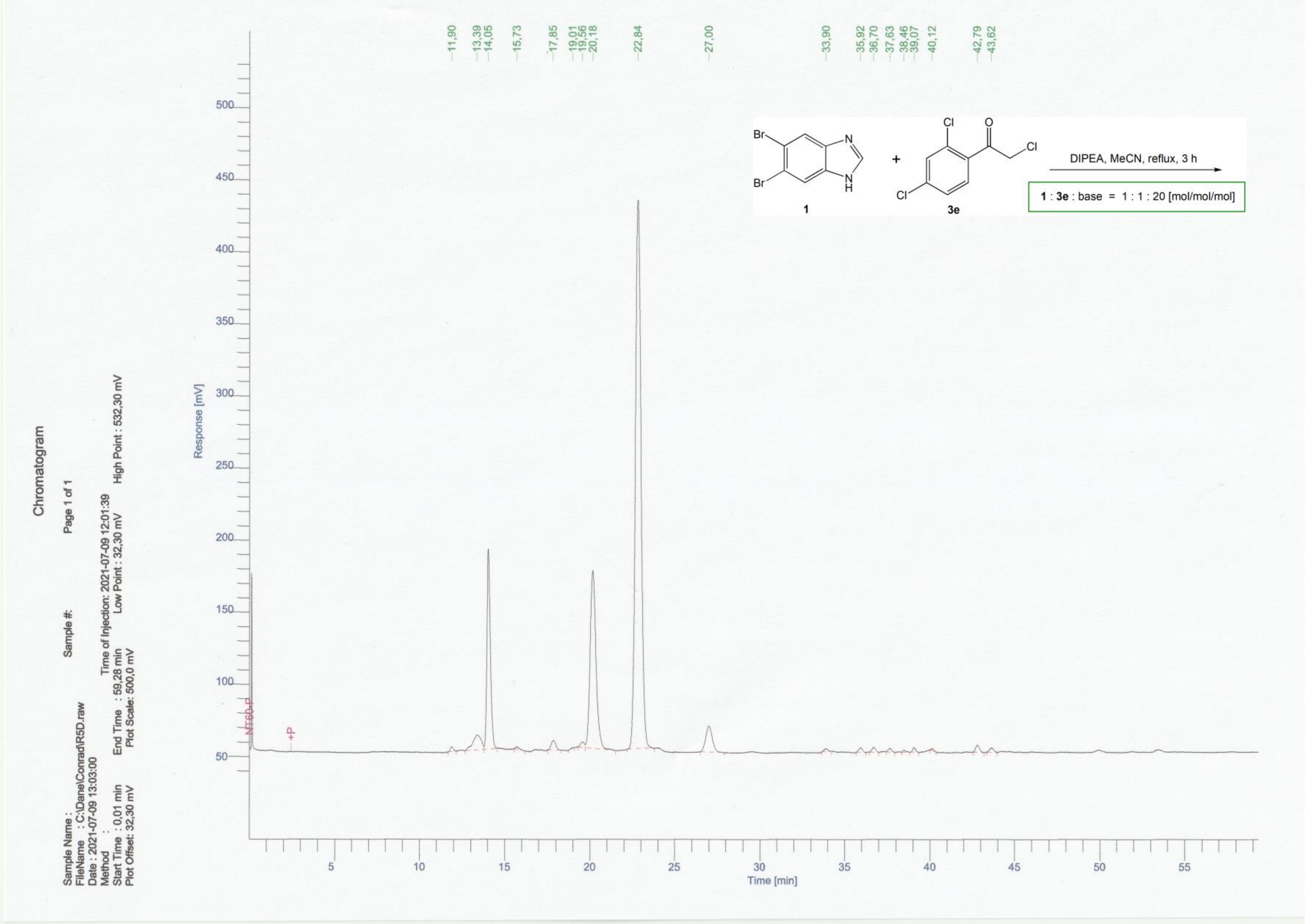

Table 1, Entry 28

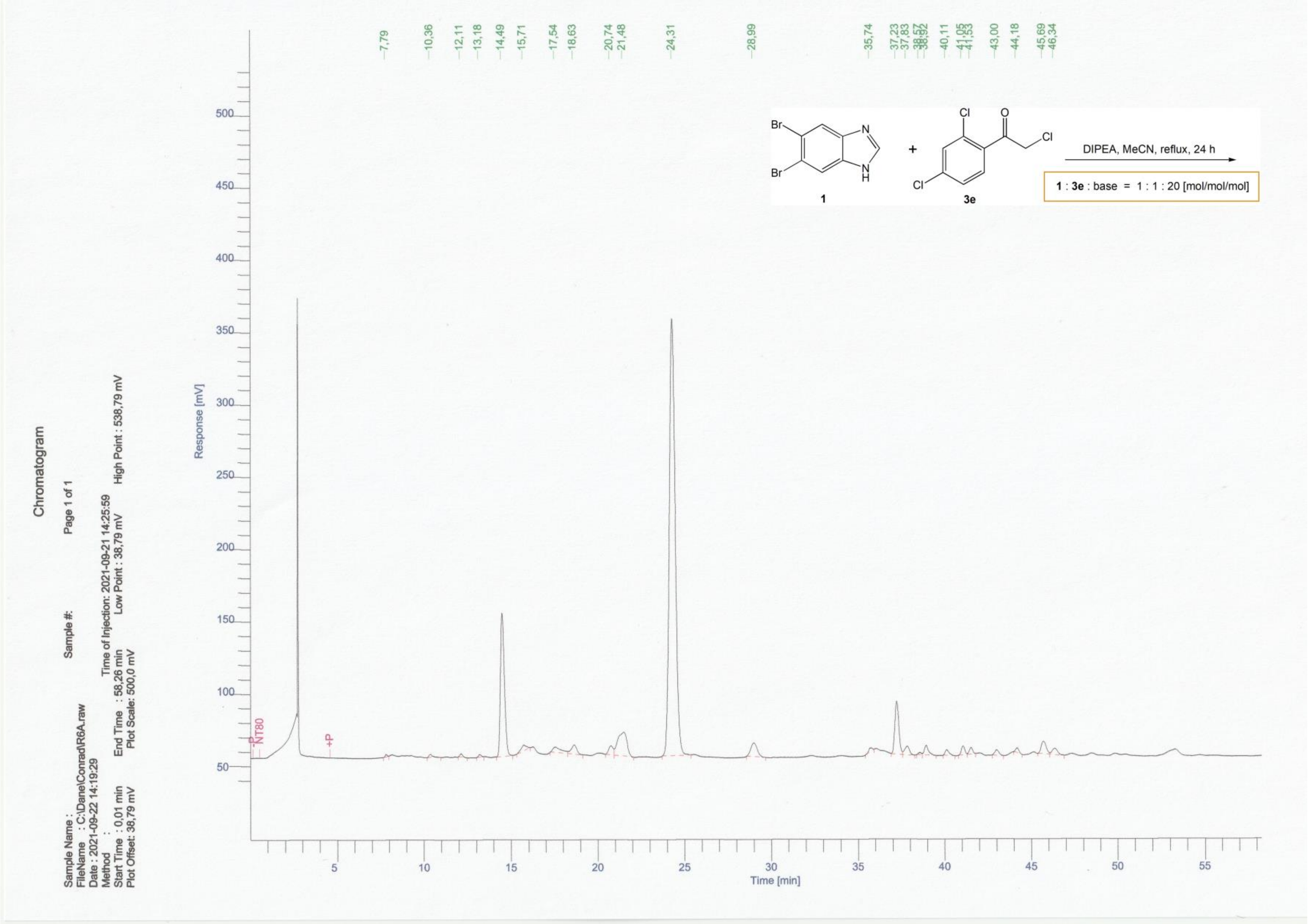

Table 1, Entry 29

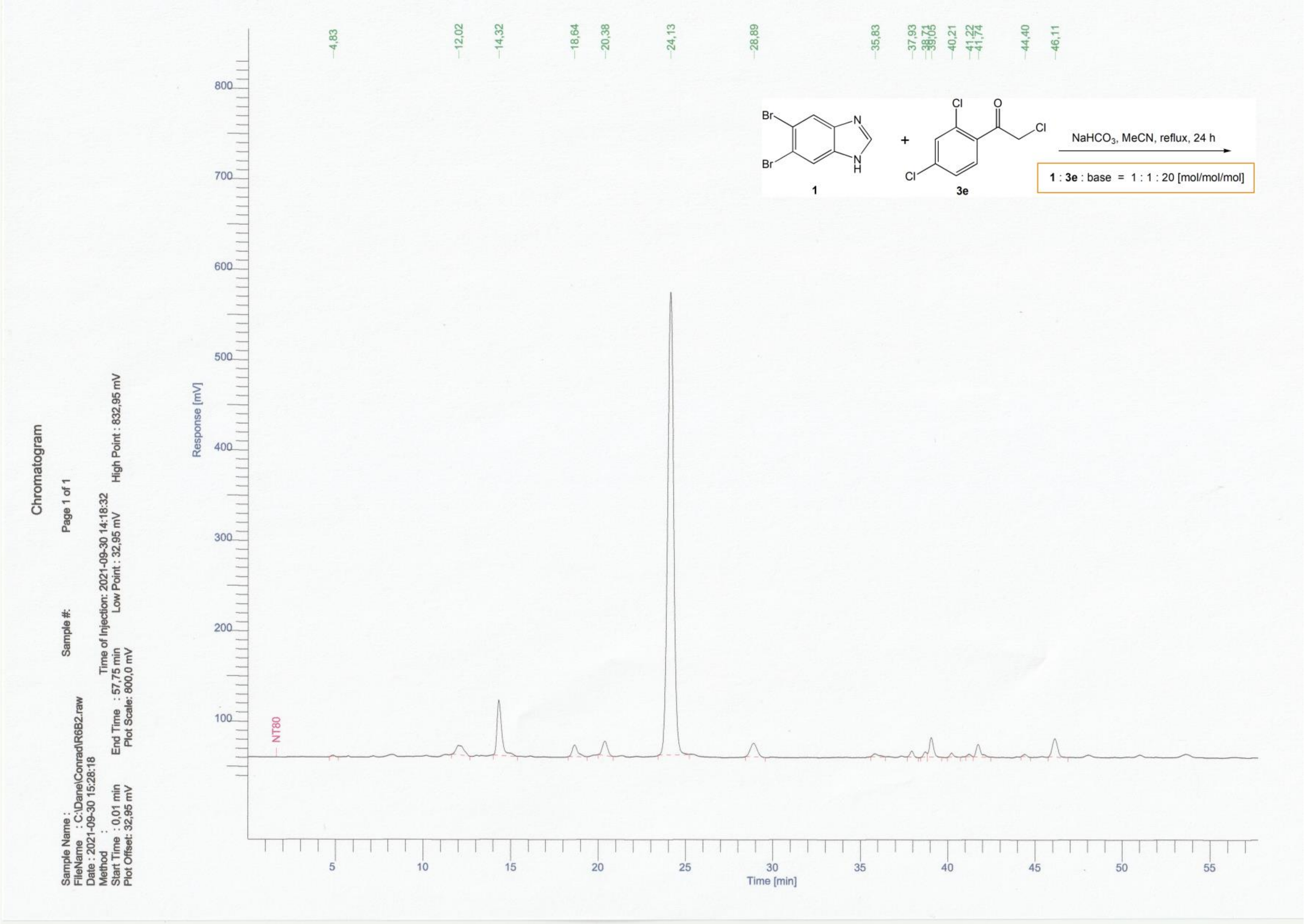

Table 1, Entry 30

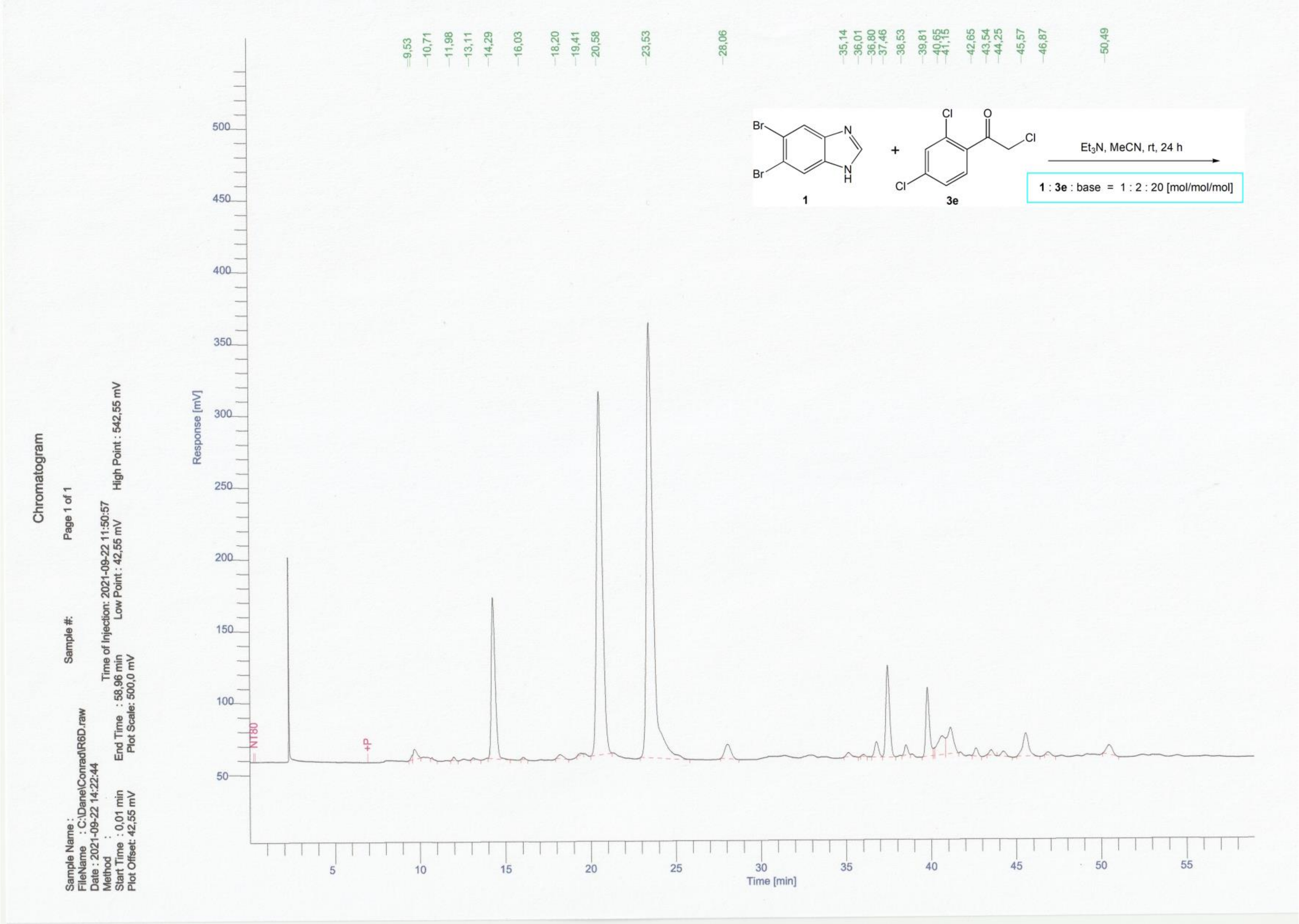

Table 1, Entry 31

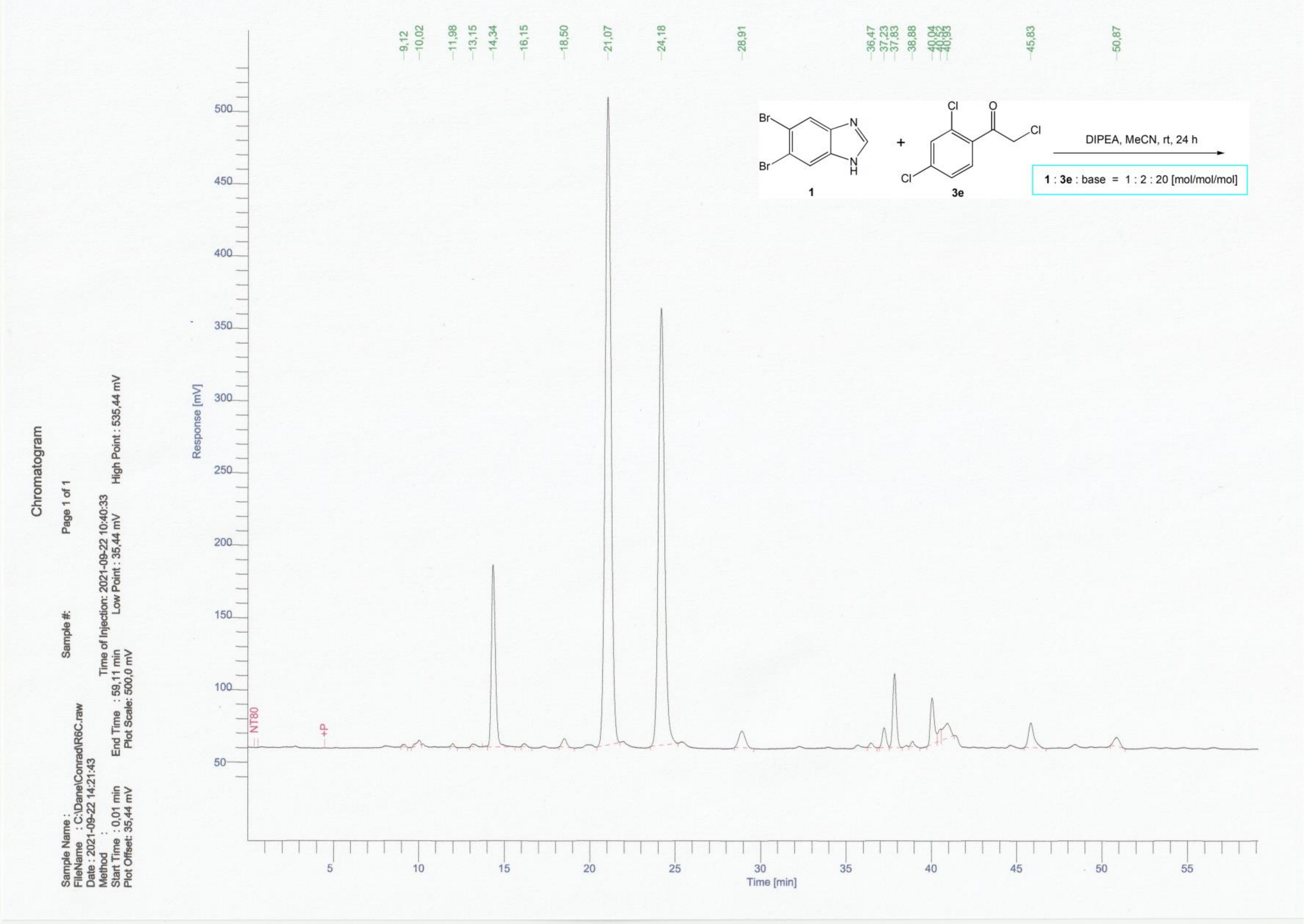

Table 1, Entry 32

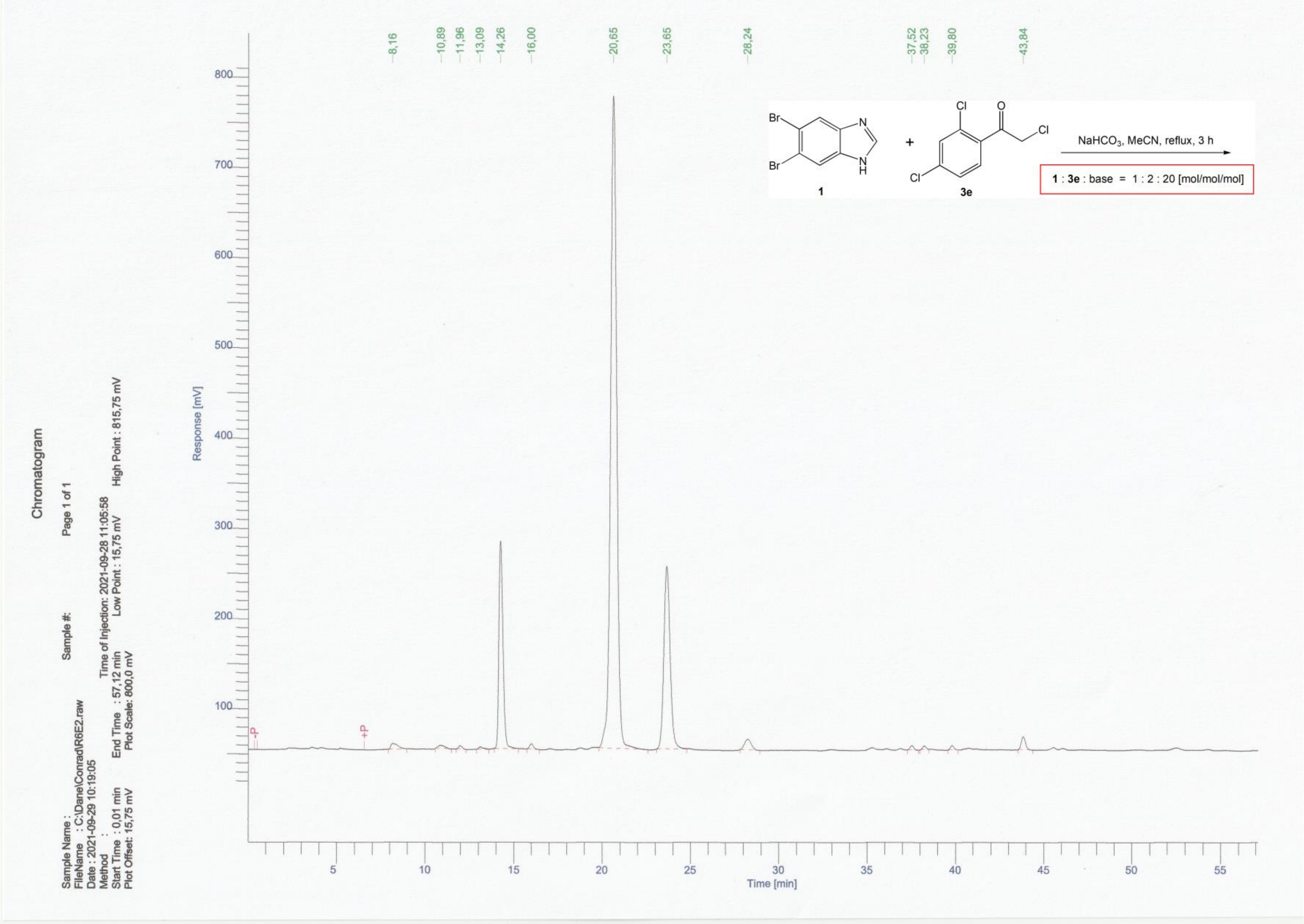

Table 1, Entry 33

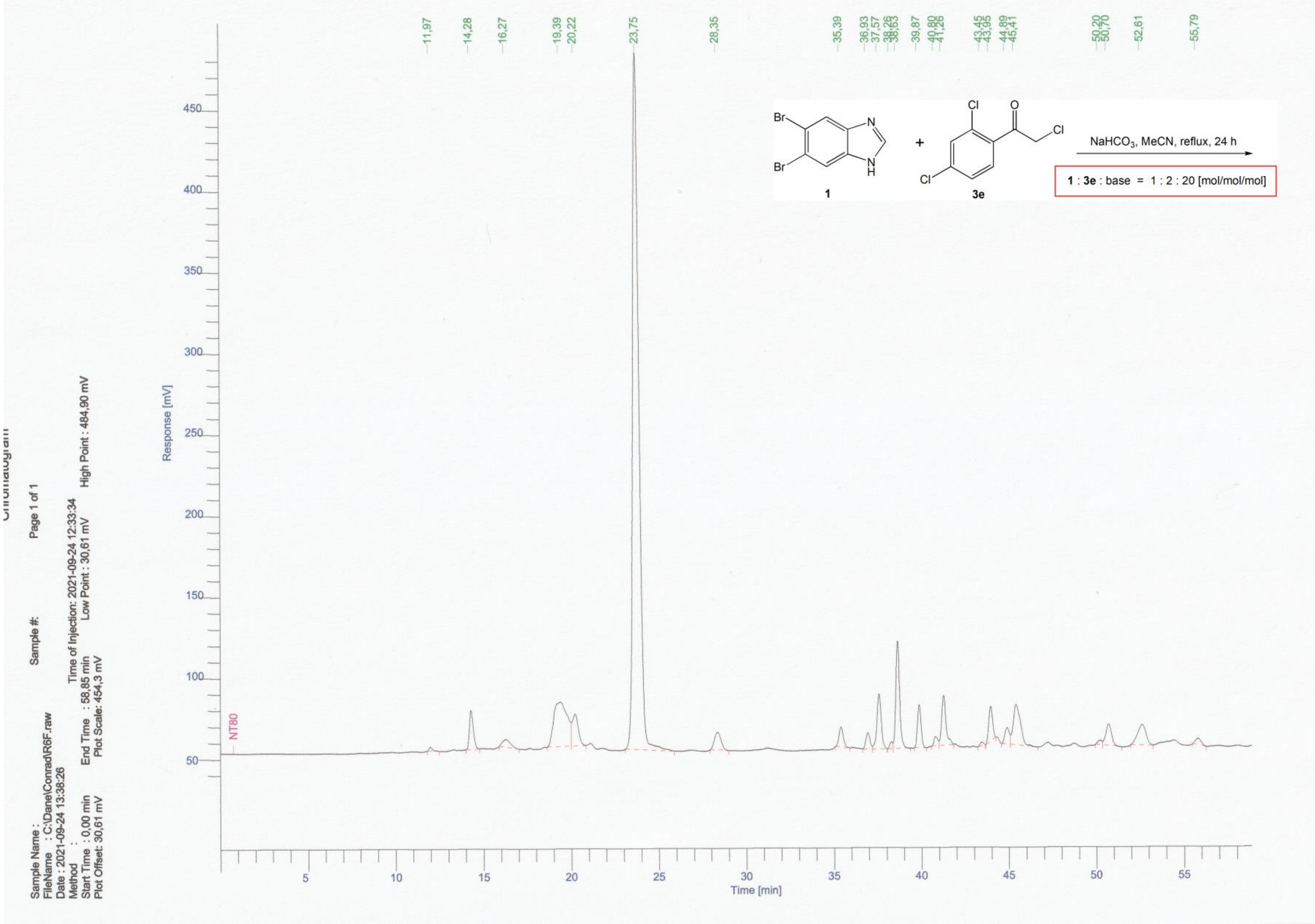

Table 1, Entry 34

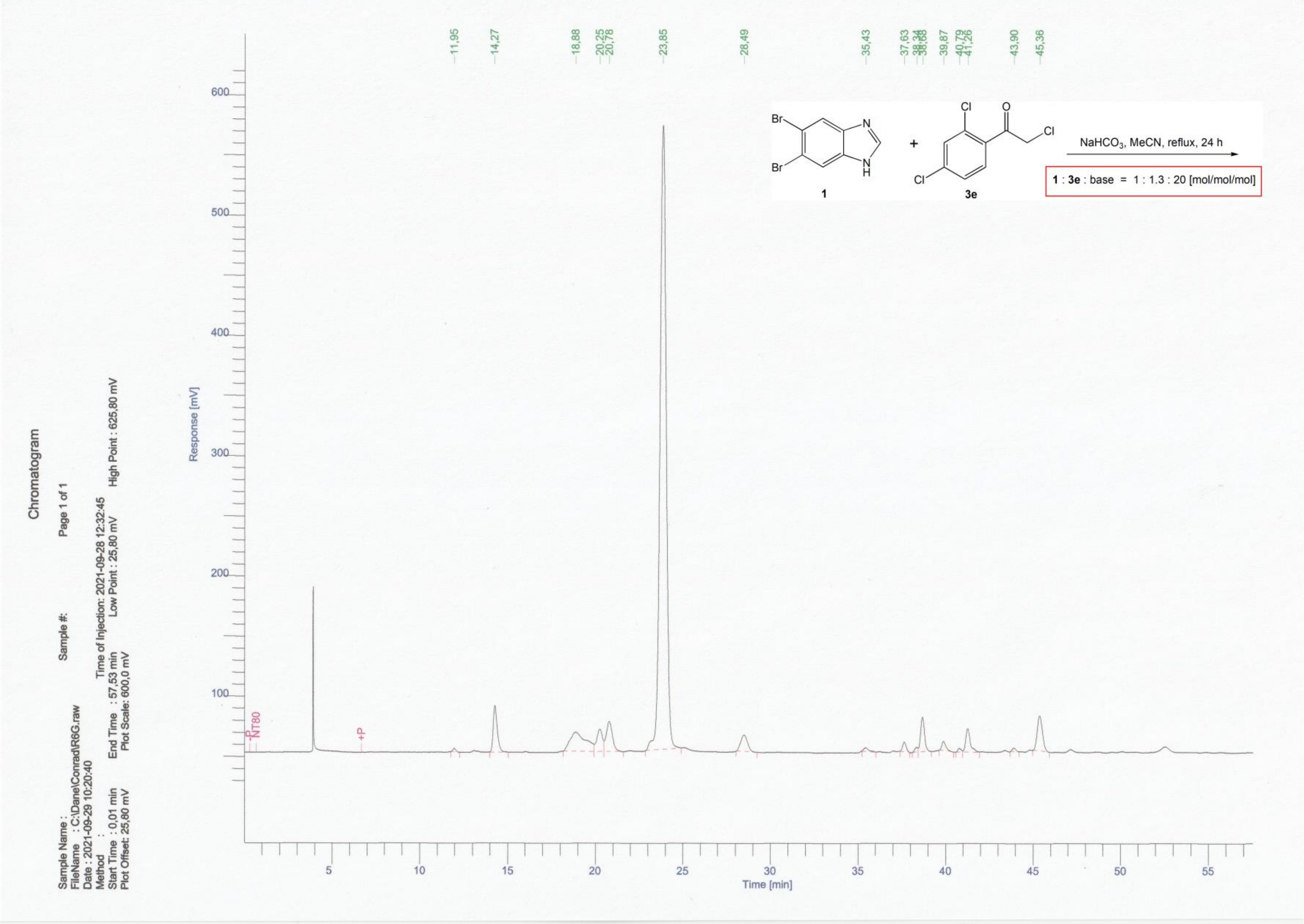

Table 1, Entry 35

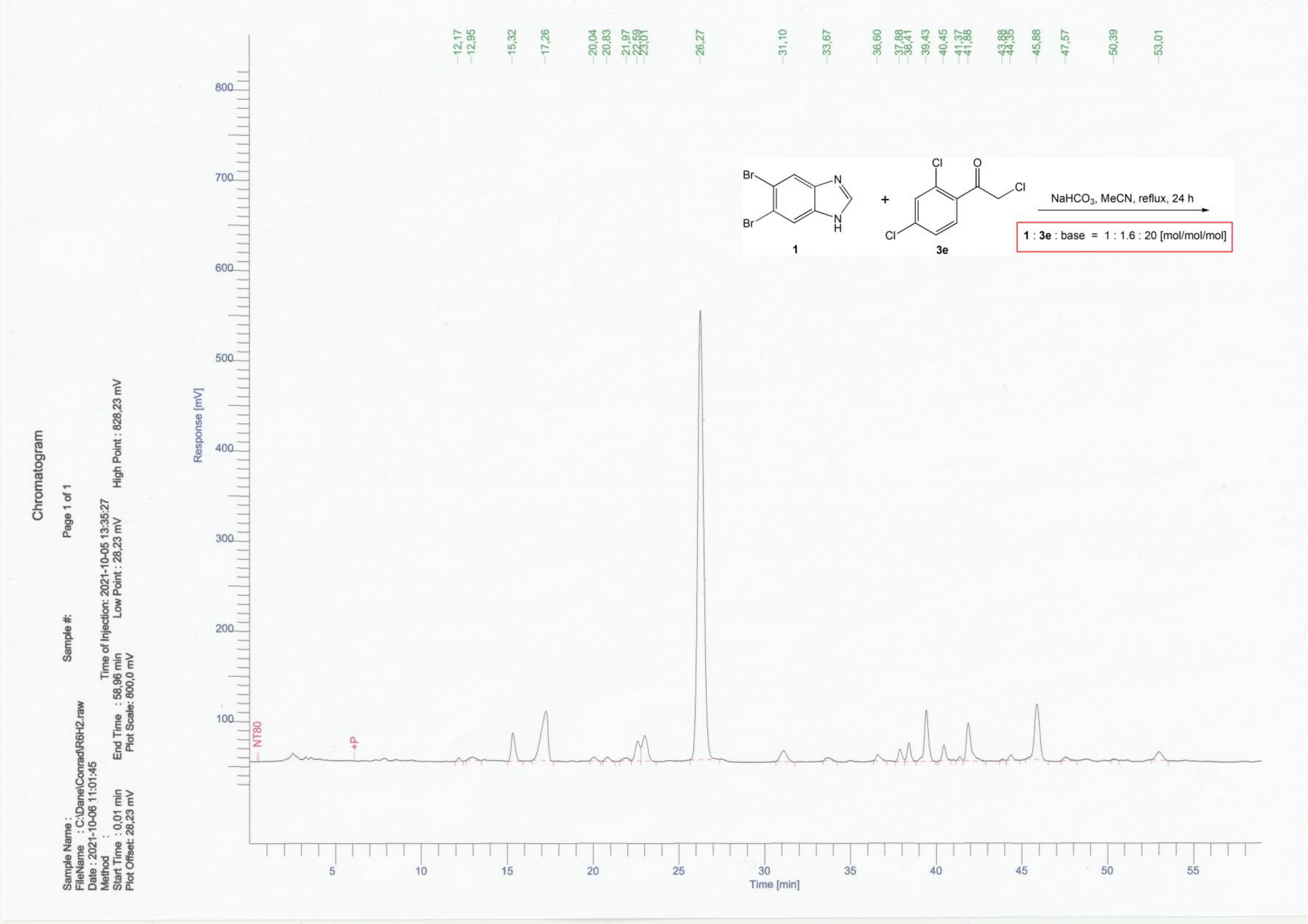

Supplement: Supplementary file 1 [file molecules-27-04349-s001.zip › SUPP INFO _ 1_ HPLC.pdf]
