# Supplementary material for: N-Phenacyldibromobenzimidazoles—Synthesis Optimization and Evaluation of Their Cytotoxic Activity
Source: Molecules. 2022 Jul 7;27(14):4349. doi: 10.3390/molecules27144349 (PMC9315981; doi:10.3390/molecules27144349)
Supplement: Supplementary file 1 [file molecules-27-04349-s001.zip › SUPP INFO _ 2 _ analytical data of compounds and biological tests.pdf]

N-Phenacyldibromobenzimidazoles – synthesis optimization and evaluation of their cytotoxic activity

Anna Kowalkowska[1,*], Konrad Chojnacki[1], Maciej Multan[1], Jan K. Maurin[2], Edyta Łukowska-Chojnacka[1] and Patrycja Wińska[1]

[1] Warsaw University of Technology, Faculty of Chemistry, Noakowskiego St 3, 00-664 Warsaw, Poland, e-mail: anna.kowalkowska@pw.edu.pl, konrad.chojnacki@pw.edu.pl, edyta.chojnacka@pw.edu.pl, patrycja.winska.pw.edu.pl

[2] National Medicines Institute, Chełmska St. 30/34, 00-725 Warsaw, Poland

National Centre for Nuclear Research, Andrzeja Sołtana St. 7, 05-400 Otwock, Poland, e-mail: j.maurin@nil.gov.pl

1. **Isolation of compounds 4 and 5** **2**
2. **Analytical and spectral data of compounds 4 and 5** **4**
3. **^{1}H and ^{13}C NMR spectra of compounds 4 and 5** **9**
4. **HRMS of compounds 4 and 5** **31**
5. **Products of 2,4-dichlorophenacyl chloride 3e self-condensation reaction** **42**
6. **X-ray analysis of compound 5d** **64**
7. **Biological analysis** **66**

1. Isolation of compounds 4 and 5

2-(5,6-dibromo-1*H*-benzimidazol-1-yl)-1-phenylethanone (**4a**): 51% yield (0.80 g, off-white solid), solidified residue treated with Et_2O/MeOH 10/1 v/v.

2-(5,6-dibromo-1*H*-benzimidazol-1-yl)-1-(4-fluorophenyl)ethanone (**4b**): 60% yield (0.99 g, off-white solid), solidified residue treated with Et_2O/MeOH 10/1 v/v.

2-(5,6-dibromo-1*H*-benzimidazol-1-yl)-1-(4-chlorophenyl)ethanone (**4c**): 47% yield (0.81 g, yellowish solid), solidified residue treated with Et_2O/MeOH 10/1 v/v.

2-(5,6-dibromo-1*H*-benzimidazol-1-yl)-1-(4-bromophenyl)ethanone (**4d**): 61% yield (1.15 g, yellowish solid), solidified residue treated with Et_2O/MeOH 10/1 v/v.

2-(5,6-dibromo-1*H*-benzimidazol-1-yl)-1-(2,4-dichlorophenyl)ethanone (**4e**): 67% yield (1.24 g, yellowish solid), solidified residue treated with Et_2O or cold EtOAc (if the residue was very dark).

2-(5,6-dibromo-1*H*-benzimidazol-1-yl)-1-(3,4-dichlorophenyl)ethanone (**4f**): 63% yield (1.17 g, yellowish solid), solidified residue treated with Et_2O/EtOAc 9/1

2-(5,6-dibromo-1*H*-benzimidazol-1-yl)-1-(2,4,6-trichlorophenyl)ethanone (**4g**): dark oily residue purified by column chromatography (silica gel, eluent: chloroform), 41% yield (0.81 g, oil solidifying to yellowish solid).

2-(5,6-dibromo-1*H*-benzimidazol-1-yl)-1-(2,4-difluorophenyl)ethanone (**4h**): dark oily residue purified by column chromatography (silica gel, eluent: chloroform), 48% yield (0.83 g, light brown oil, solidifying after treating with acetone), solid product filtered and washed with Et_2O (off-white solid).

2-(5,6-dibromo-1*H*-benzimidazol-1-yl)-1-(2,5-difluorophenyl)ethanone (**4i**): 65% yield (1.12 g, pale beige solid), solidified residue treated with Et_2O.

2-(5,6-dibromo-1*H*-benzimidazol-1-yl)-1-(2,4,6-trifluorophenyl)ethanone (**4j**): dark oily residue purified by column chromatography (silica gel, eluent: chloroform), 54% yield (0.97 g, yellowish solid).

2-(4,6-dibromo-1*H*-benzimidazol-1-yl)-1-phenylethanone (**5a**): 82% (1.29 g, off-white solid), solidified residue treated with Et_2O/MeOH 10/1 v/v.

2-(4,6-dibromo-1*H*-benzimidazol-1-yl)-1-(4-fluorophenyl)ethanone (**5b**): 89% yield (1.47 g, off-white solid), solidified residue treated with Et_2O/MeOH 10/1 v/v.

2-(4,6-dibromo-1*H*-benzimidazol-1-yl)-1-(4-chlorophenyl)ethanone (**5c**): 83% yield (1.41 g, off-white solid), solidified residue treated with Et_2O/MeOH 10/1 v/v.

2-(4,6-dibromo-1*H*-benzimidazol-1-yl)-1-(4-bromophenyl)ethanone (**5d**): 84% yield (1.59 g, yellowish solid), solidified residue treated with Et_2O/MeOH 10/1 v/v.

2-(4,6-dibromo-1*H*-benzimidazol-1-yl)-1-(2,4-dichlorophenyl)ethanone (**5e**): 80% yield (1.48 g, yellow solid), solidified residue treated with Et_2O or cold EtOAc (if the residue was very dark).

2-(4,6-dibromo-1*H*-benzimidazol-1-yl)-1-(3,4-dichlorophenyl)ethanone (**5f**): 77% yield (1.43 g, yellow solid), solidified residue treated with Et_2O/acetone 6/1 (v/v).

2-(4,6-dibromo-1*H*-benzimidazol-1-yl)-1-(2,4,6-trichlorophenyl)ethanone (**5g**): dark oily residue purified by column chromatography (silica gel, eluent: chloroform), 39% yield (0.78 g, oil solidifying to yellowish solid).

2-(4,6-dibromo-1*H*-benzimidazol-1-yl)-1-(2,4-difluorophenyl)ethanone (**5h**): 74% yield (1.27 g, off-white solid), solidified residue treated with Et_2O.

2-(4,6-dibromo-1*H*-benzimidazol-1-yl)-1-(2,5-difluorophenyl)ethanone (**5i**): 59% yield (1.01 g, off-white solid), solidified residue treated with Et_2O.

2-(4,6-dibromo-1*H*-benzimidazol-1-yl)-1-(2,4,6-trifluorophenyl)ethanone (**5j**): dark oily residue purified by column chromatography (silica gel, eluent: chloroform), 57% yield (1.02 g, light brown oil, solidifying after treating with acetone), solid product filtered and washed with Et_2O (off-white solid).

2. Analytical and spectral data of compounds 4 and 5

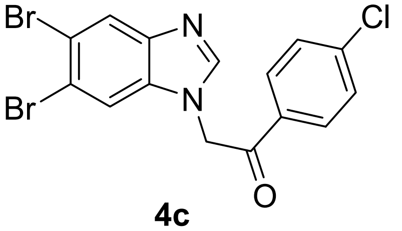

4c

2-(5,6-dibromo-1*H*-benzimidazol-1-yl)-1-(4-chlorophenyl)ethanone (**4c**): white crystals, m.p. = 188-189 °C (MeOH); ^{1}H NMR (500 MHz, DMSO-d_6) δ = 6.04 (s, 2H, CH_2), 7.70-7.73 (m, 2H, C_6H_4), 8.08-8.11 (m, 2H, C_6H_4 and 1H, CH=N), 8.20 (s, 1H, C_6H_2), 8.25 (s, 1H, C_6H_2); ^{13}C NMR (125 MHz, DMSO-d_6) δ = 51.21, 115.83, 116.75, 123.64, 128.97, 130.07, 133.06, 135.18, 138.86, 143.59, 147.21, 192.07; HRMS: calculated for $C_{15}H_{10}Br_2ClN_2O$ $[M+H]^+$: 426.88429, 428.88225, 430.88020. Found: 426.88530, 428.88283, 430.88071.

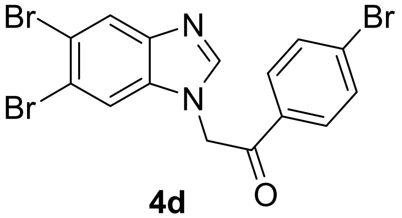

4d

2-(5,6-dibromo-1*H*-benzimidazol-1-yl)-1-(4-bromophenyl)ethanone (**4d**): off-white crystals, m.p. = 211-212 °C (MeOH); ^{1}H NMR (500 MHz, DMSO-d_6) δ = 6.03 (s, 2H, CH_2), 7.85-7.86 (m, 2H, C_6H_4), 8.00-8.02 (m, 2H, C_6H_4), 8.10 (s, 1H, CH=N), 8.20 (s, 1H, C_6H_2), 8.25 (s, 1H, C_6H_2); ^{13}C NMR (125 MHz, DMSO-d_6) δ = 51.18, 115.83, 116.75, 123.62, 128.07, 130.13, 131.92, 133.37, 135.17, 143.59, 147.21, 192.28; HRMS: calculated for $C_{15}H_{10}Br_3N_2O$ $[M+H]^+$: 470.83378, 472.83173, 474.82968. Found: 470.83430, 472.83201, 474.82978.

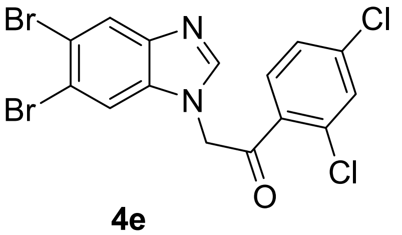

4e

2-(5,6-dibromo-1*H*-benzimidazol-1-yl)-1-(2,4-dichlorophenyl)ethanone (**4e**): yellowish crystals, m.p. = 193-194 °C (EtOH); ^{1}H NMR (500 MHz, DMSO-d_6) δ = 5.93 (s, 2H, CH_2), 7.71 (dd, J = 8.31 Hz, J = 1.96 Hz, 1H, C_6H_3), 7.85 (d, J = 1.96 Hz, 1H, C_6H_3), 8.11 (s, 1H, CH=N), 8.11 (d, J = 8.31 Hz, 1H, C_6H_3), 8.17 (s, 1H, C_6H_2), 8.27 (s, 1H, C_6H_2); ^{13}C NMR (125 MHz, DMSO-d_6) δ

= 53.20, 115.77, 115.98, 116.85, 123.70, 127.62, 130.60, 131.83, 132.40, 133.17, 134.93, 137.51, 143.58, 147.08, 193.29; HRMS: calculated for $C_{15}H_9Br_2Cl_2N_2O$ $[M+H]^+$: 460.84532, 462.84327, 464.84032. Found: 460.84566, 462.84318, 464.84054.

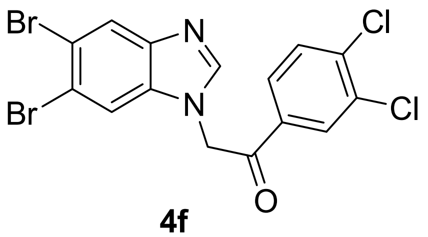

4f

2-(5,6-dibromo-1*H*-benzimidazol-1-yl)-1-(3,4-dichlorophenyl)ethanone (**4f**): white crystals, m.p. = 212-213 °C (EtOH); ^1H NMR (500 MHz, DMSO-d_6) δ = 6.05 (s, 2H, CH_2), 7.92 (d, J = 8.31 Hz, 1H, C_6H_3), 8.02 (dd, J = 8.31 Hz, J = 1.96 Hz, 1H, C_6H_3), 8.11 (s, 1H, CH=N), 8.21 (s, 1H, C_6H_2), 8.25 (s, 1H, C_6H_2), 8.32 (d, J = 1.96 Hz, 1H, C_6H_3); ^{13}C NMR (125 MHz, DMSO-d_6) δ = 51.37, 115.85, 115.93, 116.83, 123.67, 128.15, 130.17, 131.24, 131.90, 134.60, 135.13, 136.72, 143.58, 147.15, 191.47; HRMS: calculated for $C_{15}H_9Br_2Cl_2N_2O$ $[M+H]^+$: 460.84532, 462.84327, 464.84032. Found: 460.84585, 462.84342, 464.84097.

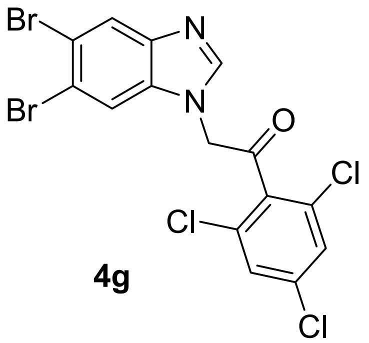

4g

2-(5,6-dibromo-1*H*-benzimidazol-1-yl)-1-(2,4,6-trichlorophenyl)ethanone (**4g**): off white crystals, m.p. = 197-198 °C (EtOH); ^1H NMR (500 MHz, DMSO-d_6) δ = 5.87 (s, 2H, CH_2), 7.93 (s, 2H, C_6H_2), 7.97 (s, 1H, CH=N), 8.14 (s, 1H, $C_6H_2Br_2$), 8.36 (s, 1H, $C_6H_2Br_2$); ^{13}C NMR (125 MHz, DMSO-d_6) δ = 53.86, 115.37, 116.30, 117.04, 124.00, 128.61, 130.94, 134.47, 134.84, 136.36, 143.63, 146.99, 195.89; HRMS: calculated for $C_{15}H_8Br_2Cl_3N_2O$ $[M+H]^+$: 494.80635, 496.80430, 498.80135. Found: 494.80759, 496.80500, 498.80239.

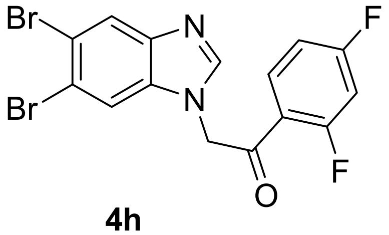

4h

2-(5,6-dibromo-1*H*-benzimidazol-1-yl)-1-(2,4-difluorophenyl)ethanone (**4h**): white crystals, m.p. = 135-136 °C (EtOH); ^{1}H NMR (500 MHz, DMSO-*d$_6$*) δ= 5.85 (d, *J* = 3.42 Hz, 2H, CH_2), 7.32 (dd, *J* = 8.31 Hz, *J* = 2.45 Hz, 1H, C_6H_3), 7.55-7.60 (m, 1H, C_6H_3), 8.04-8.10 (m, 1H, C_6H_3), 8.09 (s, 1H, CH=N), 8.21 (s, 1H, C_6H_2), 8.23 (s, 1H, C_6H_2); ^{13}C NMR (125 MHz, DMSO-*d$_6$*) δ= 54.01 (d, *J* = 11.74 Hz), 105.08-105.50 (m, 3 lines), 112.66 (dd, *J* = 22.50 Hz, *J* = 2.93 Hz), 115.84, 116.01, 116.75, 119.75 (dd, *J* = 13.69 Hz, *J* = 3.91 Hz), 123.59, 132.57-132.70 (m, 4 lines), 135.19, 143.61, 147.19, 161.73, 162.71 (dd, *J* = 258.23 Hz, *J* = 13.69 Hz), 165.70 (dd, *J* = 155.30 Hz, *J* = 12.72 Hz), 189.49 (d, *J* = 5.87 Hz); HRMS: calculated for $C_{15}H_9Br_2F_2N_2O$ $[M+H]^+$: 428.90442, 430.90237, 432.90033. Found: 428.90485, 430.90257, 432.90023.

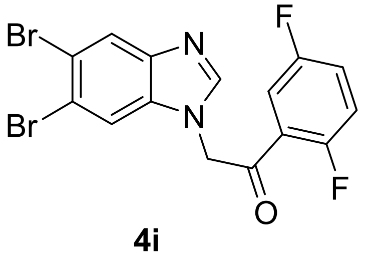

4i

2-(5,6-dibromo-1*H*-benzimidazol-1-yl)-1-(2,5-difluorophenyl)ethanone (**4i**): yellowish crystals, m.p. = 187-188 °C (EtOH); ^{1}H NMR (500 MHz, DMSO-*d$_6$*) δ= 5.87 (d, *J* = 3.42 Hz, 2H, CH_2), 7.56-7.60 (m, 1H, C_6H_3), 7.65-7.70 (m, 1H, C_6H_3), 7.72-7.76 (m, 1H, C_6H_3), 8.10 (s, 1H, CH=N), 8.23 (s, 1H, C_6H_2), 8.23 (s, 1H, C_6H_2); ^{13}C NMR (125 MHz, DMSO-*d$_6$*) δ= 54.06 (d, *J* = 11.74 Hz), 115.83-116.06 (m, 4 lines), 116.77, 118.95-119.23 (m, 4 lines), 122.72 (dd, *J* = 24.45 Hz, *J* = 9.78 Hz), 123.59, 123.92 (dd, *J* = 16.63 Hz, *J* = 6.85 Hz), 135.13, 143.61, 147.13, 156.94-159.04 (m, 6 lines), 189.78-189.83 (m, 3 lines); HRMS: calculated for $C_{15}H_9Br_2F_2N_2O$ $[M+H]^+$: 428.90442, 430.90237, 432.90033. Found: 428.90438, 430.90256, 432.90029.

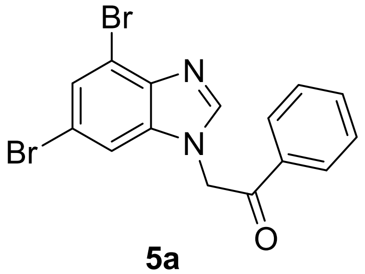

5a

2-(4,6-dibromo-1*H*-benzimidazol-1-yl)-1-phenylethanone (**5a**): white crystals, m.p. = 211-212 °C (MeOH); ^{1}H NMR (500 MHz, DMSO-*d6*) δ = 6.07 (s, 2H, CH_2), 7.62-7.65 (m, 2H, C_6H_5), 7.64 (d, *J* = 1.71 Hz, 1H, C_6H_2), 7.73-7.77 (m, 1H, C_6H_5), 8.02 (d, *J* = 1.71 Hz, 1H, C_6H_2), 8.08-8.10 (m, 2H, C_6H_5), 8.31 (s, 1H, CH=N). ^{13}C NMR (125 MHz, DMSO-*d6*) δ = 51.48, 113.22, 113.78, 114.90, 126.53, 128.22, 128.90, 134.10, 134.31, 136.32, 140.88, 146.62, 192.87. HRMS: calculated for $C_{15}H_{11}Br_2N_2O$ $[M+H]^+$: 392.92326, 394.92122, 396.91917. Found: 392.92422, 394.92190, 396.92023.

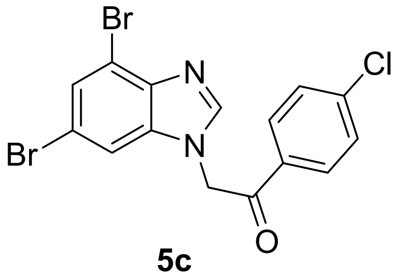

5c

2-(4,6-dibromo-1*H*-benzimidazol-1-yl)-1-(4-chlorophenyl)ethanone (**5c**): white crystals, m.p. = 228-230 °C (MeOH); ^{1}H NMR (500 MHz, DMSO-*d6*) δ = 6.05 (s, 2H, CH_2), 7.64 (d, *J* = 1.71 Hz, 1H, C_6H_2), 7.70-7.73 (m, 2H, C_6H_4), 8.02 (d, *J* = 1.47 Hz, 1H, C_6H_2), 8.08-8.11 (m, 2H, C_6H_4), 8.30 (s, 1H, CH=N); ^{13}C NMR (125 MHz, DMSO-*d6*) δ = 51.75, 112.67, 114.08, 115.35, 127.08, 129.04, 130.16, 133.00, 136.02, 139.00, 139.70, 146.41, 191.85; HRMS: calculated for $C_{15}H_{10}Br_2ClN_2O$ $[M+H]^+$: 426.88429, 428.88225, 430.88020. Found: 426.88505, 428.88227, 430.88039.

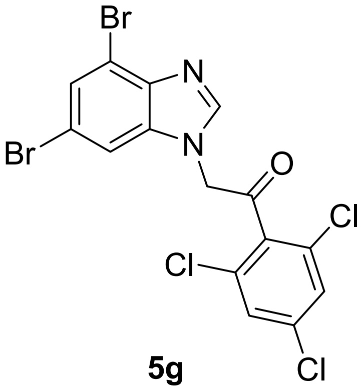

5g

2-(4,6-dibromo-1*H*-benzimidazol-1-yl)-1-(2,4,6-trichlorophenyl)ethanone (**5g**): off white crystals, m.p. = 212-214 °C (EtOH); ^{1}H NMR (500 MHz, DMSO-d_6) δ = 5.89 (s, 2H, CH_2), 7.69 (d, *J* = 1.51 Hz, 1H, $C_6H_2Br_2$), 7.78 (d, *J* = 1.67 Hz, 1H, $C_6H_2Br_2$), 7.94 (s, 2H, C_6H_2), 8.40 (s, 1H, CH=N); ^{13}C NMR (125 MHz, DMSO-d_6) δ = 54.09, 113.31, 113.59, 115.10, 126.97, 128.63, 130.97, 134.80, 135.53, 136.40, 140.93, 146.29, 195.75; HRMS: calculated for $C_{15}H_8Br_2Cl_3N_2O$ $[M+H]^+$: 494.80635, 496.80430, 498.80135. Found: 494.80770, 496.80501, 498.80238.

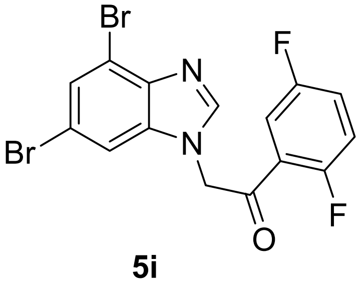

5i

2-(4,6-dibromo-1*H*-benzimidazol-1-yl)-1-(2,5-difluorophenyl)ethanone (**5i**): off white crystals, m.p. = 134-135 °C (EtOH); ^{1}H NMR (500 MHz, DMSO-d_6) δ = 5.88 (d, *J* = 3.42 Hz, 2H, CH_2), 7.56-7.61 (m, 1H, C_6H_3), 7.64 (d, *J* = 1.71 Hz, 1H, C_6H_2), 7.65-7.70 (m, 1H, C_6H_3), 7.72-7.76 (m, 1H, C_6H_3), 8.05 (d, *J* = 1.71 Hz, 1H, C_6H_2), 8.27 (s, 1H, CH=N); ^{13}C NMR (125 MHz, DMSO-d_6) δ = 54.29 (d, *J* = 10.76 Hz), 113.14, 113.93, 114.89, 115.84-116.07 (m, 4 lines), 118.96-119.24 (m, 4 lines), 122.62-122.89 (m, 4 lines), 123.88 (dd, *J* = 16.63 Hz, *J* = 6.85 Hz), 126.52, 136.21, 140.84, 146.42, 156-95-159.04 (m, 7 lines), 189.66-189.72 (m, 3 lines); HRMS: calculated for $C_{15}H_9Br_2F_2N_2O$ $[M+H]^+$: 428.90442, 430.90237, 432.90033. Found: 428.90480, 430.90257, 432.90029.

3. ^{1}H and ^{13}C NMR spectra of compounds 4 and 5

2-(5,6-dibromo-1*H*-benzimidazol-1-yl)-1-(4-chlorophenyl)ethanone (4c)

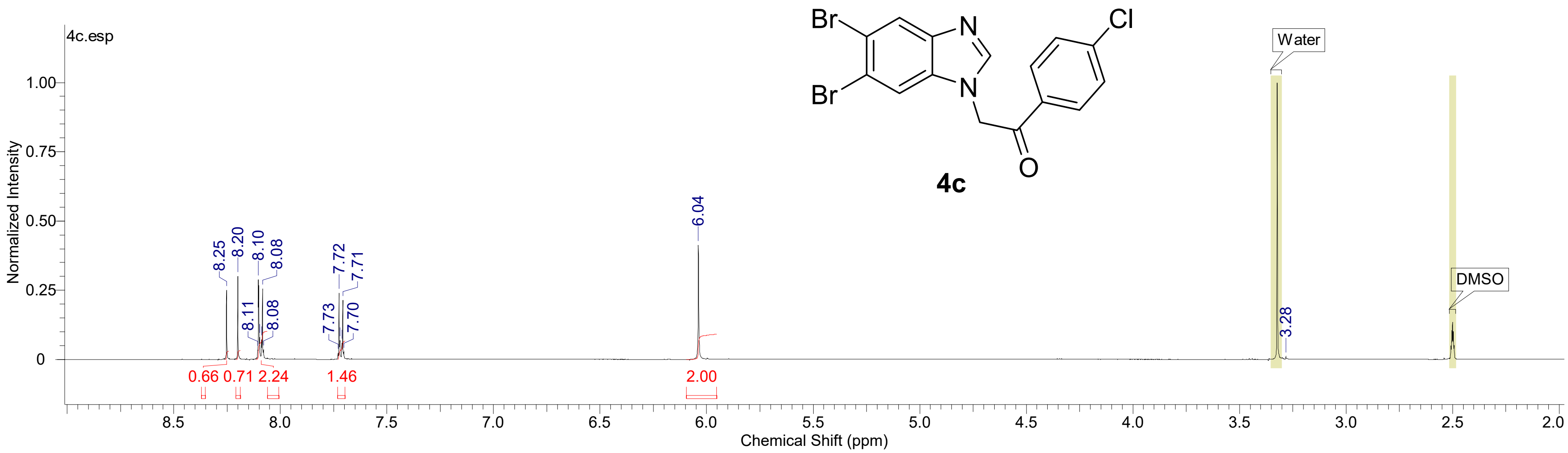

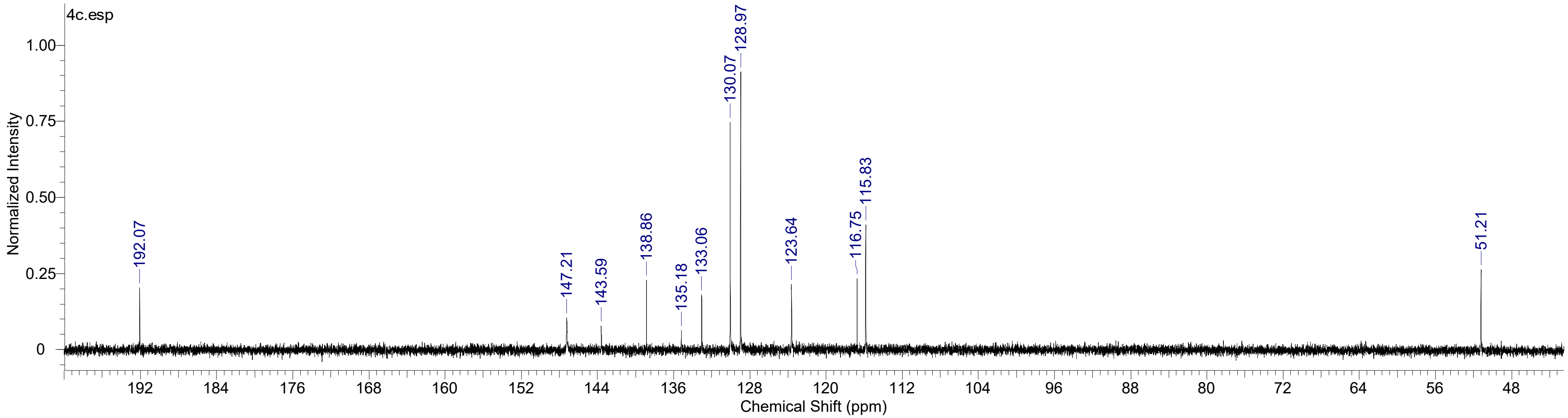
4c.esp
Normalized Intensity
Chemical Shift (ppm)
192.07
147.21
143.59
138.86
135.18
133.06
130.07
128.97
123.64
116.75
115.83
51.21

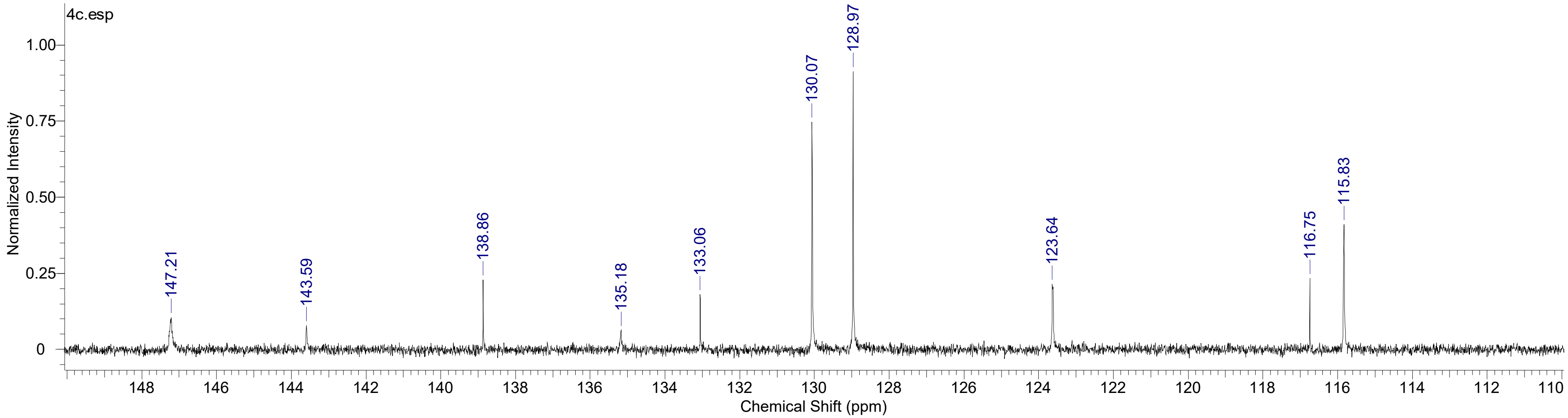
4c.esp
Normalized Intensity
Chemical Shift (ppm)
147.21
143.59
138.86
135.18
133.06
130.07
128.97
123.64
116.75
115.83

2-(5,6-dibromo-1*H*-benzimidazol-1-yl)-1-(4-bromophenyl)ethanone (4d)

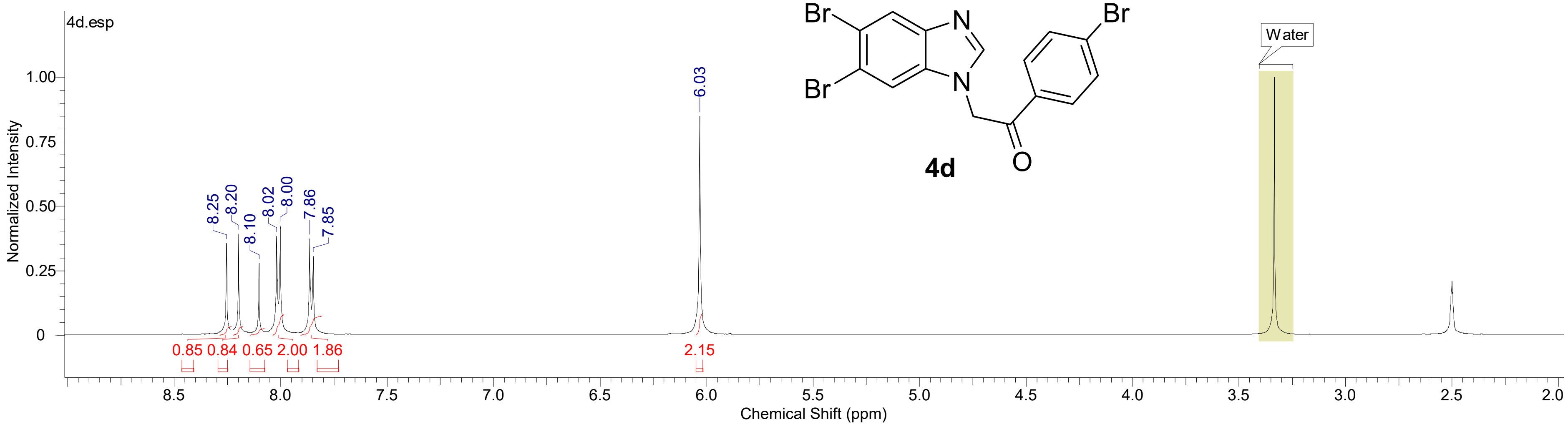

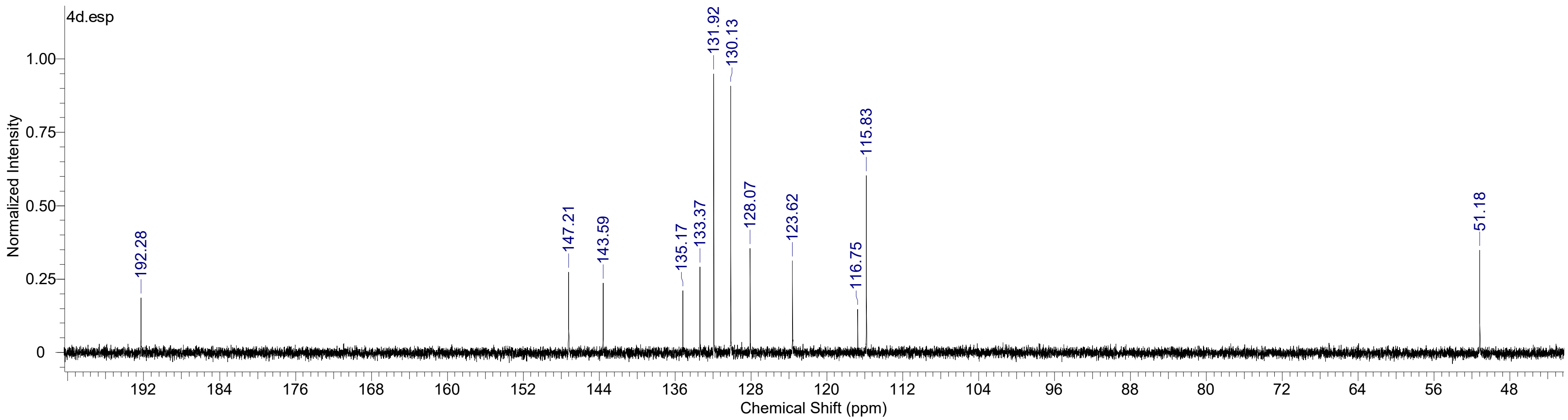

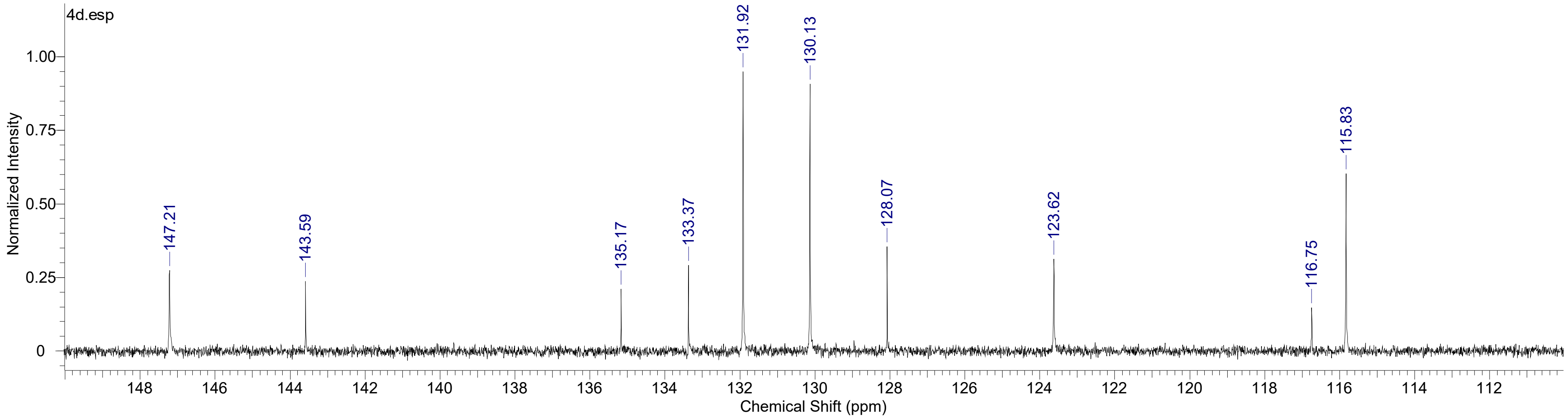

2-(5,6-dibromo-1*H*-benzimidazol-1-yl)-1-(2,4-dichlorophenyl)ethanone (4e)

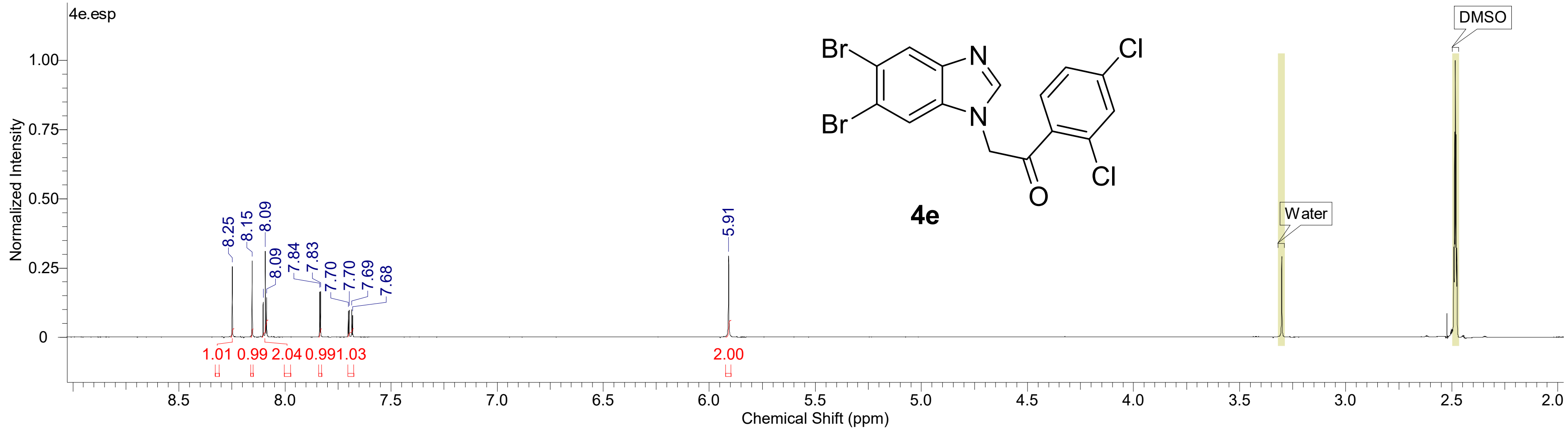

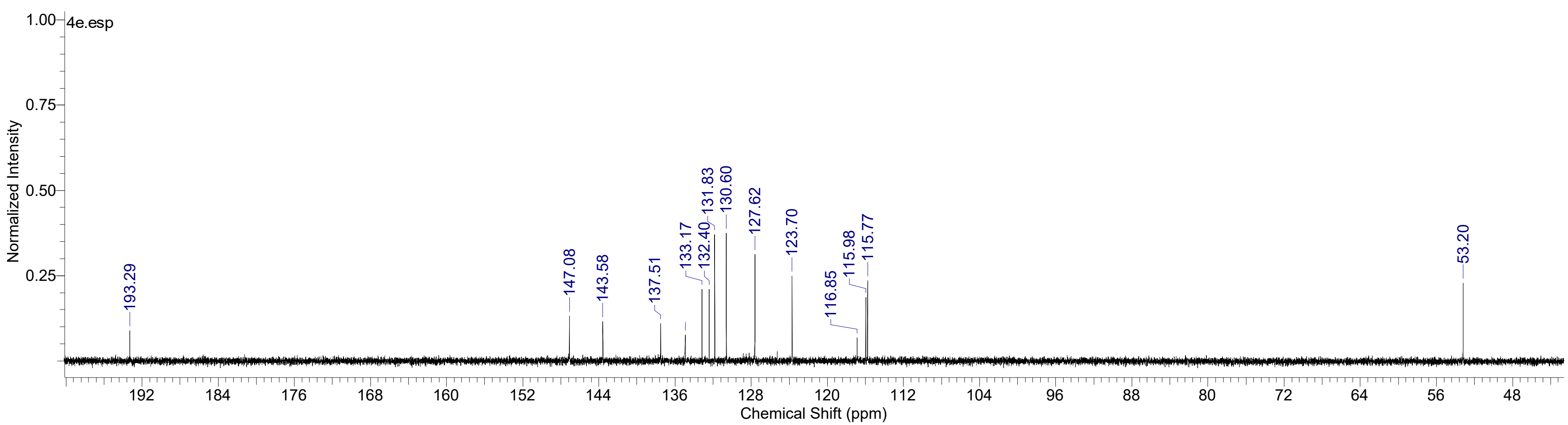
4e.esp
Normalized Intensity
Chemical Shift (ppm)
193.29
147.08
143.58
137.51
133.17
132.40
131.83
130.60
127.62
123.70
116.85
115.98
115.77
53.20

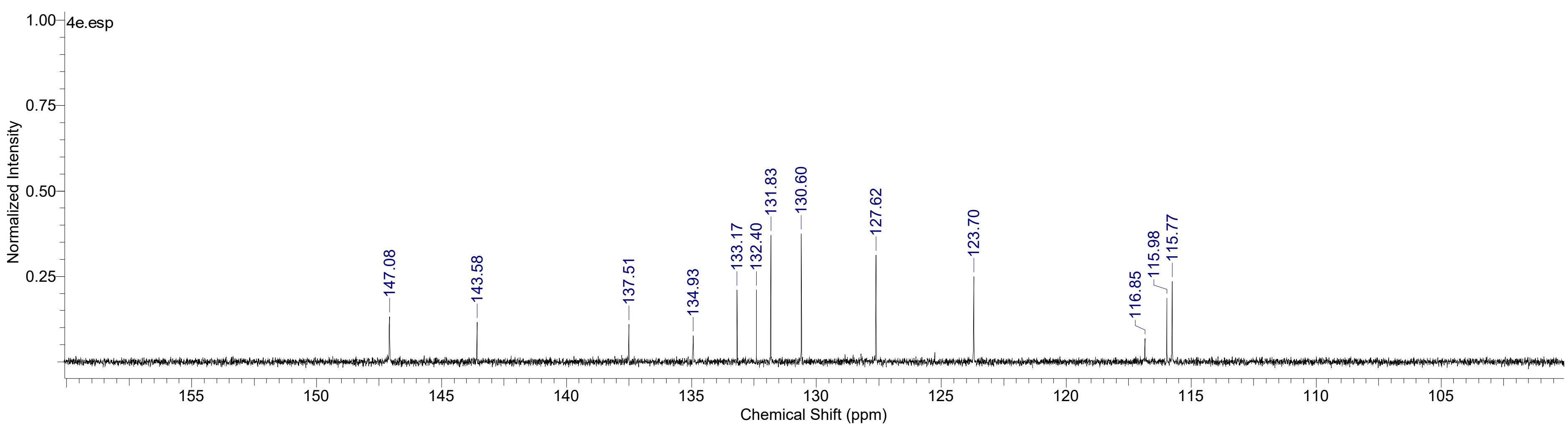
4e.esp
Normalized Intensity
Chemical Shift (ppm)
147.08
143.58
137.51
134.93
133.17
132.40
131.83
130.60
127.62
123.70
116.85
115.98
115.77

2-(5,6-dibromo-1*H*-benzimidazol-1-yl)-1-(3,4-dichlorophenyl)ethanone (4f)

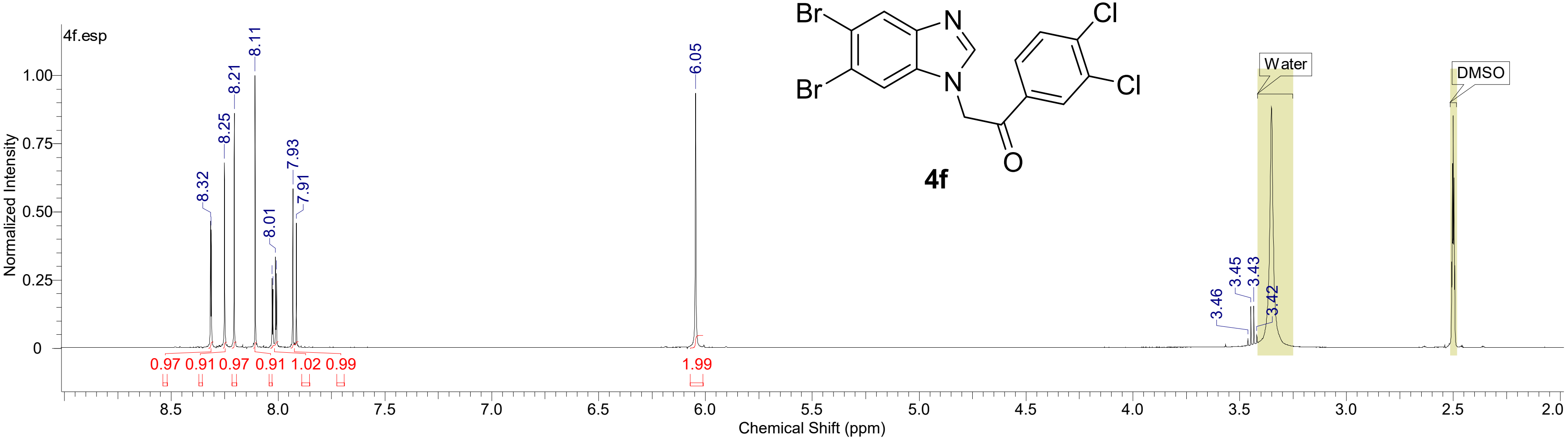

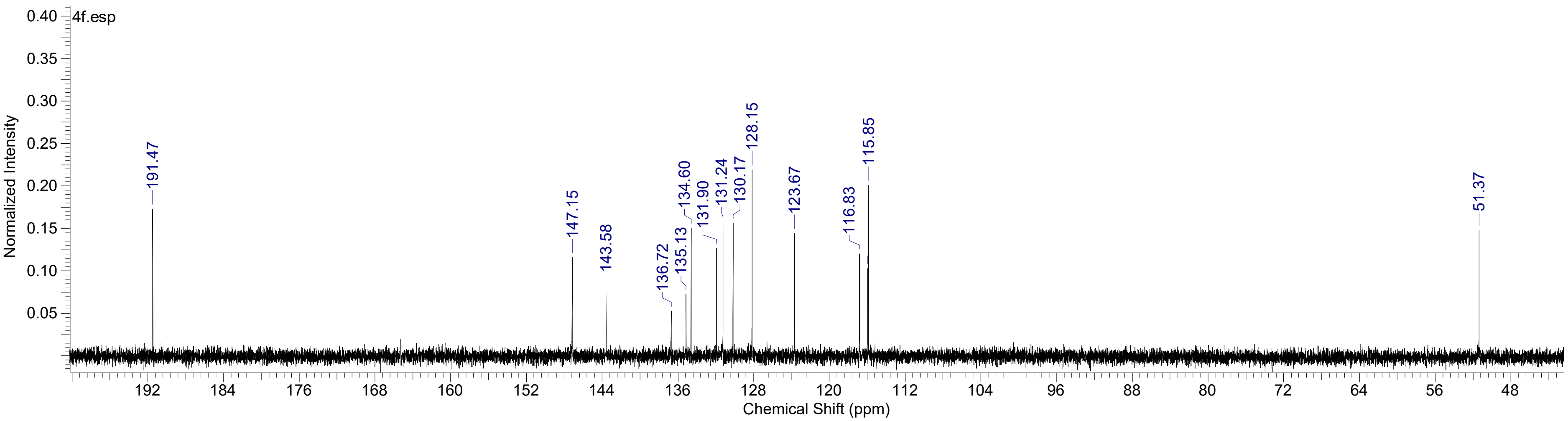

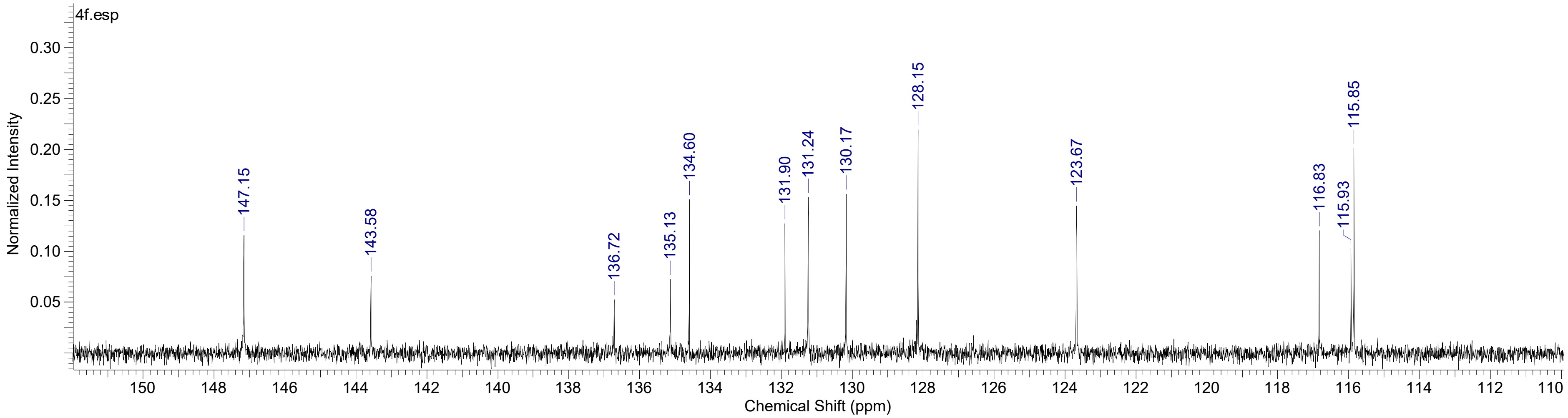

2-(5,6-dibromo-1*H*-benzimidazol-1-yl)-1-(2,4,6-trichlorophenyl)ethanone (4g)

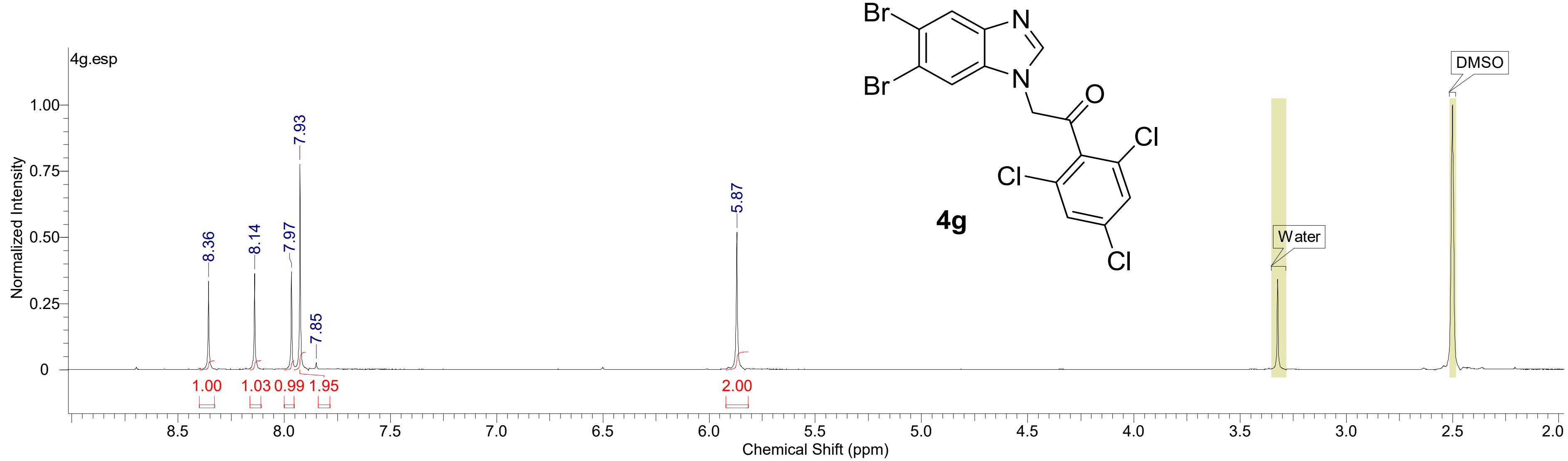

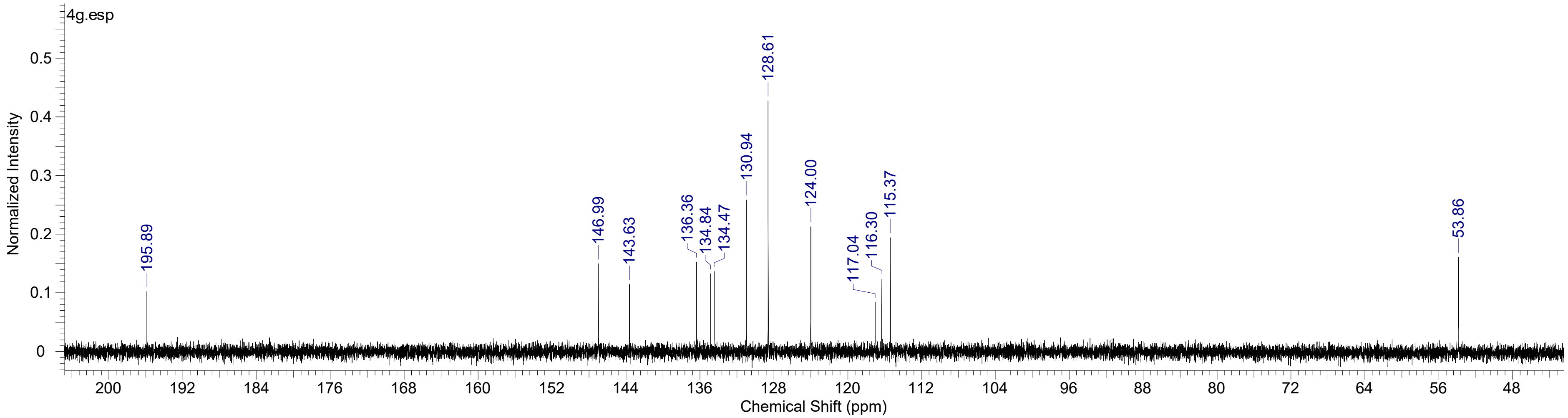
4g.esp
Normalized Intensity
Chemical Shift (ppm)
195.89
146.99
143.63
136.36
134.84
134.47
130.94
128.61
124.00
117.04
116.30
115.37
53.86

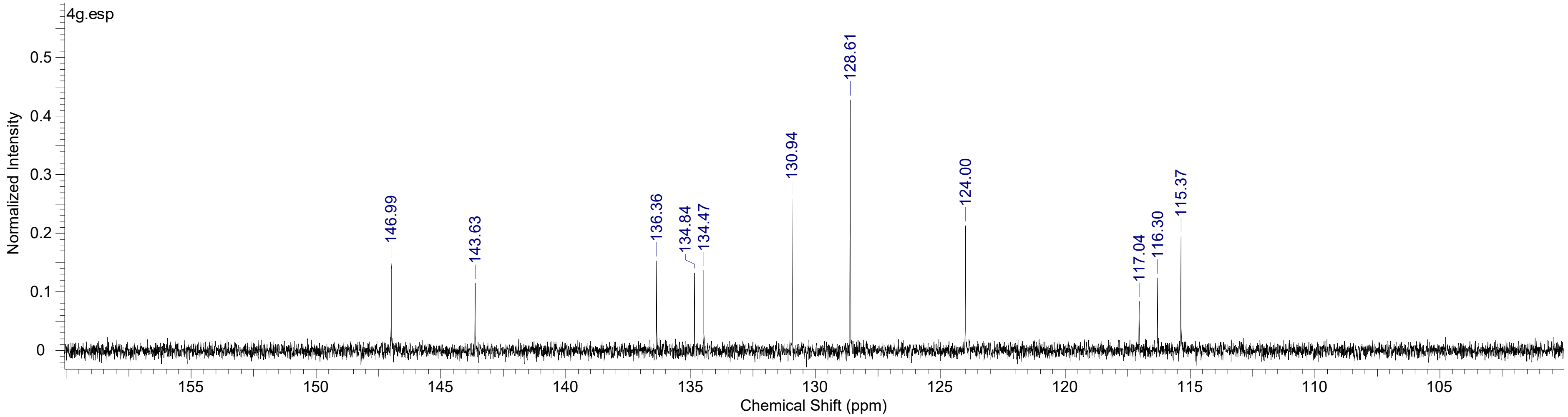
4g.esp
Normalized Intensity
Chemical Shift (ppm)
146.99
143.63
136.36
134.84
134.47
130.94
128.61
124.00
117.04
116.30
115.37

2-(5,6-dibromo-1*H*-benzimidazol-1-yl)-1-(2,4-difluorophenyl)ethanone (4h)

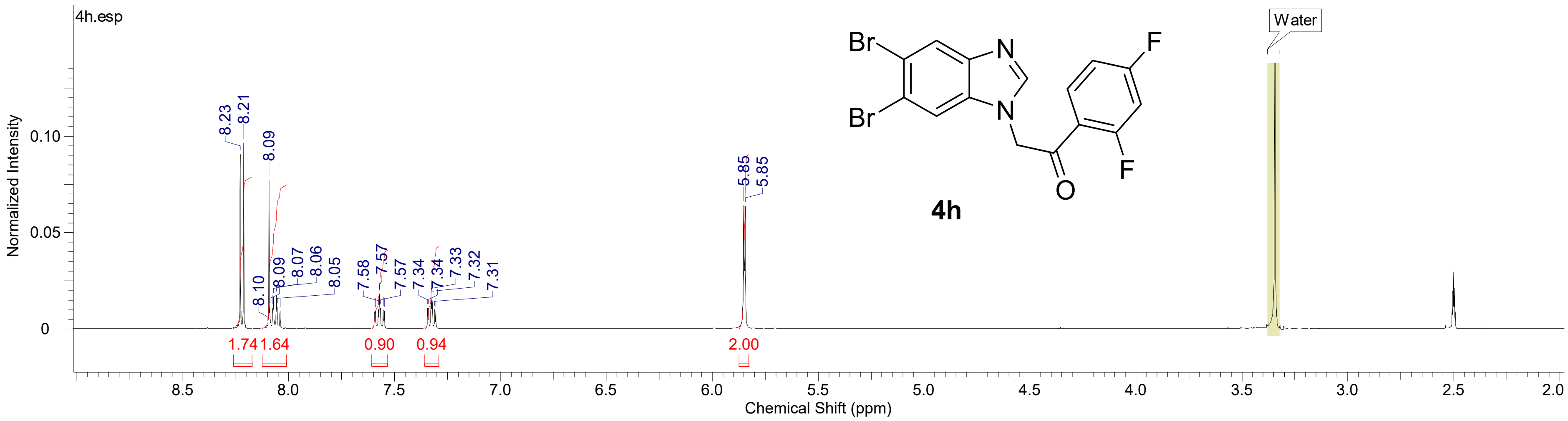

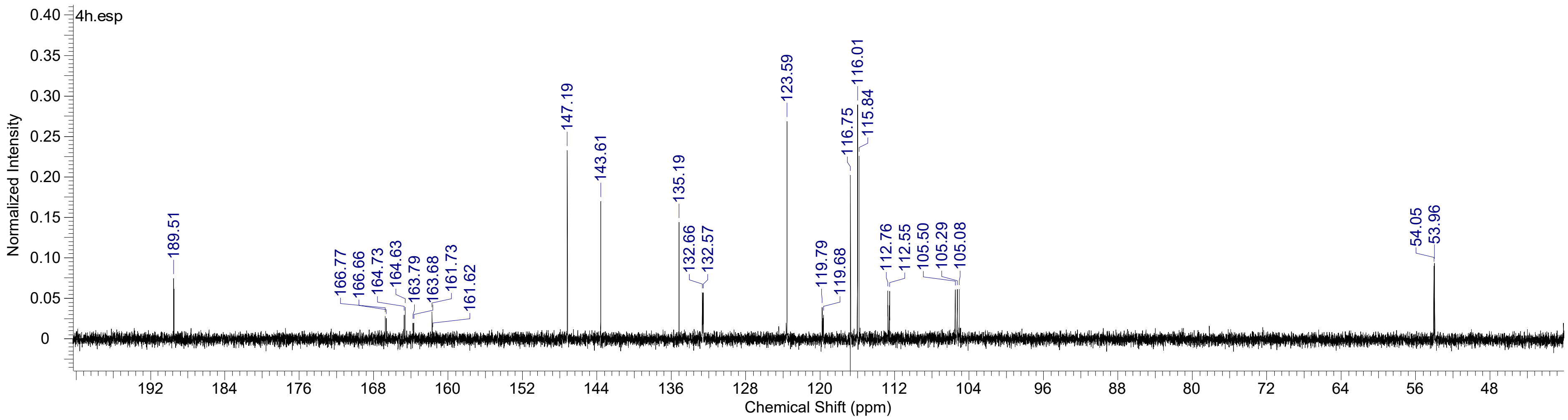

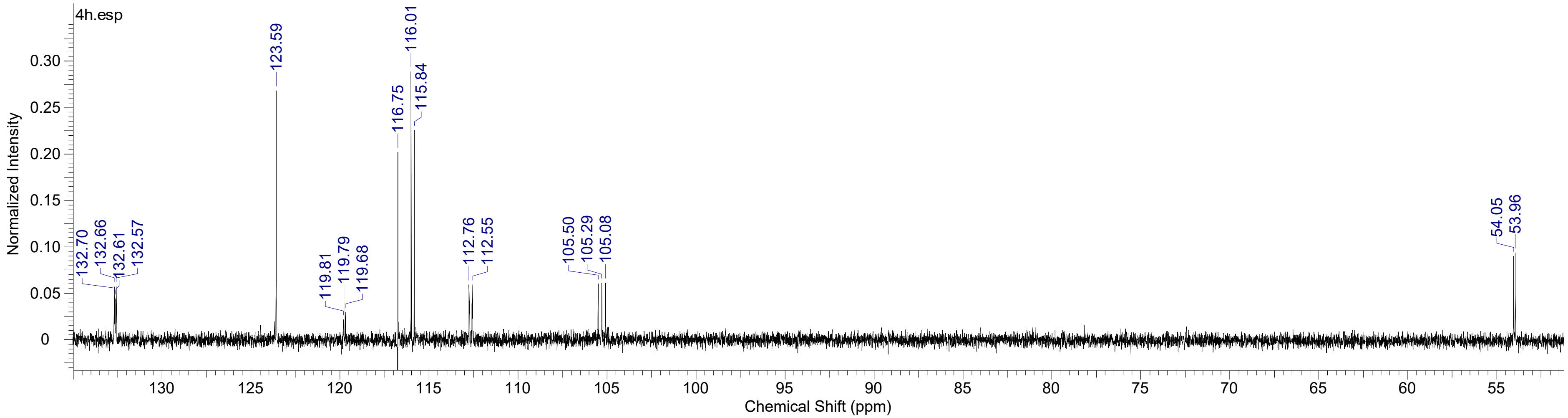
4h.esp
Normalized Intensity
Chemical Shift (ppm)
132.70
132.66
132.61
132.57
123.59
119.81
119.79
119.68
116.75
116.01
115.84
112.76
112.55
105.50
105.29
105.08
54.05
53.96

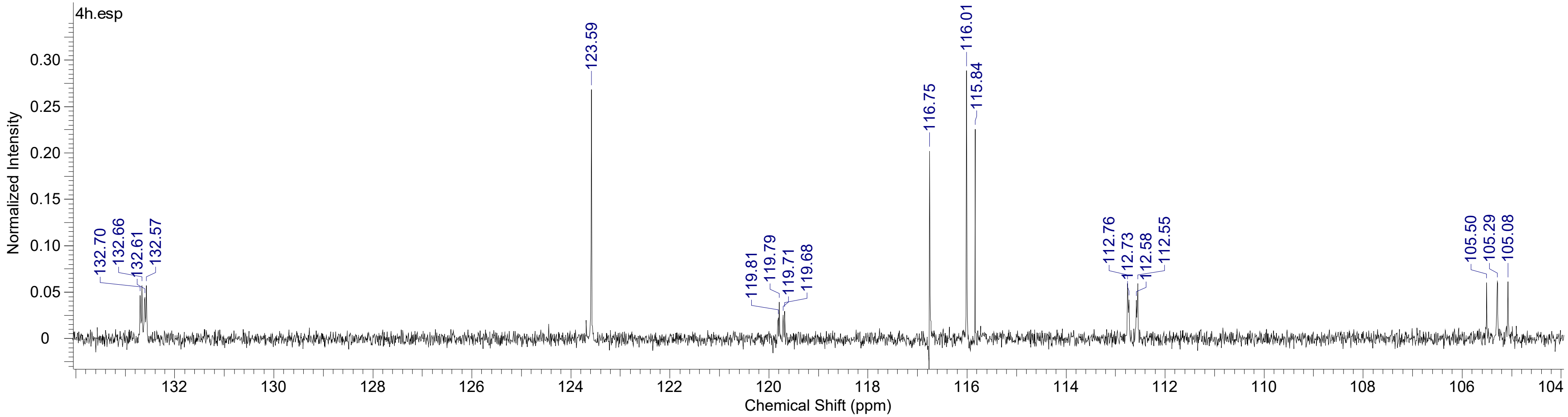
4h.esp
Normalized Intensity
Chemical Shift (ppm)
132.70
132.66
132.61
132.57
123.59
119.81
119.79
119.71
119.68
116.75
116.01
115.84
112.76
112.73
112.58
112.55
105.50
105.29
105.08

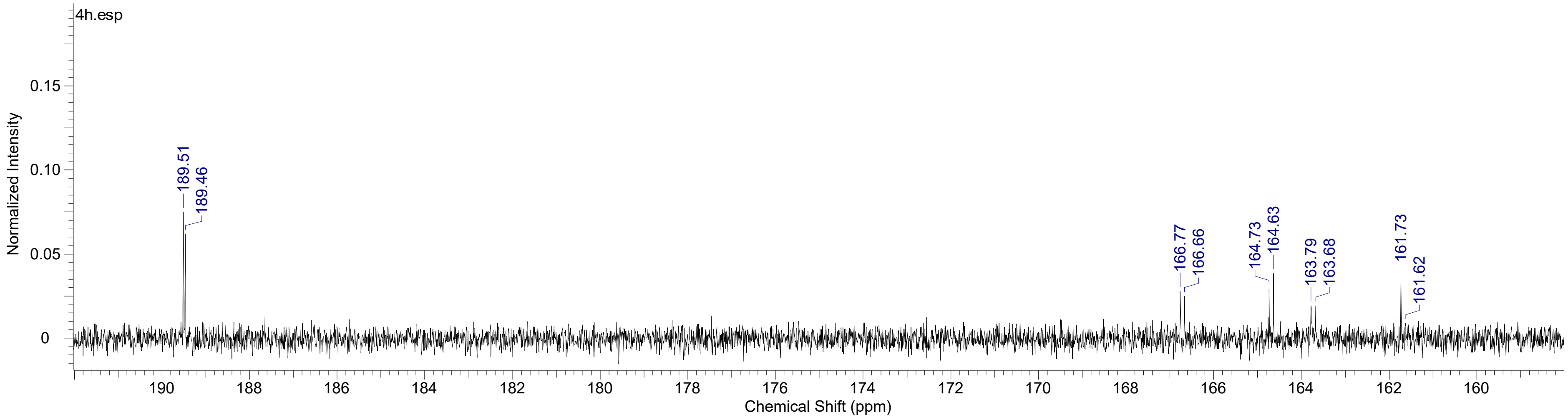

2-(5,6-dibromo-1*H*-benzimidazol-1-yl)-1-(2,5-difluorophenyl)ethanone (4i)

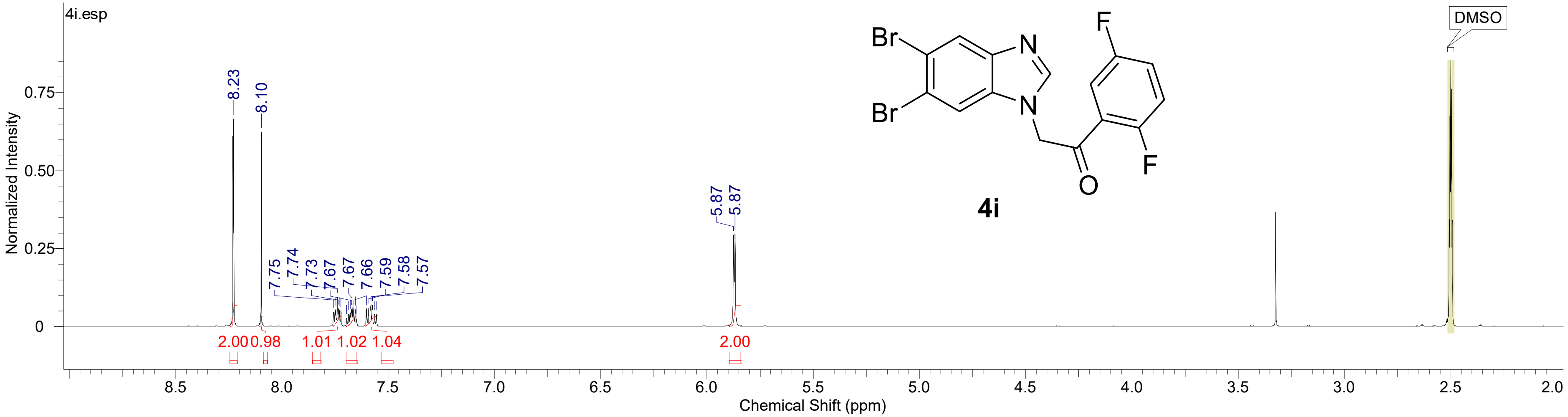

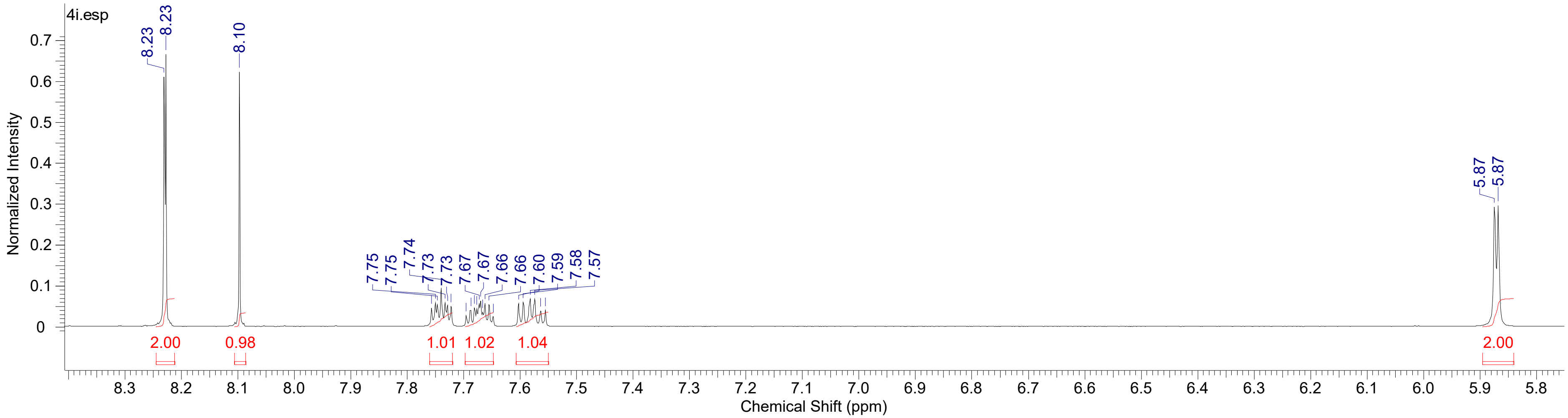
4i.esp
Normalized Intensity
Chemical Shift (ppm)
8.23
8.23
8.10
7.75
7.75
7.74
7.73
7.73
7.67
7.67
7.66
7.66
7.60
7.59
7.58
7.57
5.87
5.87
2.00
0.98
1.01
1.02
1.04
2.00

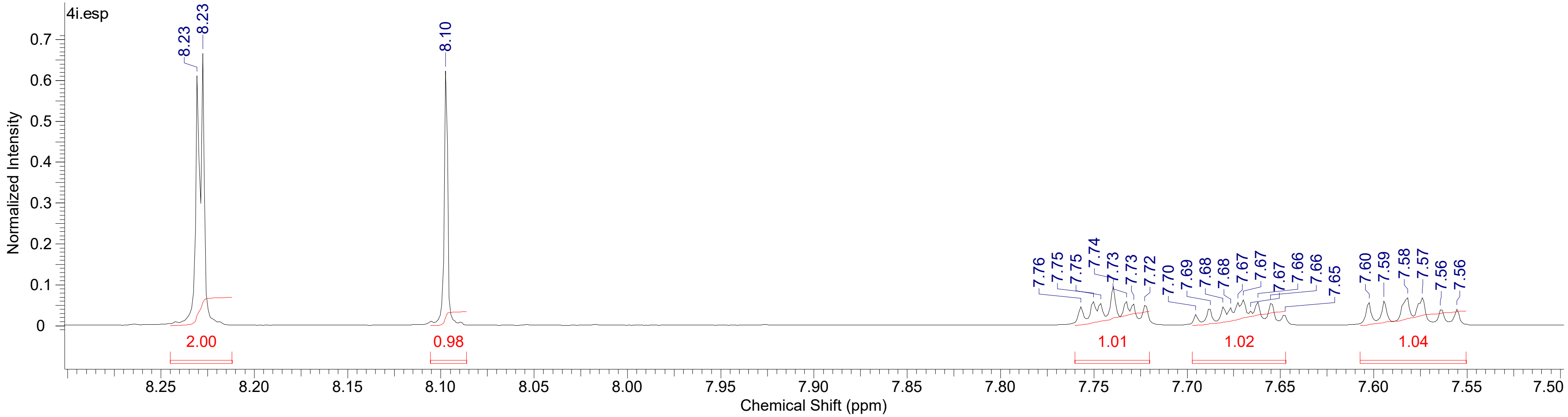
4i.esp
Normalized Intensity
Chemical Shift (ppm)
8.23
8.23
8.10
7.76
7.75
7.75
7.74
7.73
7.73
7.72
7.70
7.69
7.68
7.68
7.67
7.67
7.67
7.66
7.66
7.65
7.60
7.59
7.58
7.57
7.56
7.56
2.00
0.98
1.01
1.02
1.04

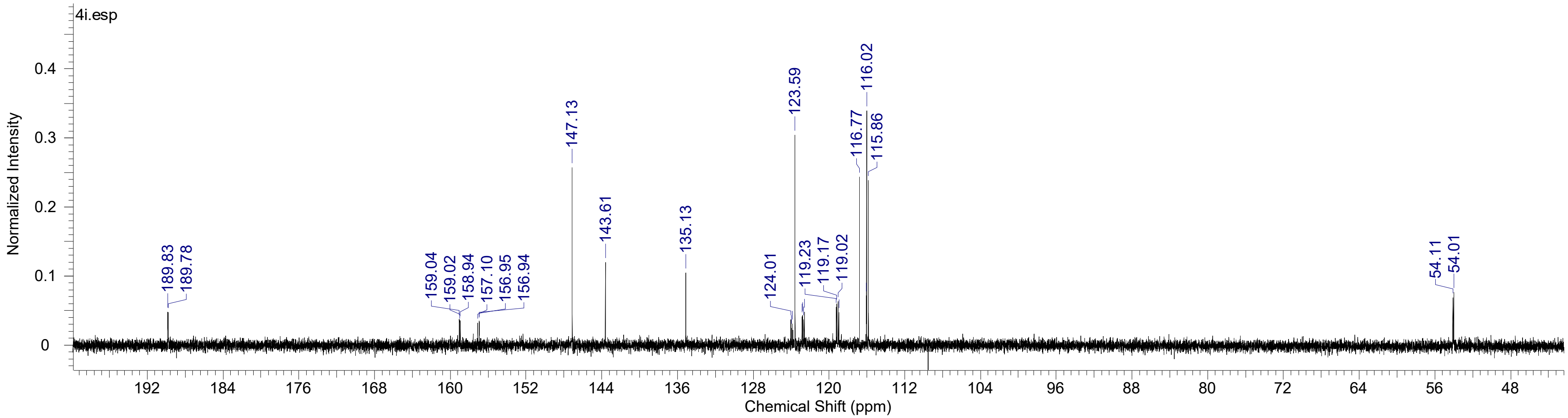
4i.esp
Normalized Intensity
Chemical Shift (ppm)
189.83
189.78
159.04
159.02
158.94
157.10
156.95
156.94
147.13
143.61
135.13
124.01
123.59
119.23
119.17
119.02
116.77
116.02
115.86
54.11
54.01

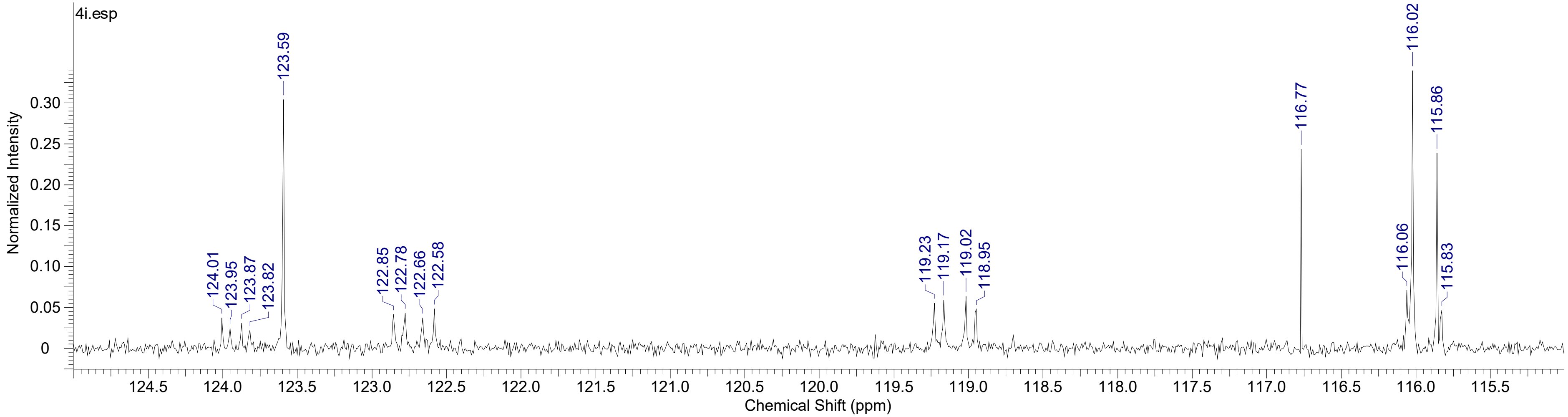
4i.esp
Normalized Intensity
Chemical Shift (ppm)
124.01
123.95
123.87
123.82
123.59
122.85
122.78
122.66
122.58
119.23
119.17
119.02
118.95
116.77
116.06
116.02
115.86
115.83

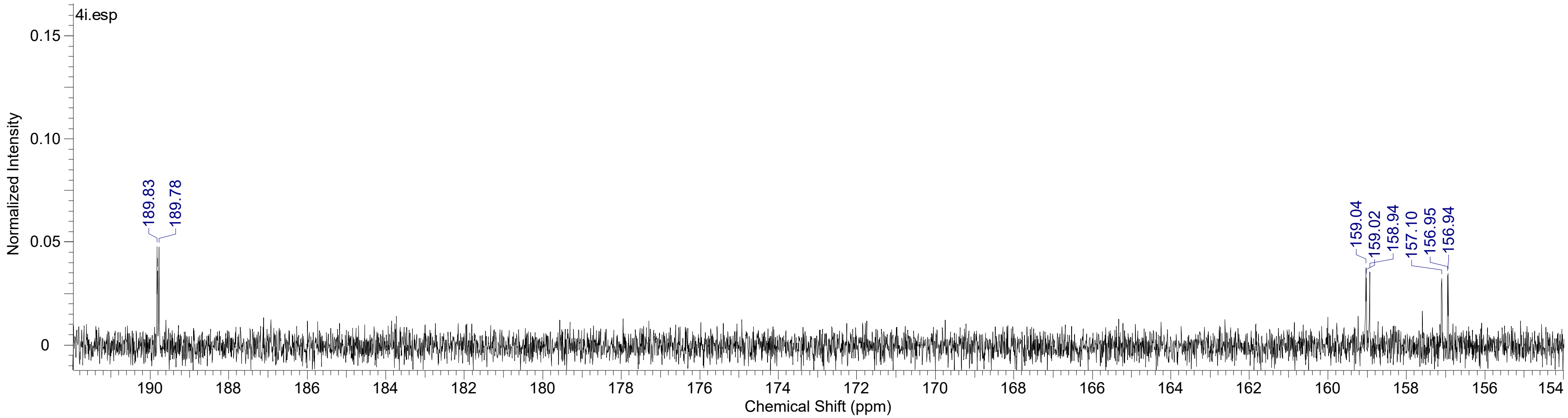

2-(4,6-dibromo-1*H*-benzimidazol-1-yl)-1-phenylethanone (5a)

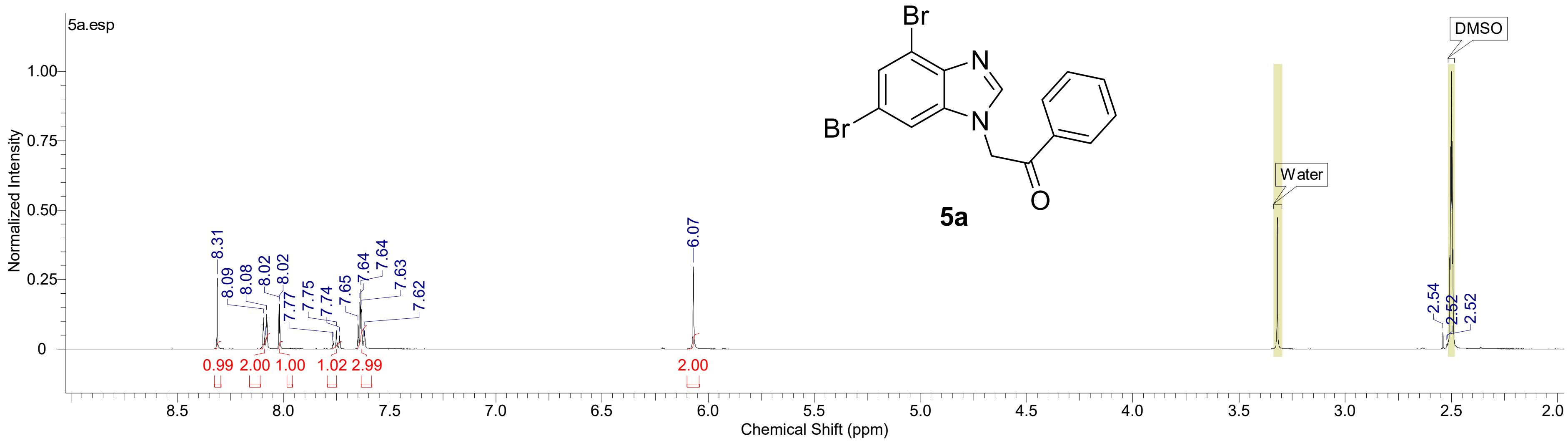

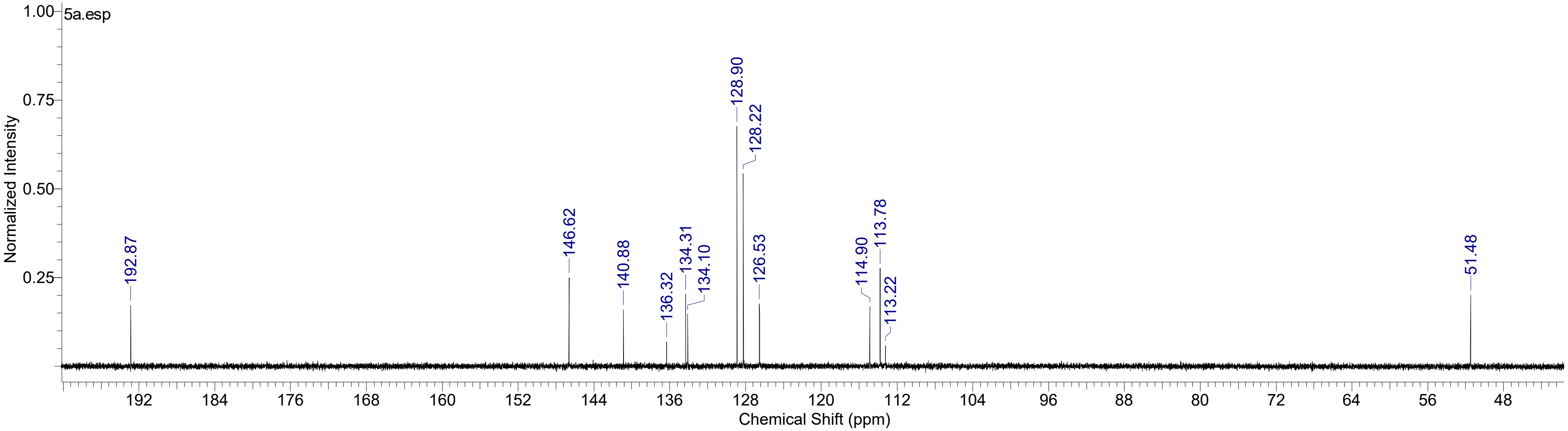

2-(4,6-dibromo-1*H*-benzimidazol-1-yl)-1-(4-chlorophenyl)ethanone (5c)

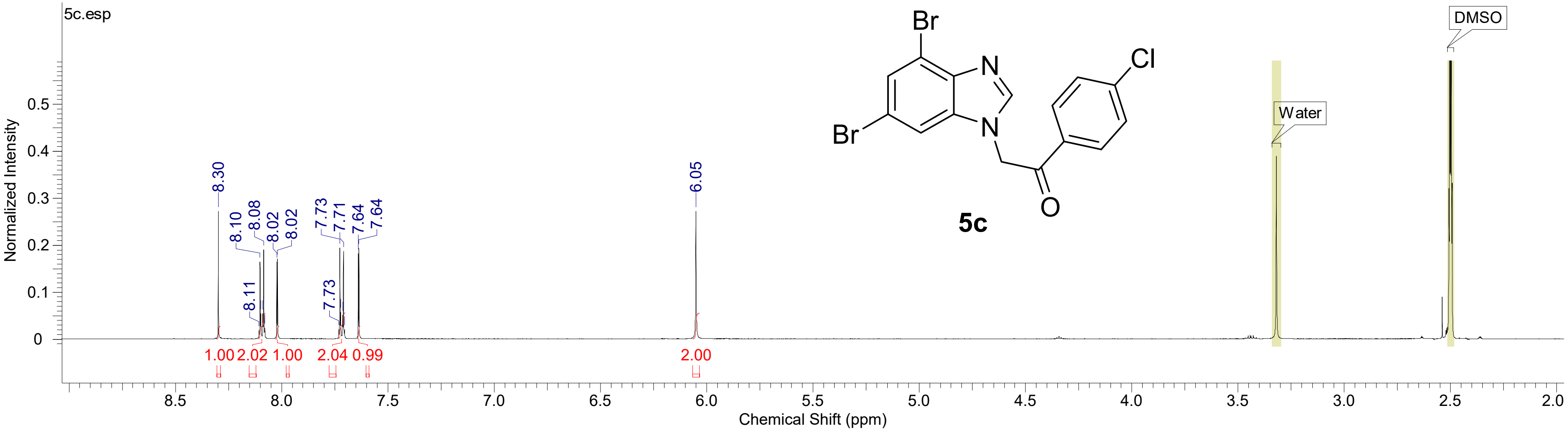

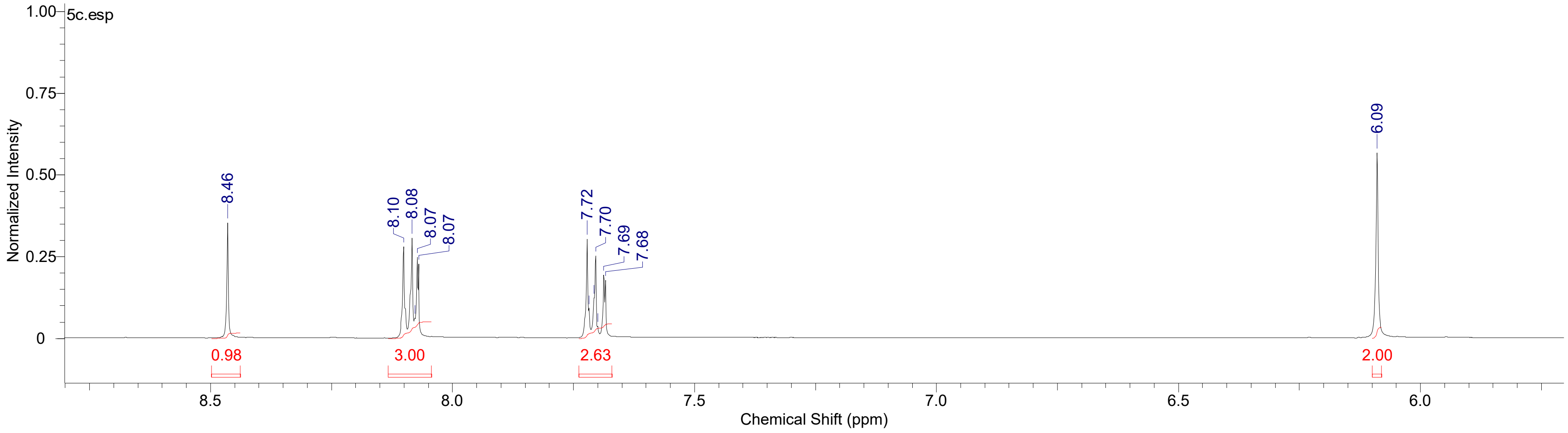
5c.esp
8.46
8.10
8.08
8.07
8.07
7.72
7.70
7.69
7.68
6.09
0.98
3.00
2.63
2.00
Normalized Intensity
Chemical Shift (ppm)

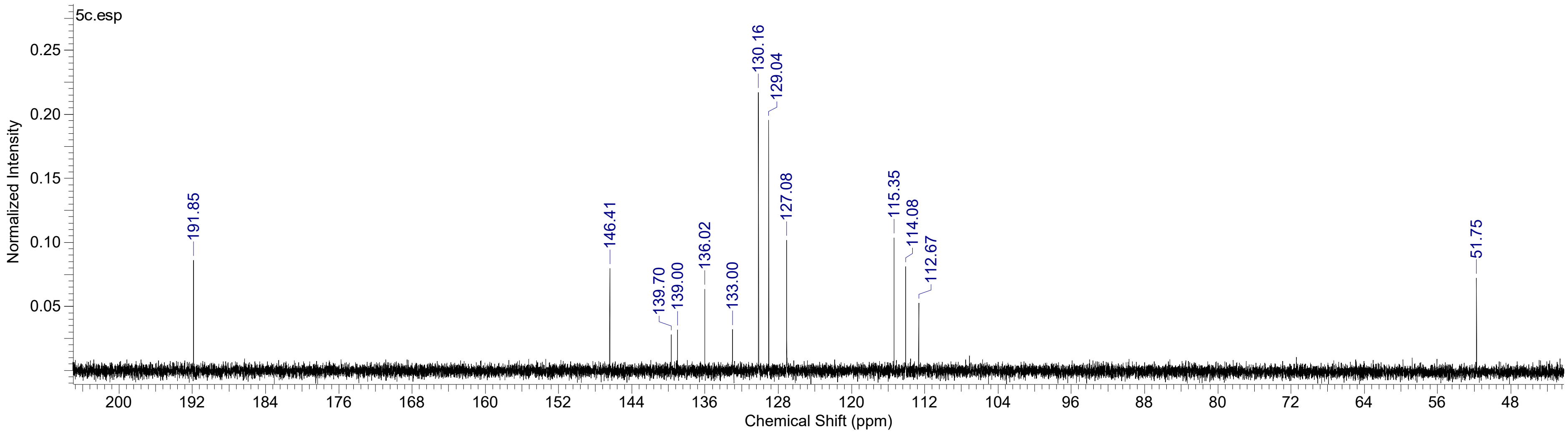
5c.esp
191.85
146.41
139.70
139.00
136.02
133.00
130.16
129.04
127.08
115.35
114.08
112.67
51.75
Normalized Intensity
Chemical Shift (ppm)

2-(4,6-dibromo-1*H*-benzimidazol-1-yl)-1-(2,4,6-trichlorophenyl)ethanone (5g)

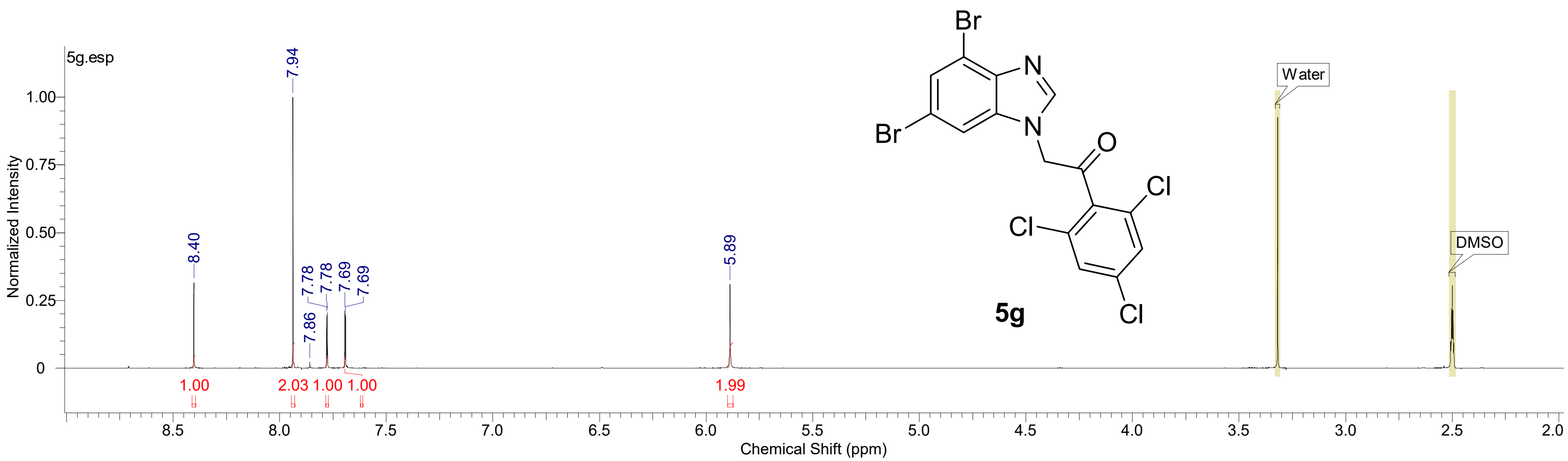

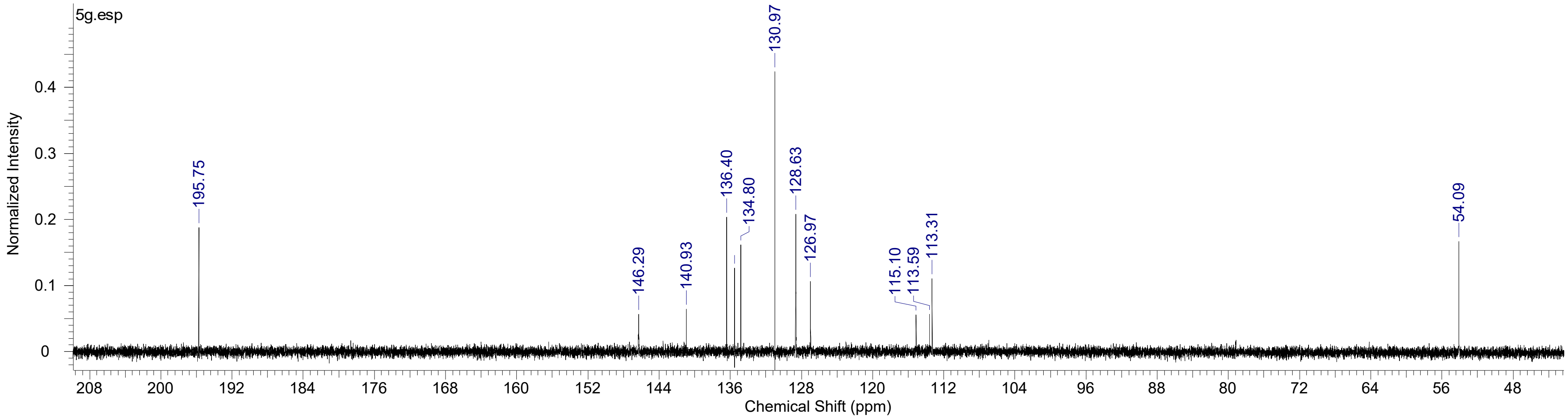
5g.esp
Normalized Intensity
Chemical Shift (ppm)
195.75
146.29
140.93
136.40
134.80
130.97
128.63
126.97
115.10
113.59
113.31
54.09

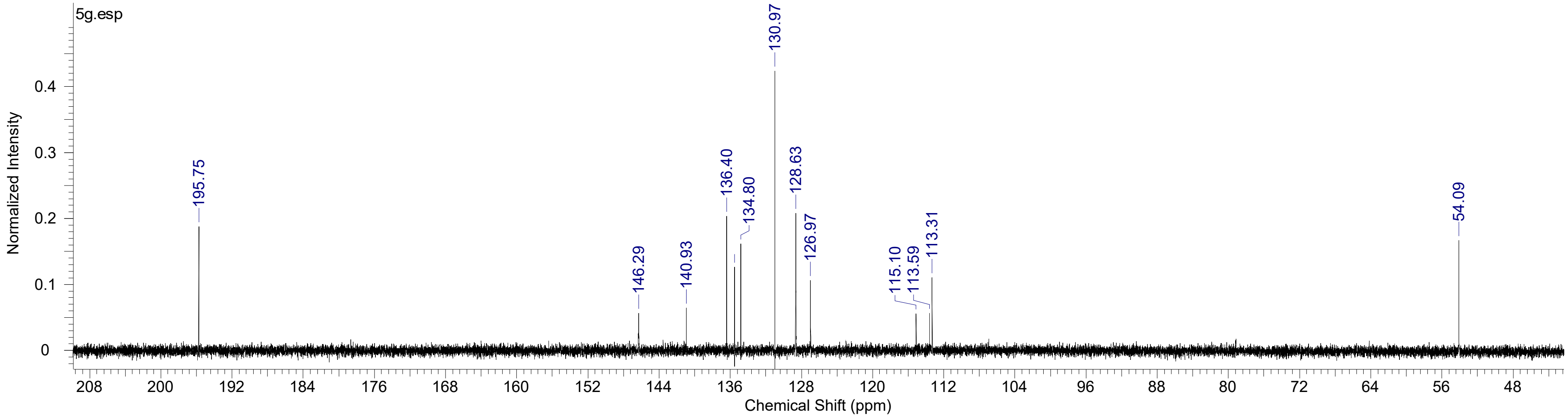
5g.esp
Normalized Intensity
Chemical Shift (ppm)
195.75
146.29
140.93
136.40
134.80
130.97
128.63
126.97
115.10
113.59
113.31
54.09

2-(4,6-dibromo-1*H*-benzimidazol-1-yl)-1-(2,5-difluorophenyl)ethanone (5i)

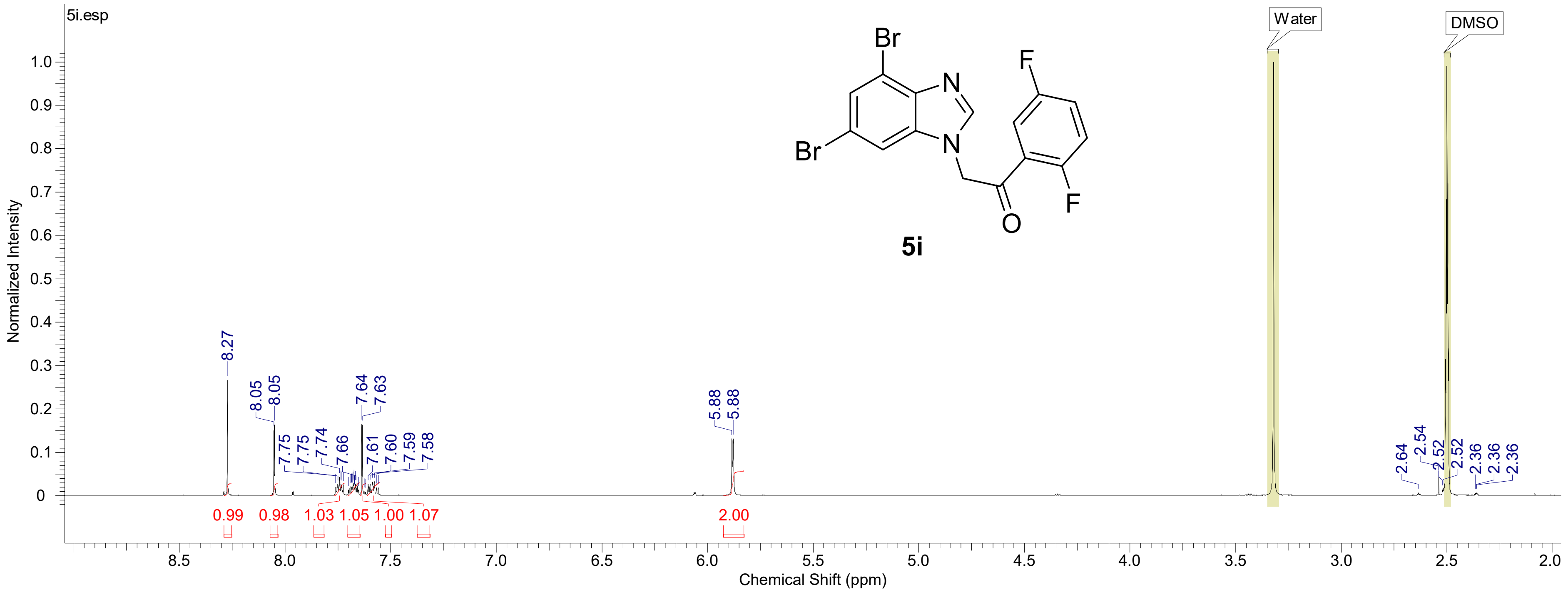

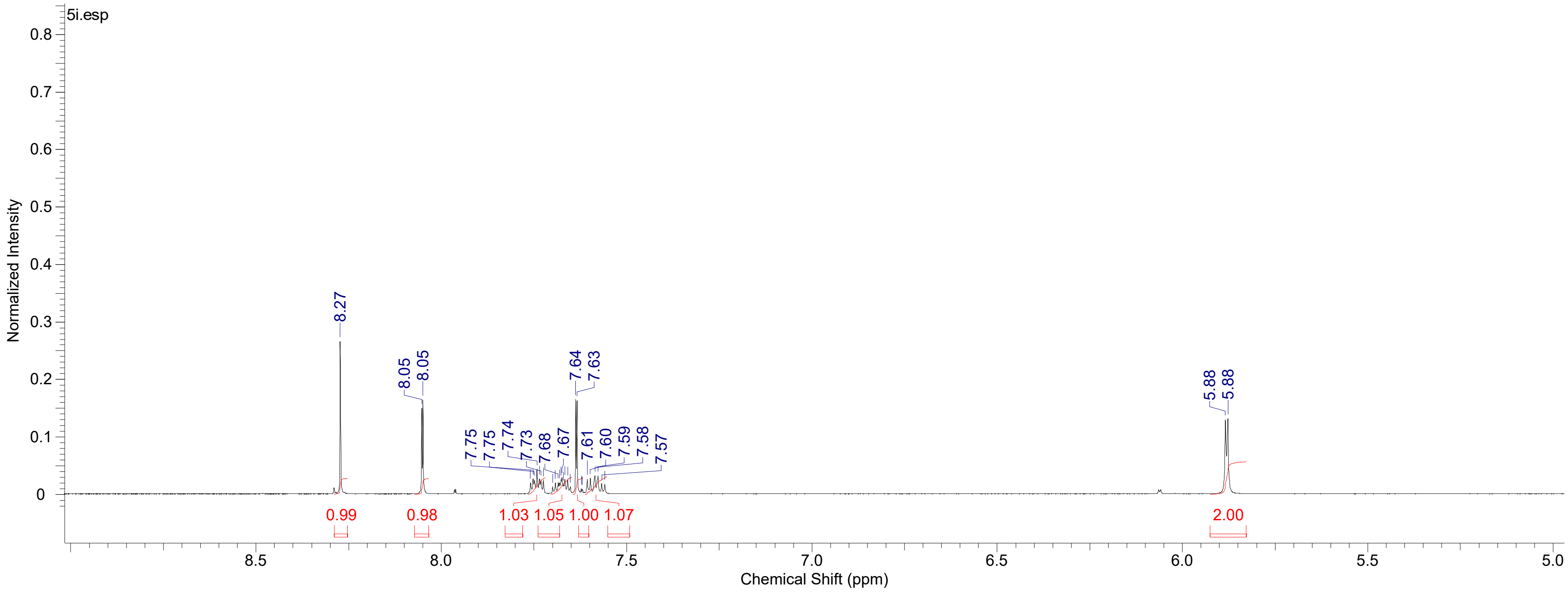
5i.esp
Normalized Intensity
Chemical Shift (ppm)
8.27
8.05
8.05
7.75
7.75
7.74
7.73
7.68
7.67
7.64
7.63
7.61
7.60
7.59
7.58
7.57
5.88
5.88
0.99
0.98
1.03
1.05
1.00
1.07
2.00

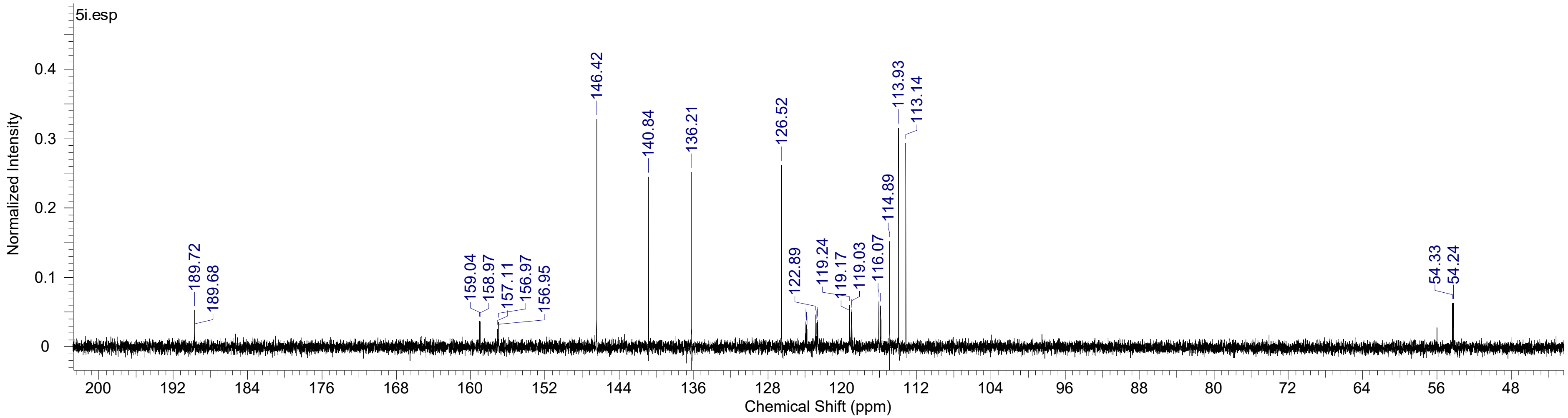
5i.esp
Normalized Intensity
Chemical Shift (ppm)
189.72
189.68
159.04
158.97
157.11
156.97
156.95
146.42
140.84
136.21
126.52
122.89
119.24
119.17
119.03
116.07
114.89
113.93
113.14
54.33
54.24

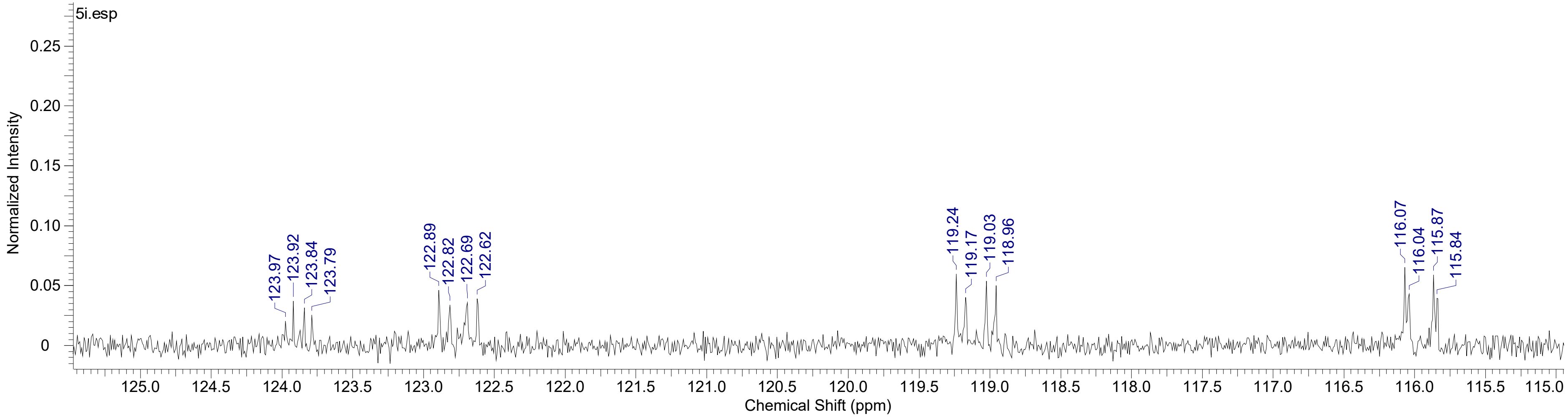
5i.esp
Normalized Intensity
Chemical Shift (ppm)
123.97
123.92
123.84
123.79
122.89
122.82
122.69
122.62
119.24
119.17
119.03
118.96
116.07
116.04
115.87
115.84

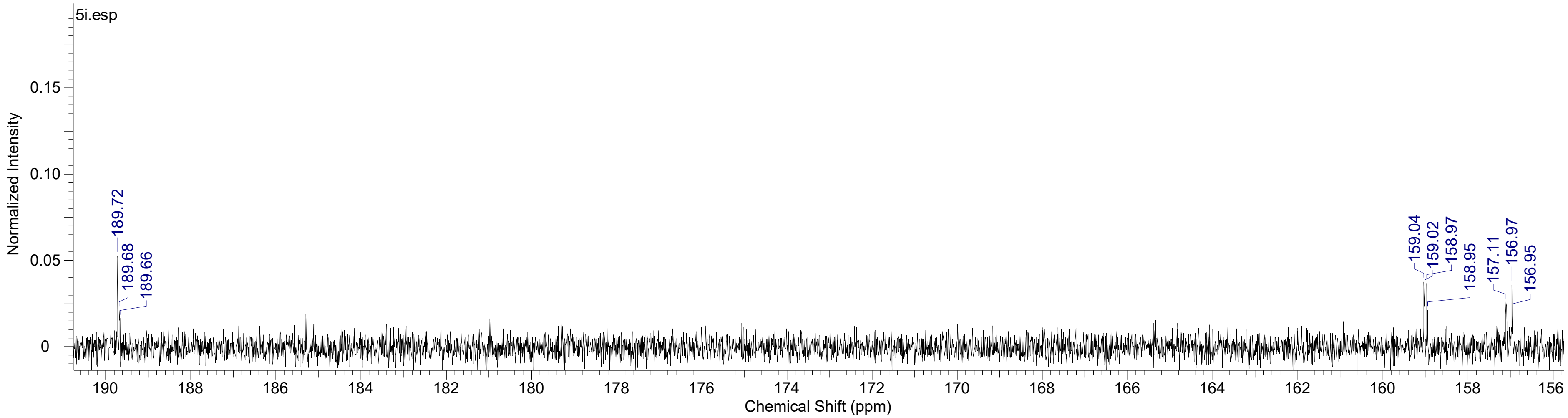
5i.esp
Normalized Intensity
0.15
0.10
0.05
0
189.72
189.68
189.66
159.04
159.02
158.97
158.95
157.11
156.97
156.95
190
188
186
184
182
180
178
176
174
172
170
168
166
164
162
160
158
156
Chemical Shift (ppm)

4. HRMS of compounds 4 and 5

2-(5,6-dibromo-1*H*-benzimidazol-1-yl)-1-(4-chlorophenyl)ethanone (4c)

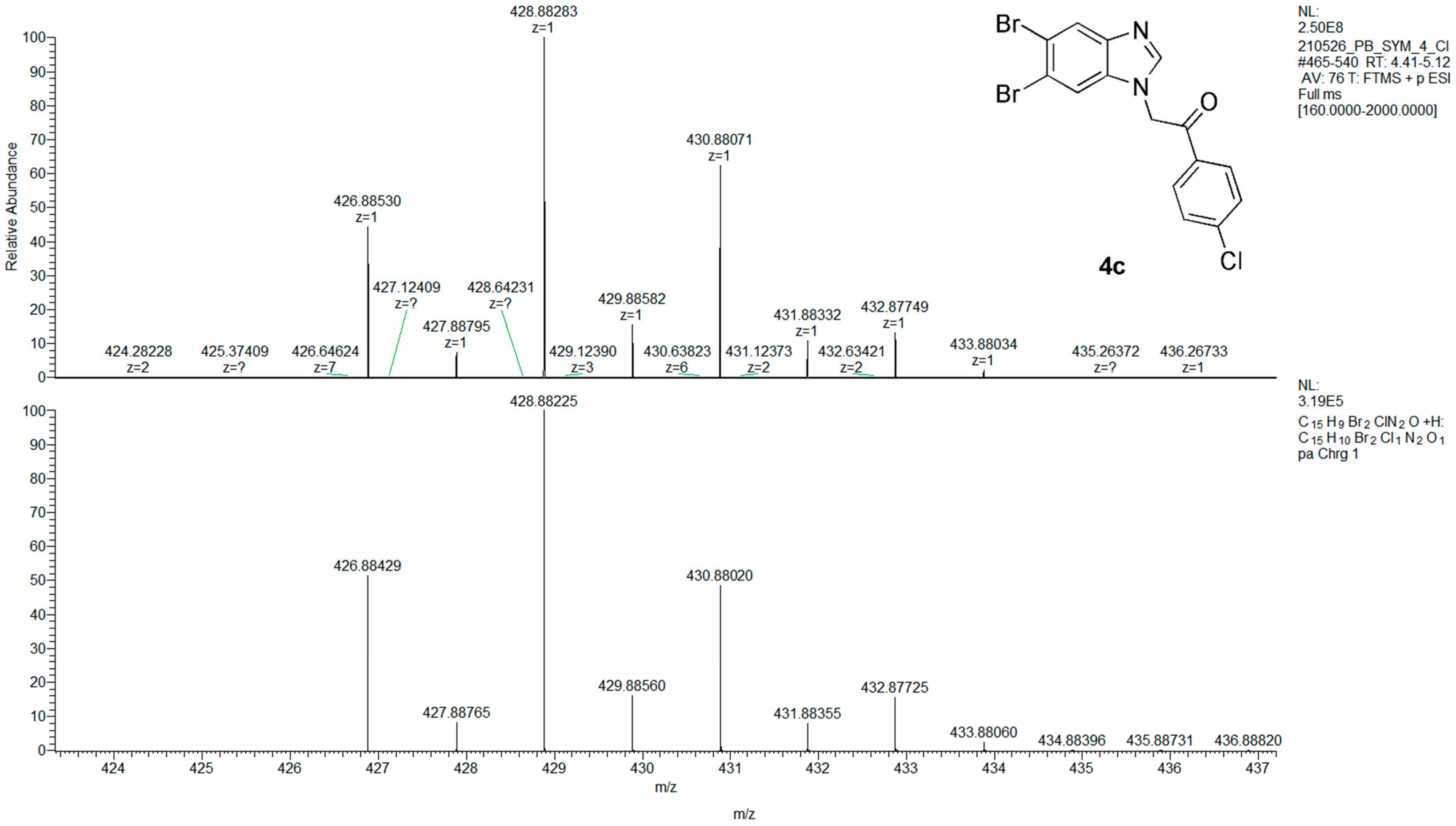

2-(5,6-dibromo-1*H*-benzimidazol-1-yl)-1-(4-bromophenyl)ethanone (4d)

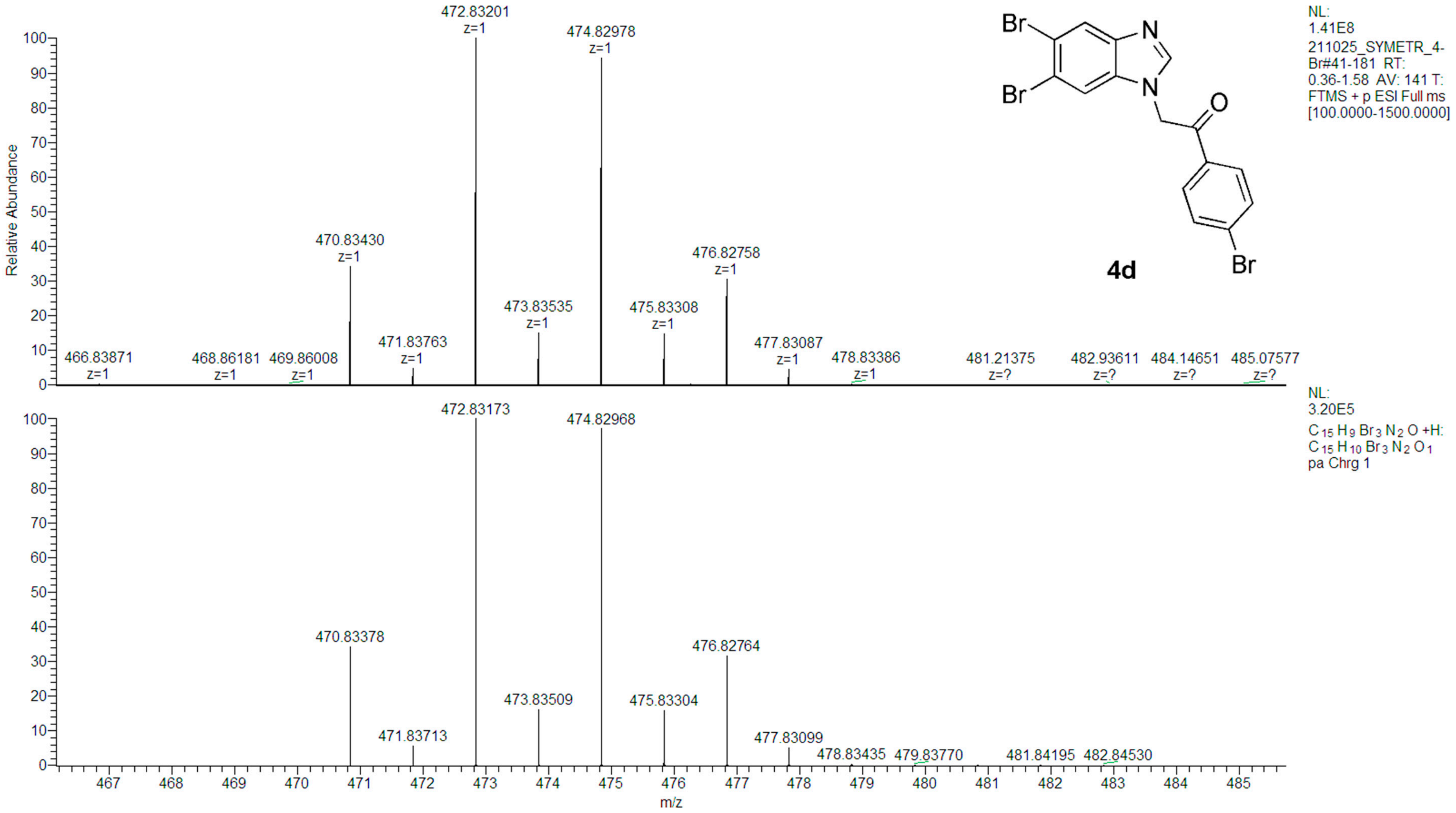

2-(5,6-dibromo-1*H*-benzimidazol-1-yl)-1-(2,4-dichlorophenyl)ethanone (4e)

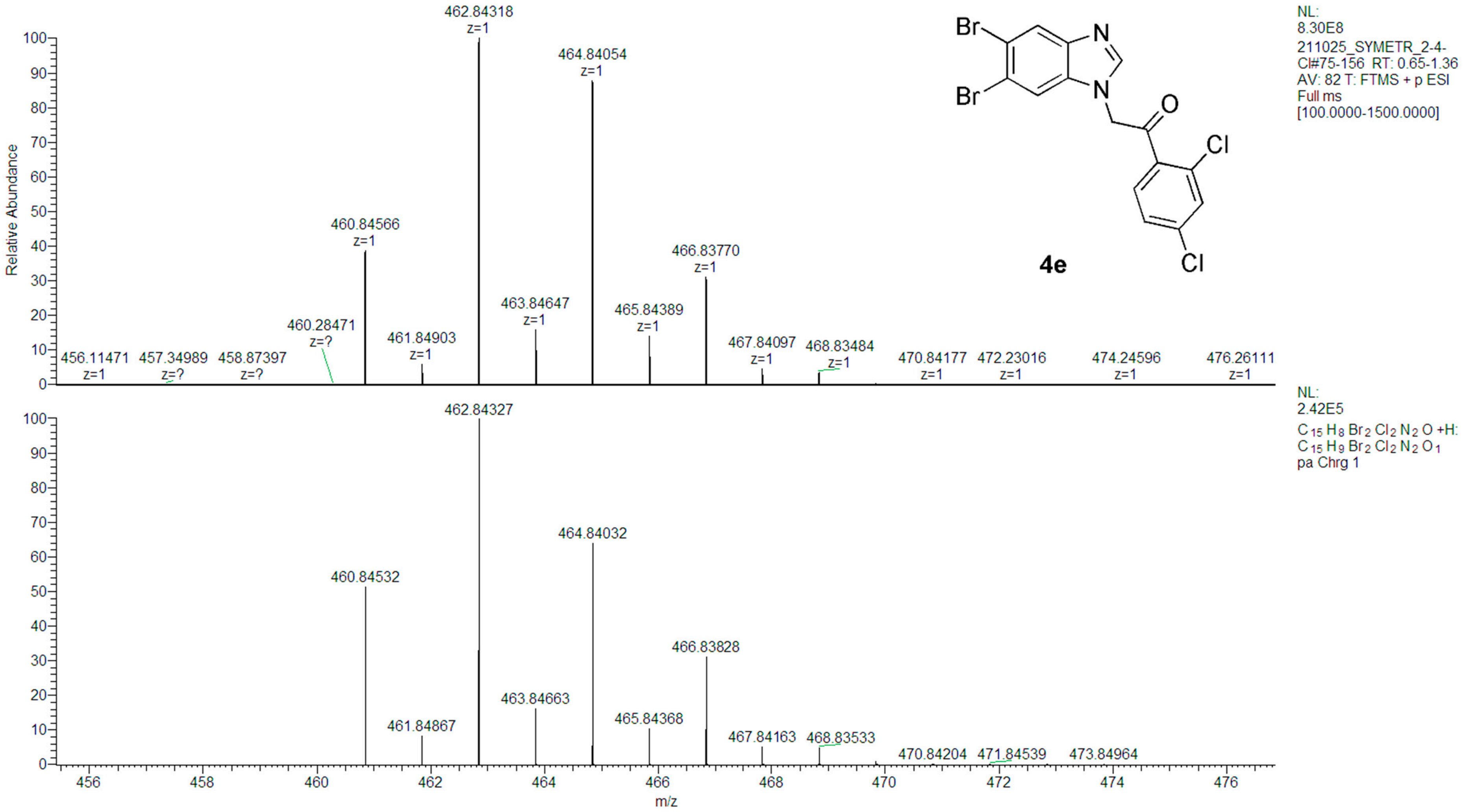

2-(5,6-dibromo-1*H*-benzimidazol-1-yl)-1-(3,4-dichlorophenyl)ethanone (4f)

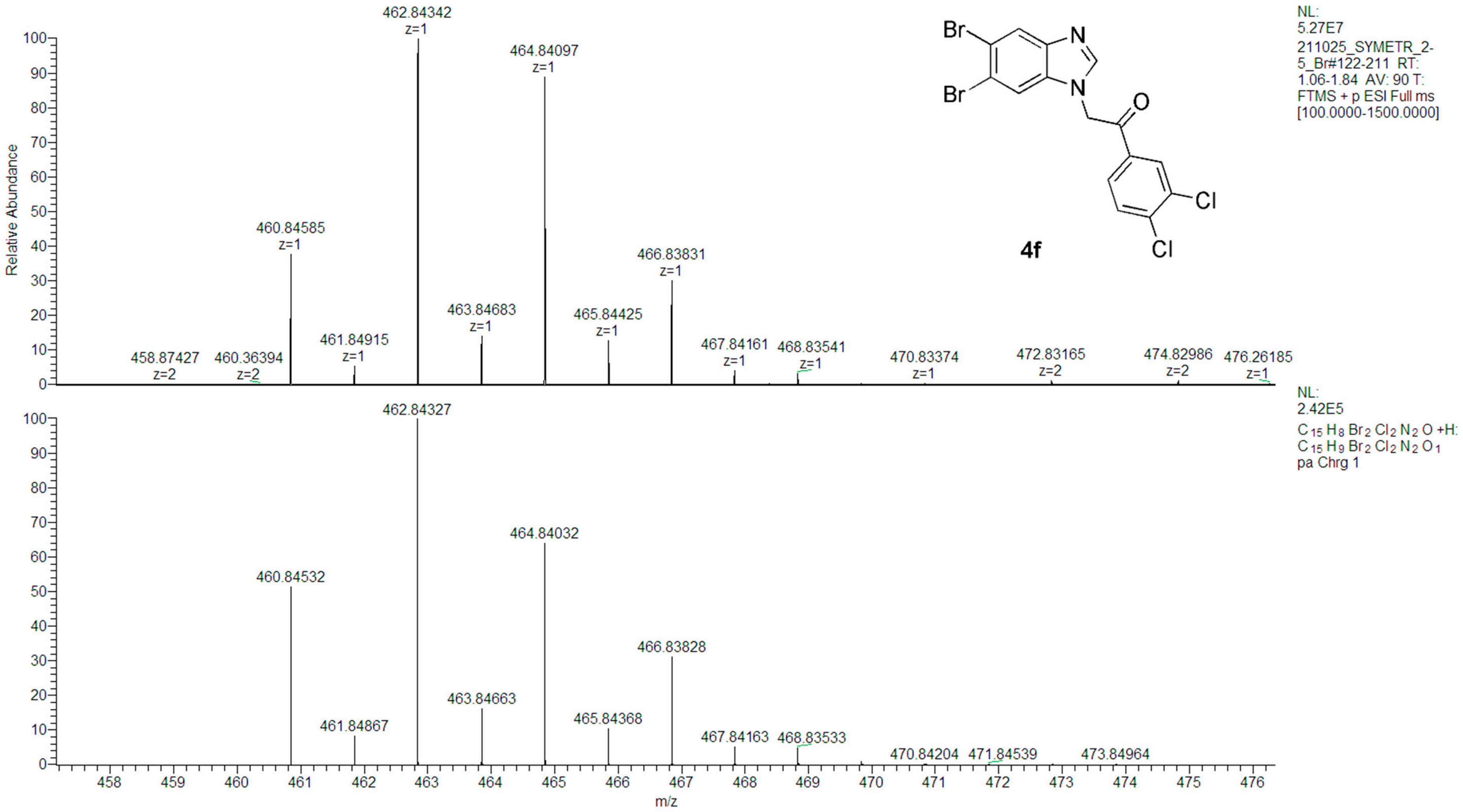

2-(5,6-dibromo-1*H*-benzimidazol-1-yl)-1-(2,4,6-trichlorophenyl)ethanone (**4g**)

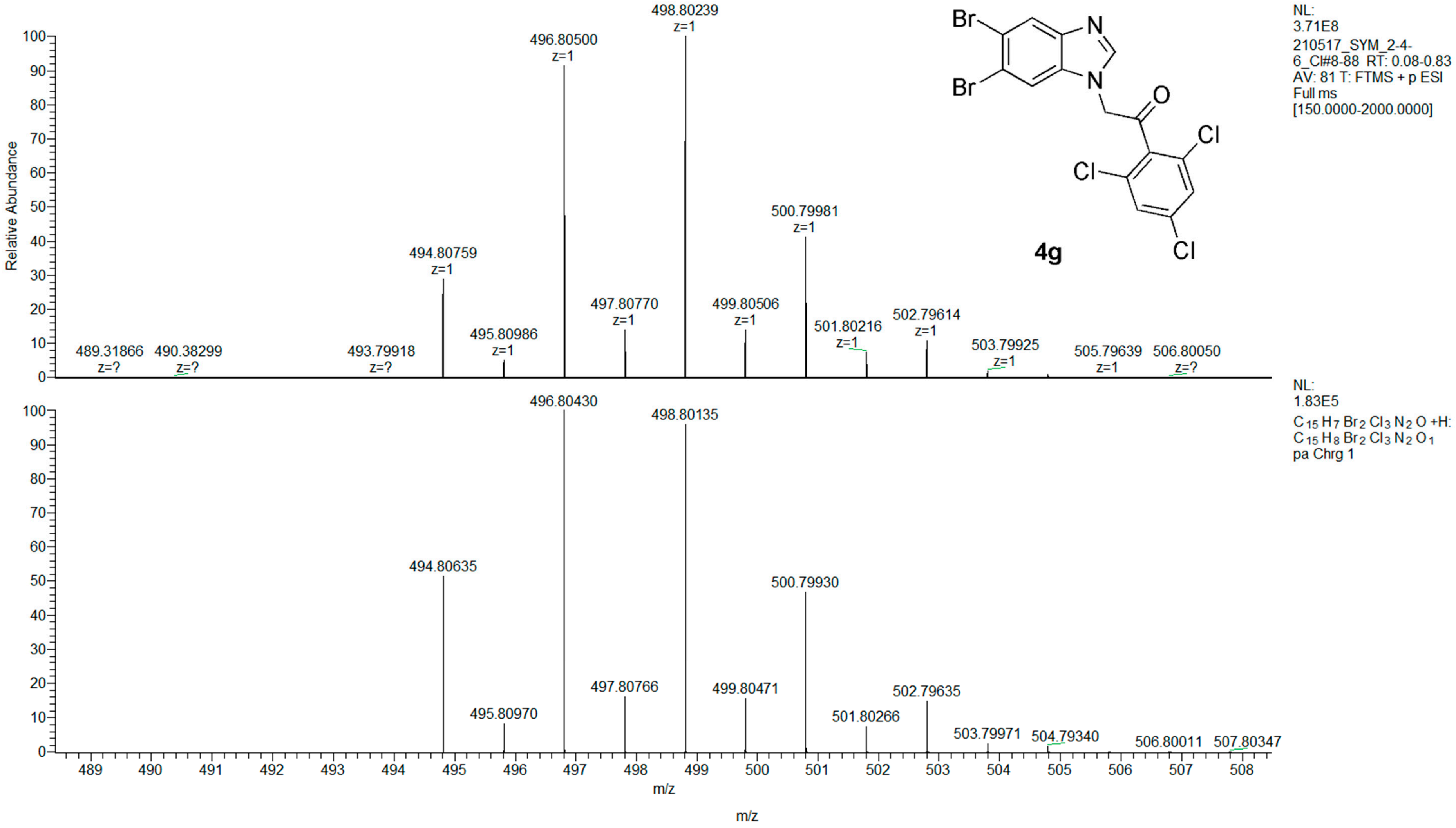

2-(5,6-dibromo-1*H*-benzimidazol-1-yl)-1-(2,4-difluorophenyl)ethanone (4h)

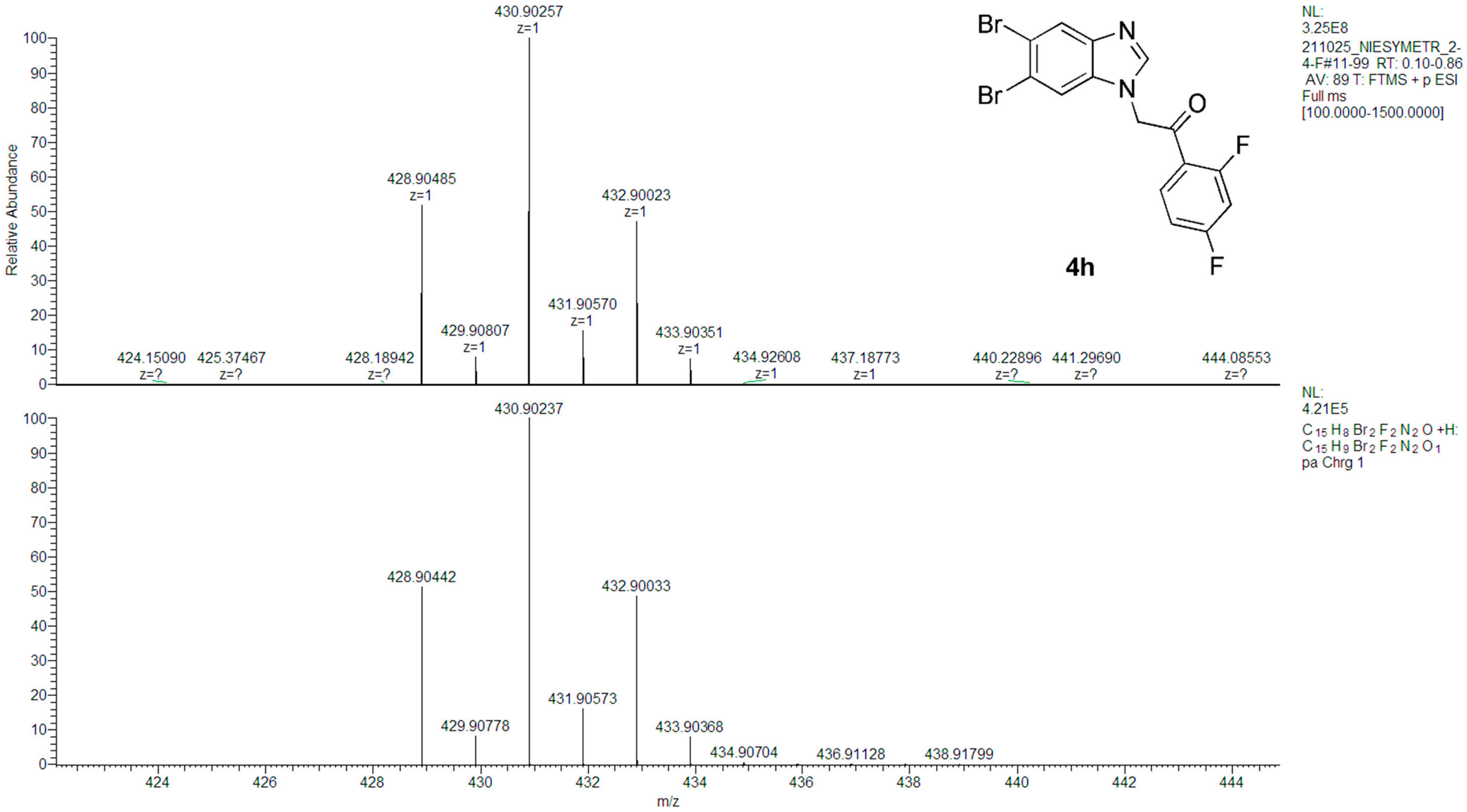

2-(5,6-dibromo-1*H*-benzimidazol-1-yl)-1-(2,5-difluorophenyl)ethanone (4i)

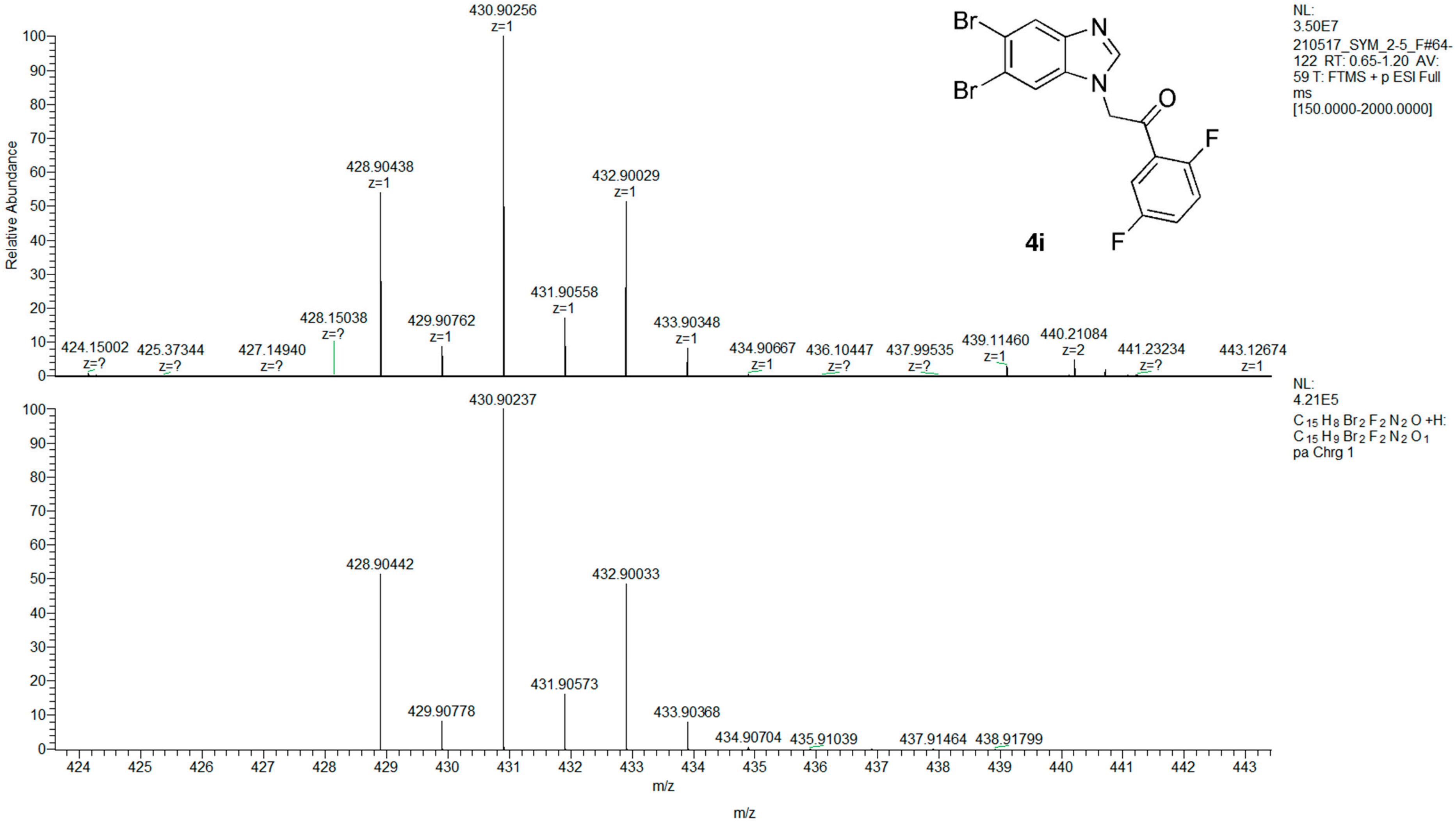

2-(4,6-dibromo-1*H*-benzimidazol-1-yl)-1-phenylethanone (5a)

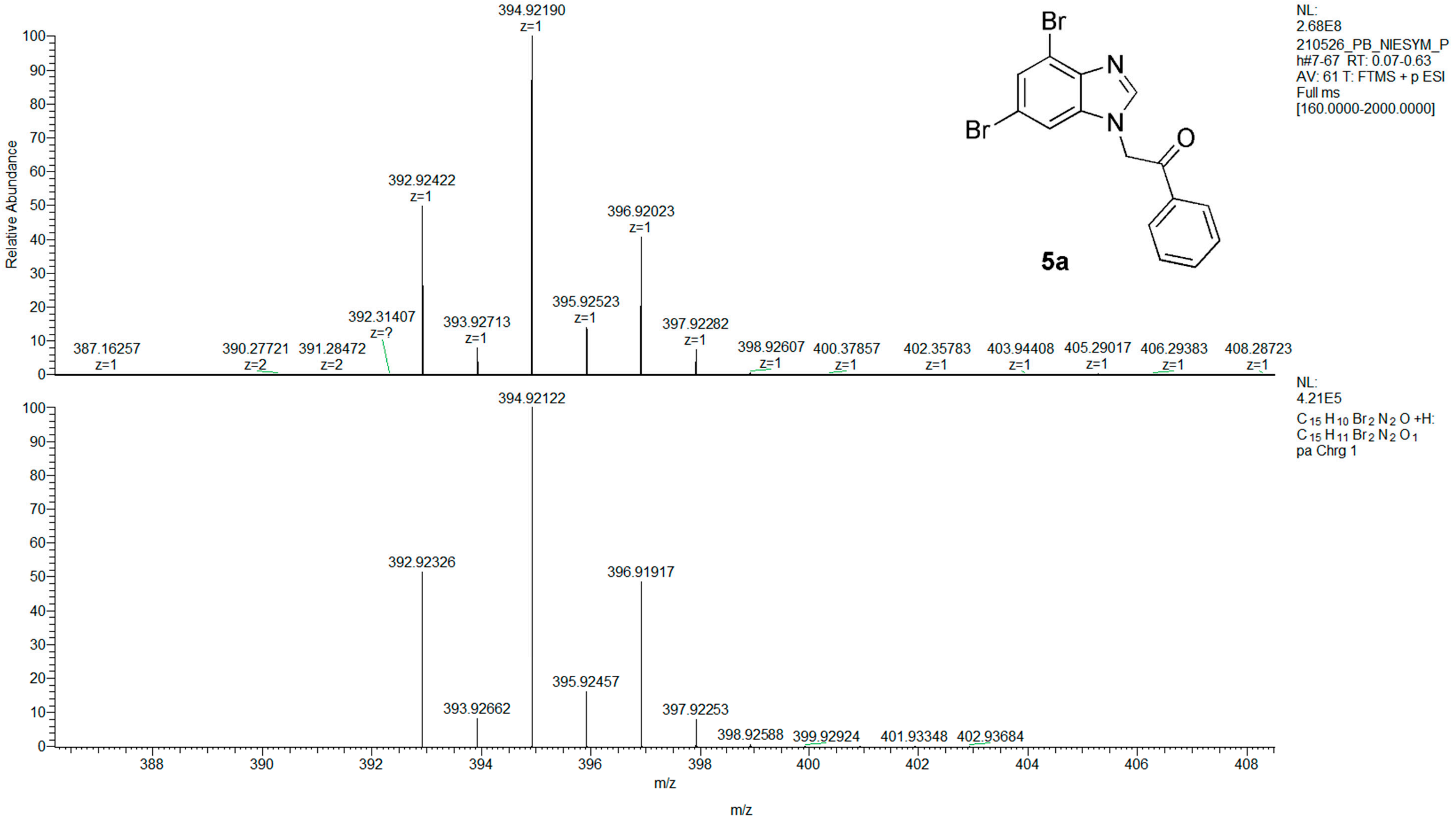

2-(4,6-dibromo-1*H*-benzimidazol-1-yl)-1-(4-chlorophenyl)ethanone (**5c**)

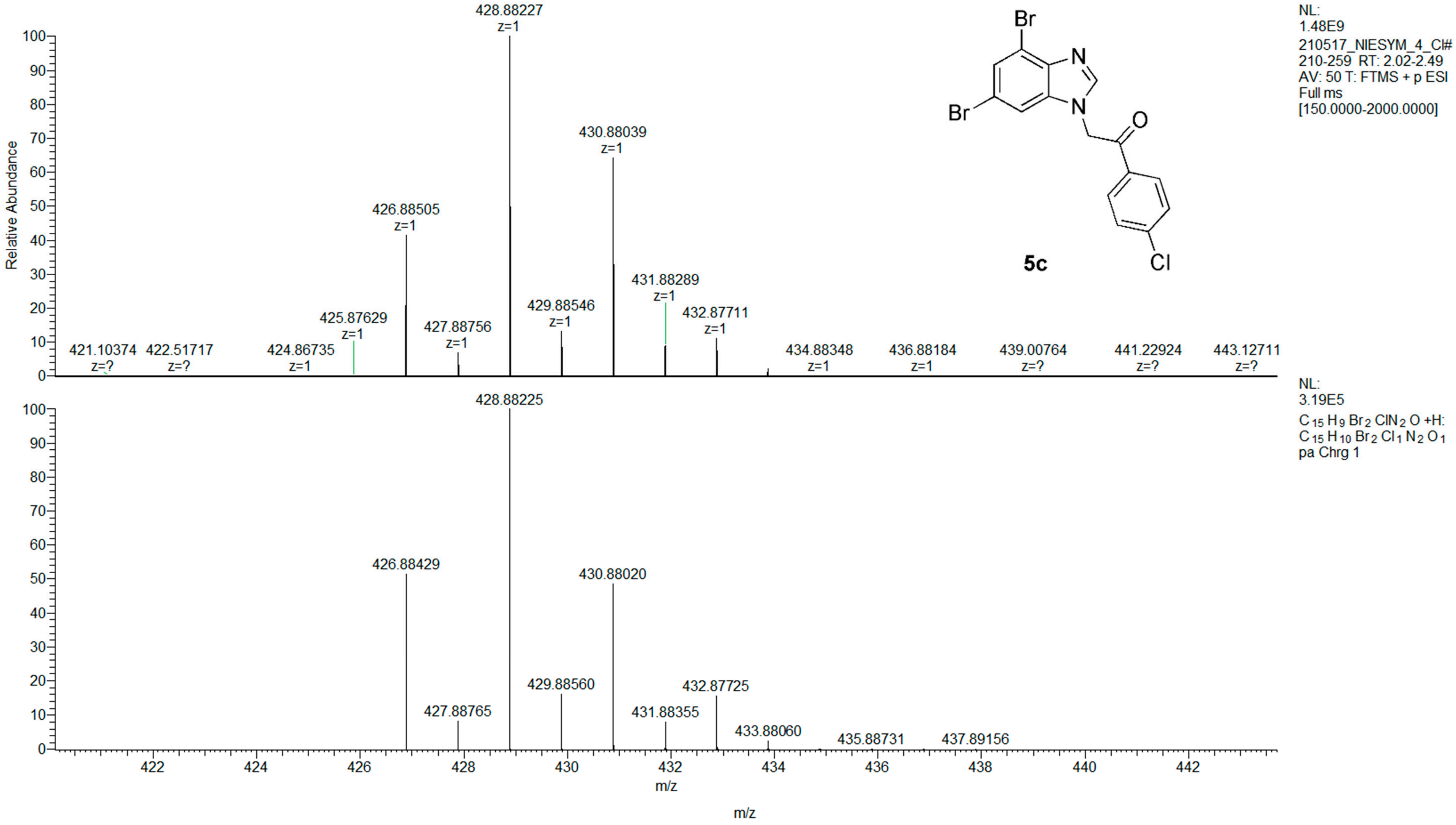

2-(4,6-dibromo-1*H*-benzimidazol-1-yl)-1-(2,4,6-trichlorophenyl)ethanone (5g)

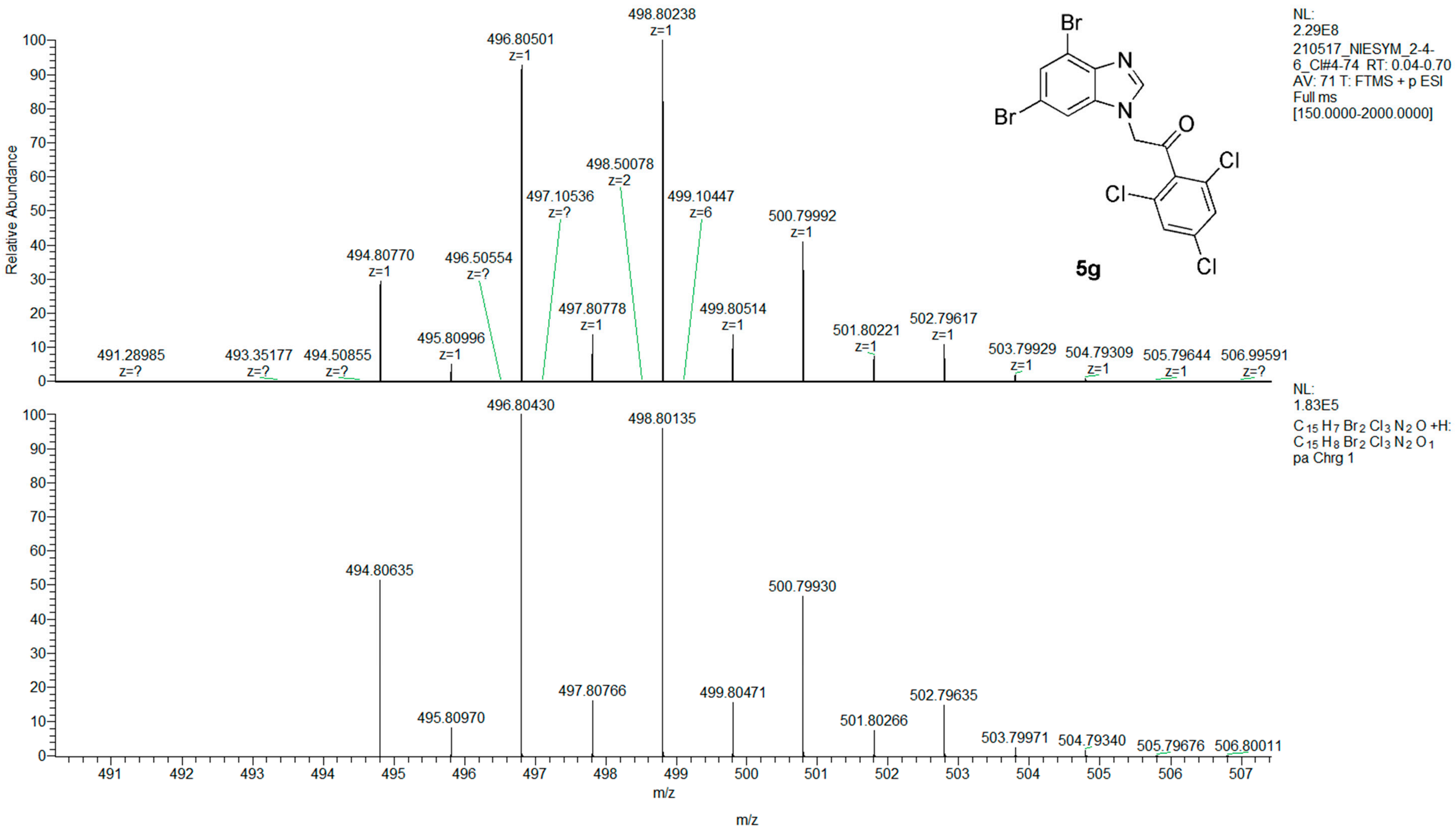

2-(4,6-dibromo-1*H*-benzimidazol-1-yl)-1-(2,5-difluorophenyl)ethanone (5i)

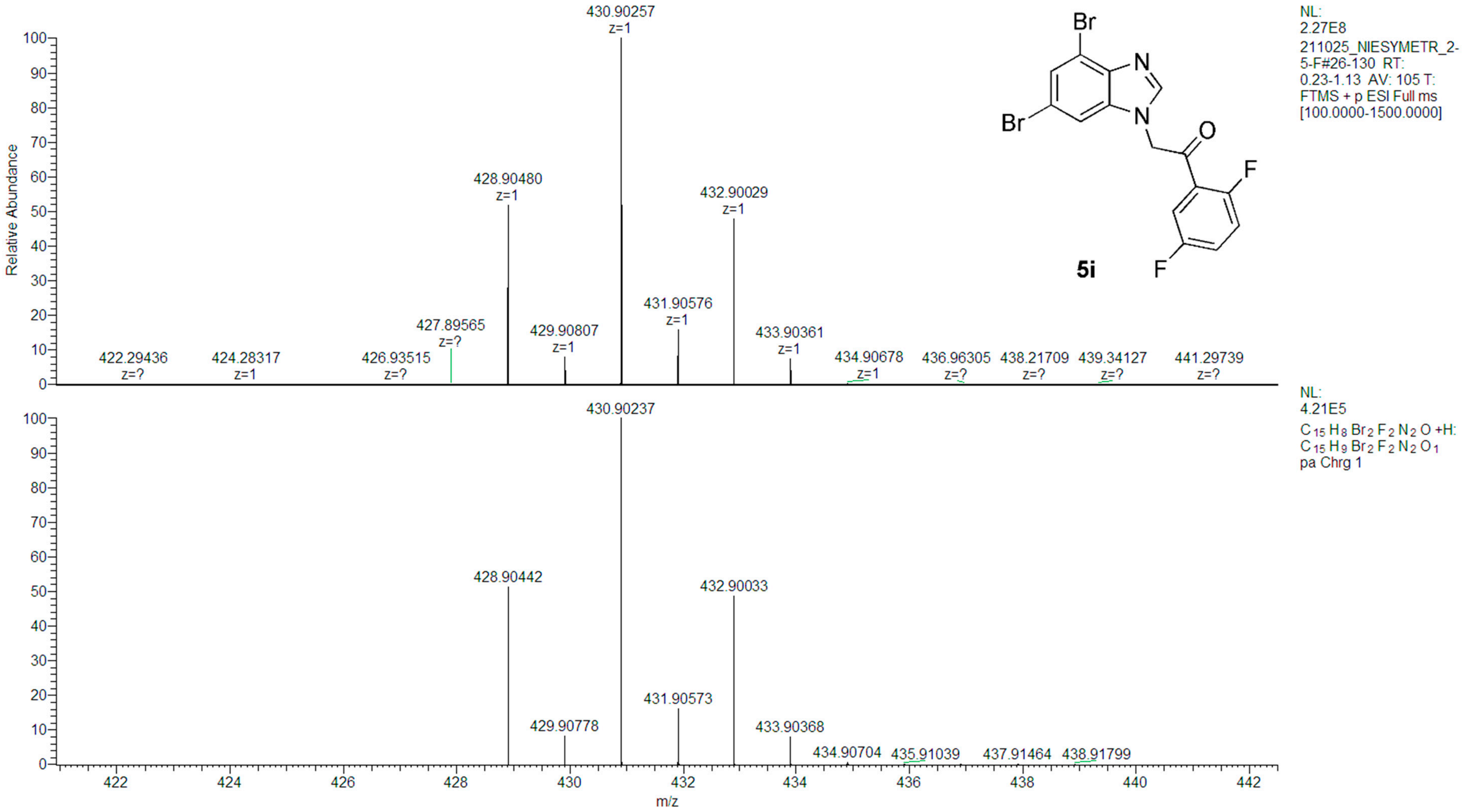

5. Products of 2,4-dichlorophenacyl chloride 3e condensation reaction

To the solution of 2,4-dichlorophenacyl chloride (10 mmol, 2,23 g) in MeCN (50 ml) powdered K_2CO_3 (50 mmol, 6,91 g) was added. The mixture was stirred at rt for 24 h, filtered, evaporated to dryness. The semi-solid orange residue was analyzed by TLC (plate length 15 cm, hexane/EtOAC 95:5).

Five products were found with the following R_f values: 0.61, 0.53, 0.47, 0.36, 0.30. TLC analysis did not reveal the presence of the substrate (R_f 0.52).

The mixture of products (orange semi-solid residue) was separated by column chromatography (the residue was dissolved in hexane/EtOAc 9:1, silica gel, eluent: hexane/ethyl acetate gradient, from 100:0 to 100:3). Sample of pure products with R_f 0.53, 0.36 and 0.30 were isolated and analyzed.

Compound 6e

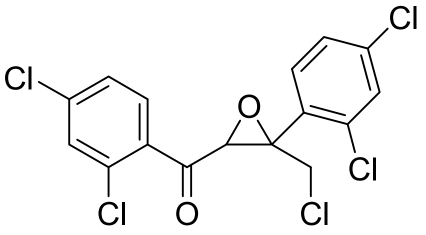

6e

White solid, m.p. 132-133°C; R_f 0.53; ^{1}H NMR (500 MHz, $CDCl_3$) δ= 3.84 (d, J = 11.98 Hz, 1H, C*H*HCl), 4.13 (d, J = 11.98 Hz, 1H, CH*H*Cl), 4.32 (s, 1H, C(O)CH), 7.33 (dd, J = 8.31 Hz, J = 1.96 Hz, 1H, C_6H_2), 7.41 (dd, J = 8.31 Hz, J = 1.96 Hz, 1H, C_6H_2), 7.43 (d, J = 1.96 Hz, 1H, C_6H_2), 7.49 (d, J = 8.31 Hz, 1H, C_6H_2), 7.54 (d, J = 1.96 Hz, 1H, C_6H_2), 7.65 (d, J = 8.31 Hz, 1H, C_6H_2); ^{13}C NMR (125 MHz, $CDCl_3$) δ= 43.38, 65.12, 65.90, 127.29, 127.65, 129.42, 130.85, 130.92, 131.49, 132.61, 132.87, 133.17, 134.75, 135.83, 139.14, 193.62; HRMS: calculated for $C_{16}H_{10}Cl_5O_2$ $[M+H]^+$: 410.90884. Found: 410.90881.

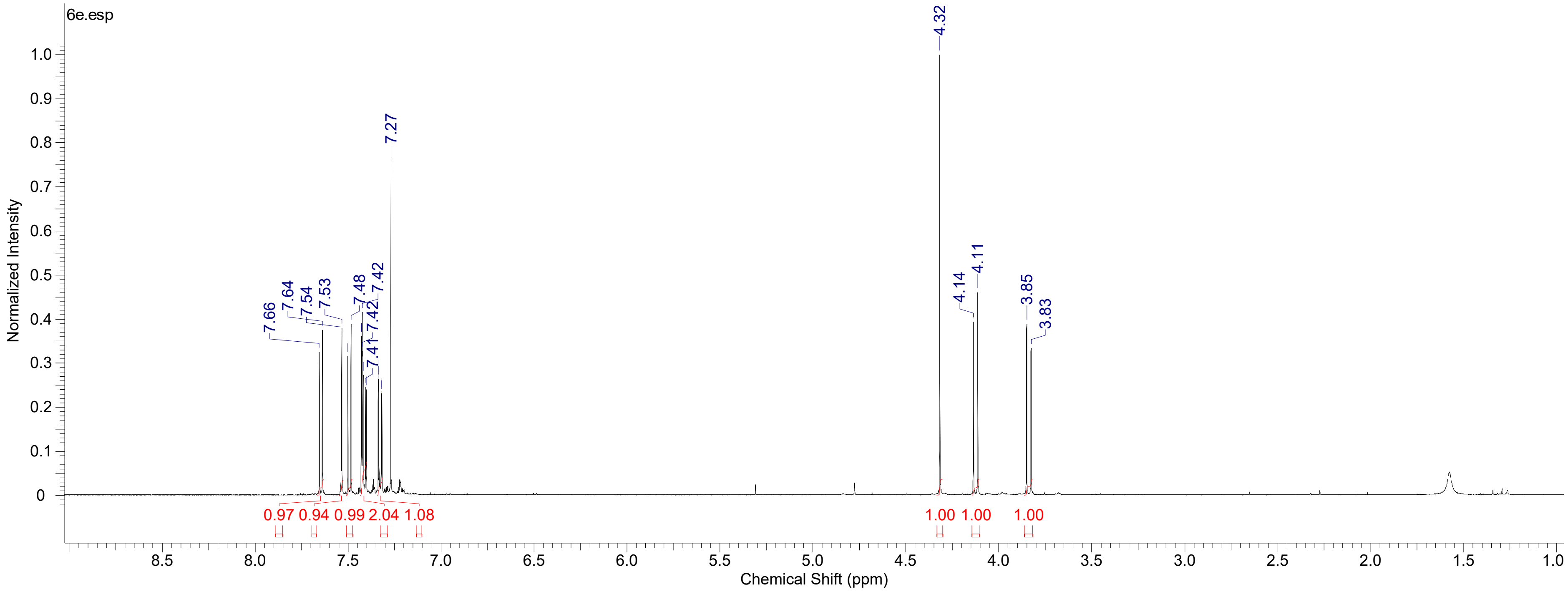
6e.esp
Normalized Intensity
Chemical Shift (ppm)
7.66
7.64
7.54
7.53
7.48
7.42
7.42
7.41
7.27
4.32
4.14
4.11
3.85
3.83
0.97
0.94
0.99
2.04
1.08
1.00
1.00
1.00

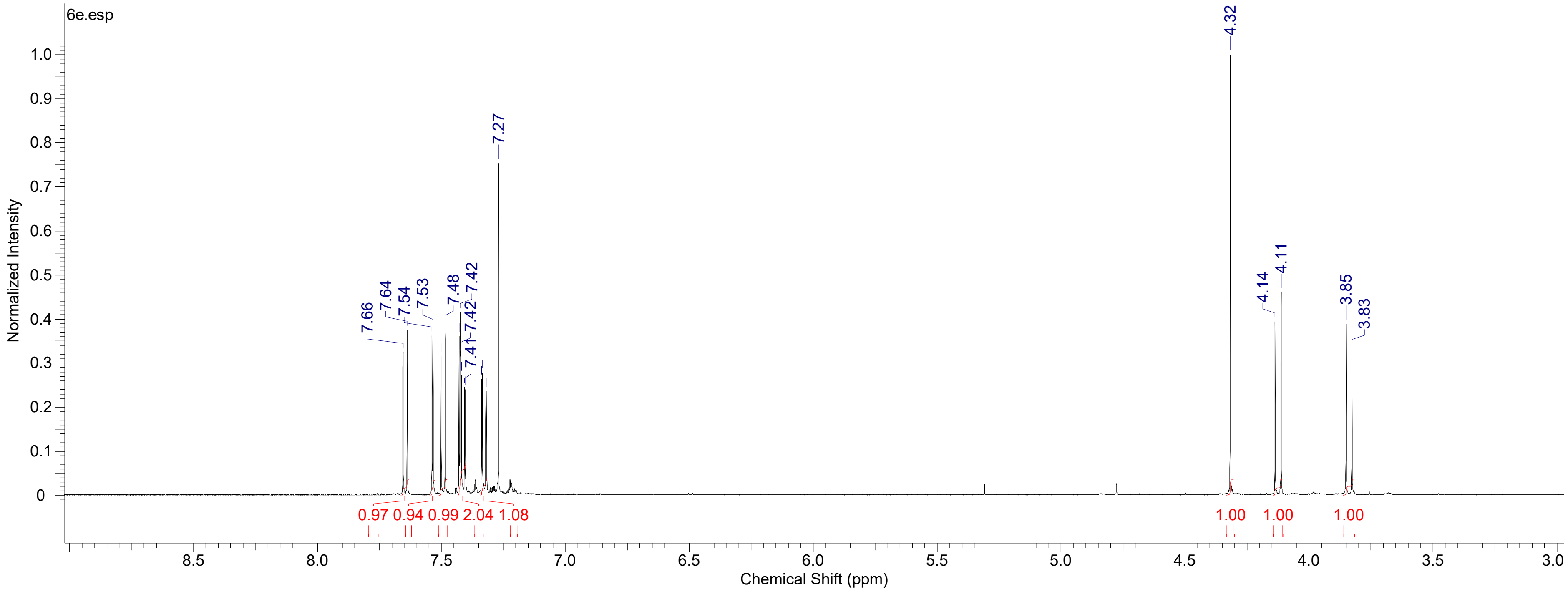
6e.esp
Normalized Intensity
Chemical Shift (ppm)
7.66
7.64
7.54
7.53
7.48
7.42
7.42
7.41
7.27
4.32
4.14
4.11
3.85
3.83
0.97
0.94
0.99
2.04
1.08
1.00
1.00
1.00

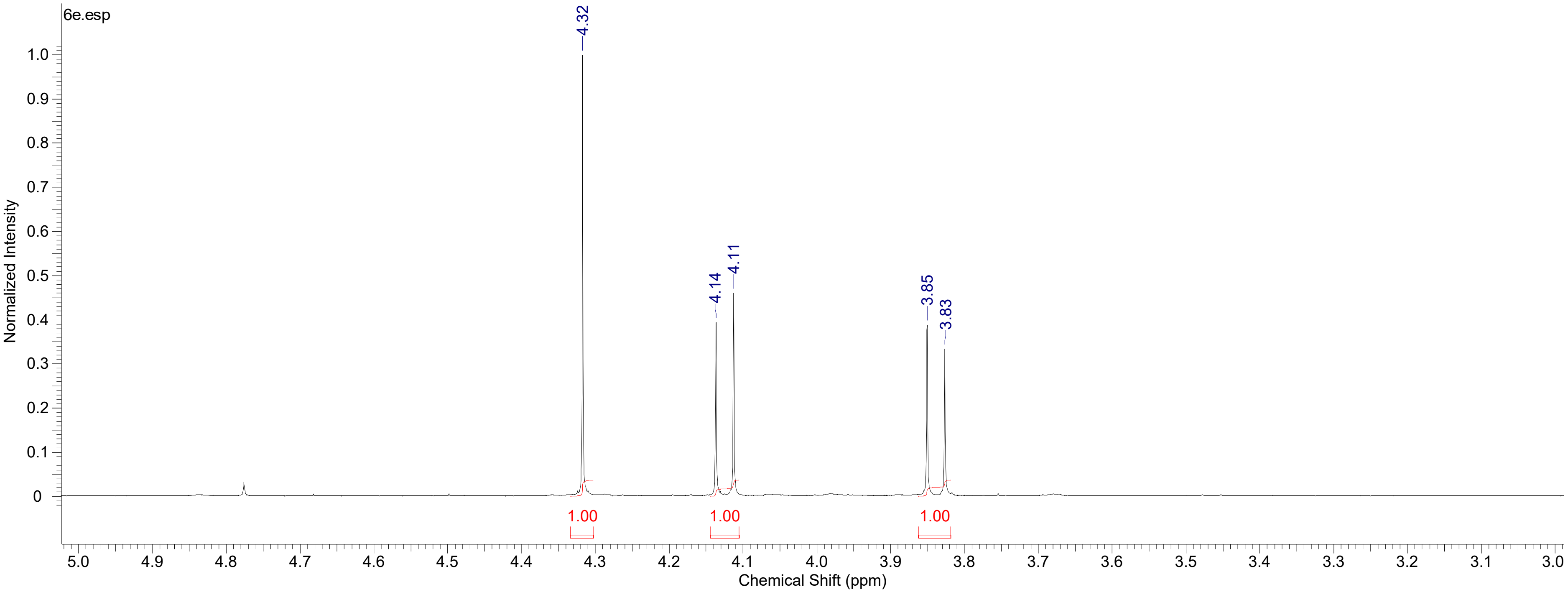

6e.esp
4.32
4.14
4.11
3.85
3.83
1.00
1.00
1.00
Normalized Intensity
Chemical Shift (ppm)

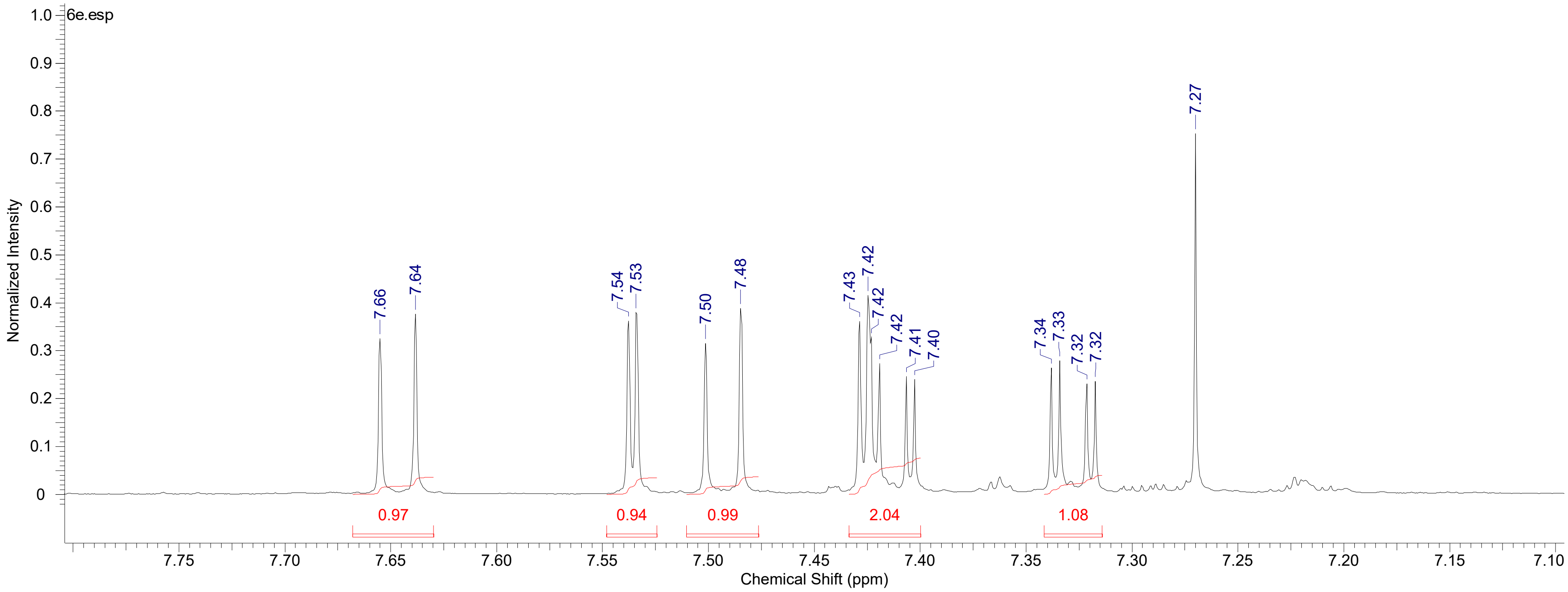
6e.esp
Normalized Intensity
Chemical Shift (ppm)
7.66
7.64
7.54
7.53
7.50
7.48
7.43
7.42
7.42
7.42
7.41
7.40
7.34
7.33
7.32
7.32
7.27
0.97
0.94
0.99
2.04
1.08

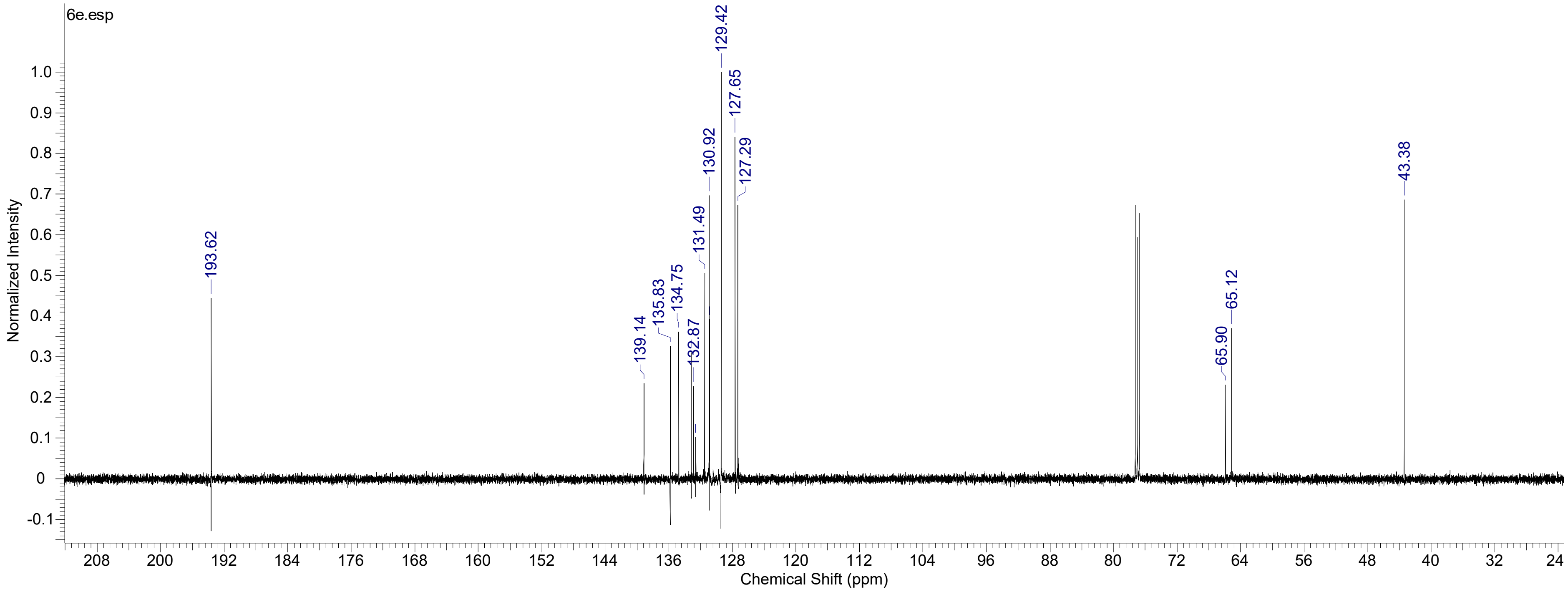

6e.esp
193.62
139.14
135.83
134.75
132.87
131.49
130.92
129.42
127.65
127.29
65.90
65.12
43.38
Normalized Intensity
Chemical Shift (ppm)

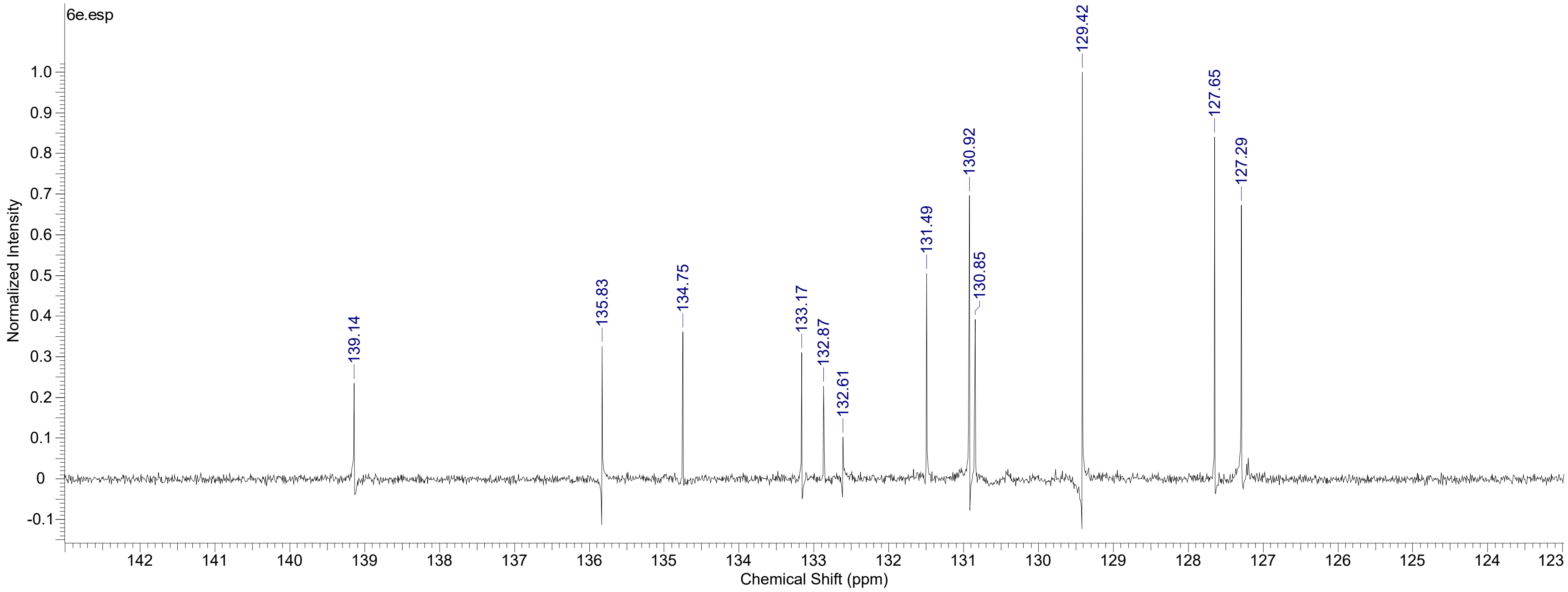

6e.esp
139.14
135.83
134.75
133.17
132.87
132.61
131.49
130.92
130.85
129.42
127.65
127.29
Normalized Intensity
Chemical Shift (ppm)

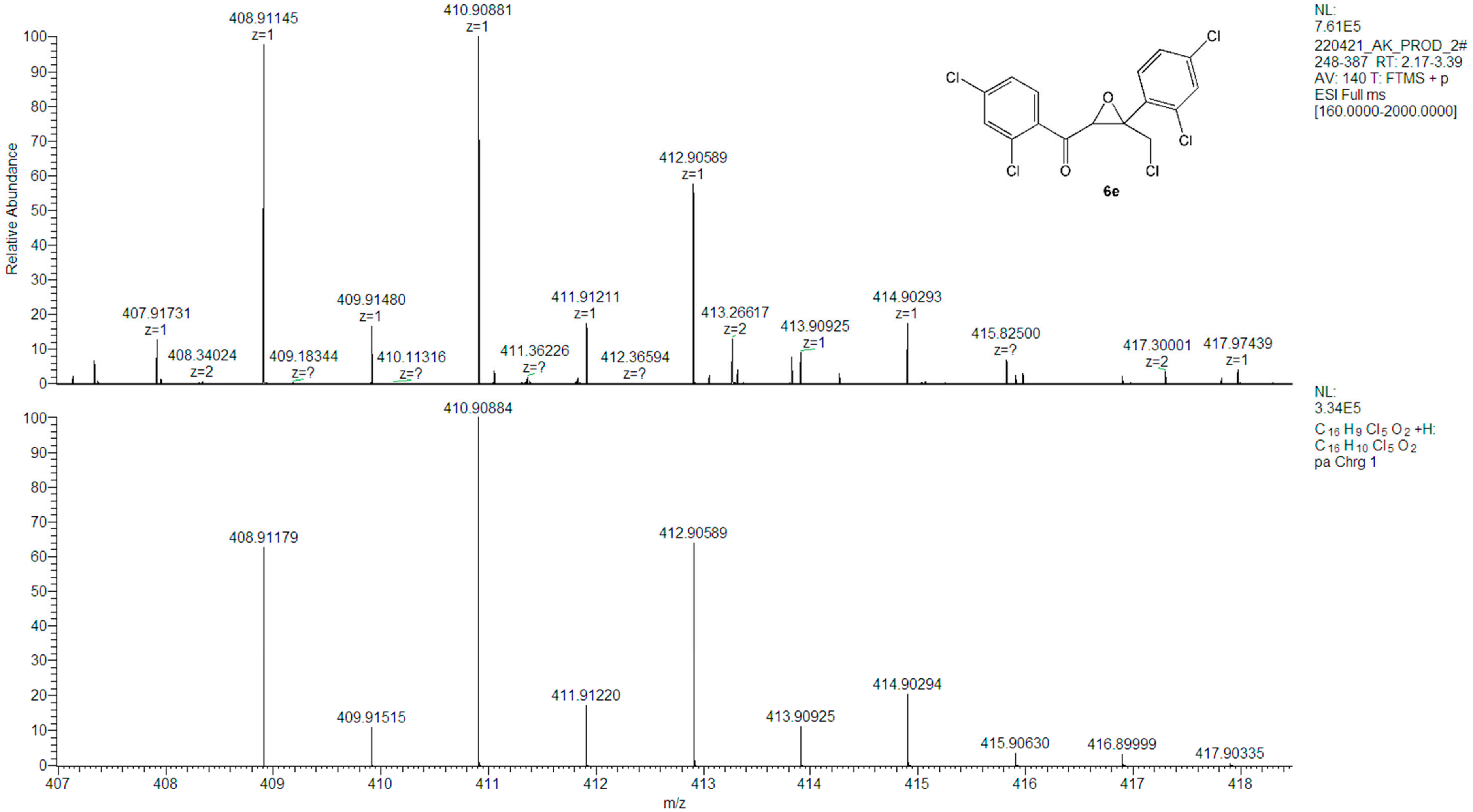

NL:
7.61E5
220421_AK_PROD_2#
248-387 RT: 2.17-3.39
AV: 140 T: FTMS + p
ESI Full ms
[160.0000-2000.0000]
6e
408.91145 z=1
410.90881 z=1
412.90589 z=1
407.91731 z=1
408.34024 z=2
409.18344 z=?
409.91480 z=1
410.11316 z=?
411.36226 z=?
411.91211 z=1
412.36594 z=?
413.26617 z=2
413.90925 z=1
414.90293 z=1
415.82500 z=?
417.30001 z=2
417.97439 z=1
NL:
3.34E5
C 16 H 9 Cl 5 O 2 +H:
C 16 H 10 Cl 5 O 2
pa Chrg 1
410.90884
408.91179
412.90589
409.91515
411.91220
413.90925
414.90294
415.90630
416.89999
417.90335
Relative Abundance
m/z

Compound 7e

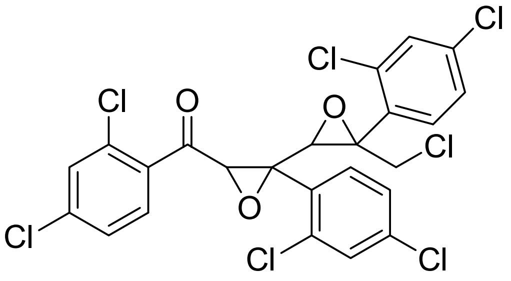

7e

White solid, m.p. 104-106°C; R_f 0.47; ^{1}H NMR (500 MHz, $CDCl_3$) δ= 3.95 (s, 1H, CH-O), 4.16 (d, *J* = 11.49 Hz, 1H, C*H*HCl), 4.37 (d, *J* = 11.49 Hz, 1H, CH*H*Cl), 4.69 (s, 1H, CH-O), 7.22-7.28 (m, 6H, C_6H_2), 7.36-7.40 (m, 1H, C_6H_2), 7.37 (d, *J* = 1.96 Hz, 1H, C_6H_2), 7.42 (d, *J* = 1.96 Hz, 1H, C_6H_2); ^{13}C NMR (125 MHz, $CDCl_3$) δ= 23.25, 24.94, 25.73, 27.64, 89.59, 89.64, 90.08, 91.53, 91.98, 93.02, 93.49, 93.62, 93.78, 94.91, 95.96, 95.99, 96.63, 97.92, 98.69, 101.32; HRMS: calculated for $C_{24}H_{14}Cl_7O_3$ $[M+H]^+$: 598.86981. Found: 596.86967.

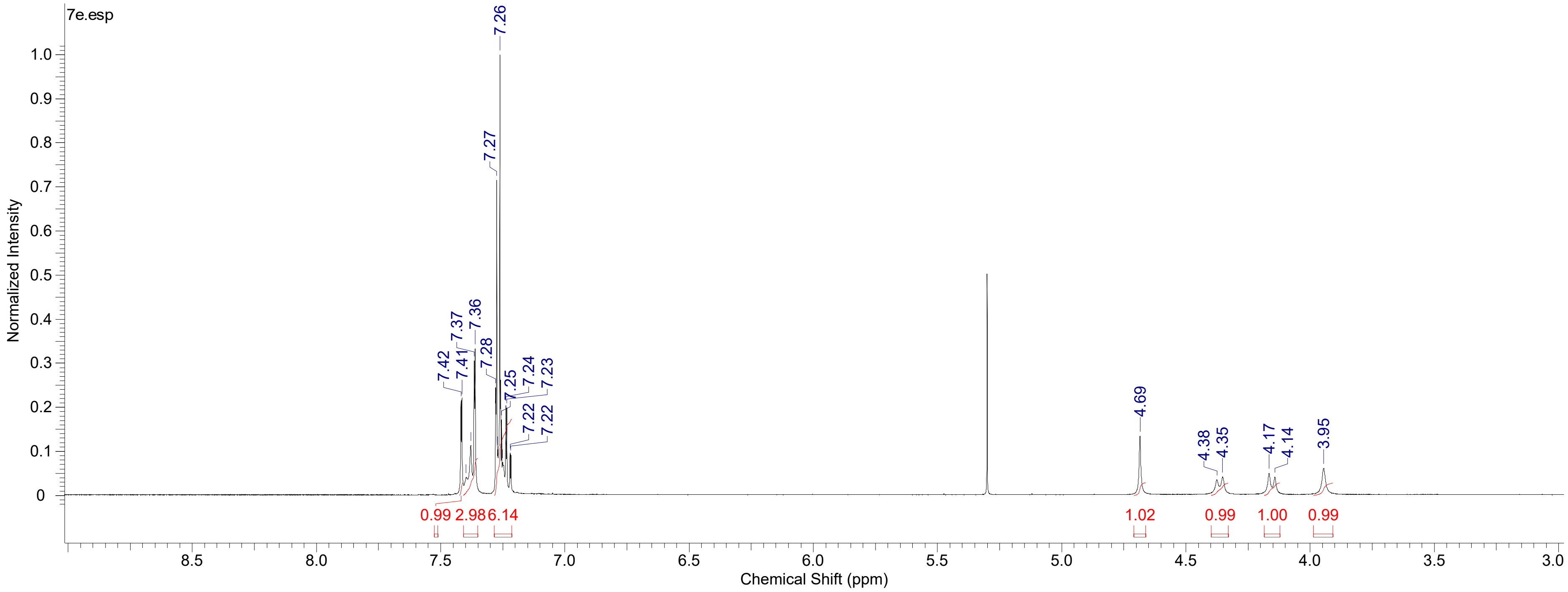
7e.esp
Normalized Intensity
Chemical Shift (ppm)
7.26
7.27
7.42
7.41
7.37
7.36
7.28
7.25
7.24
7.23
7.22
7.22
4.69
4.38
4.35
4.17
4.14
3.95
0.99
2.98
6.14
1.02
0.99
1.00
0.99

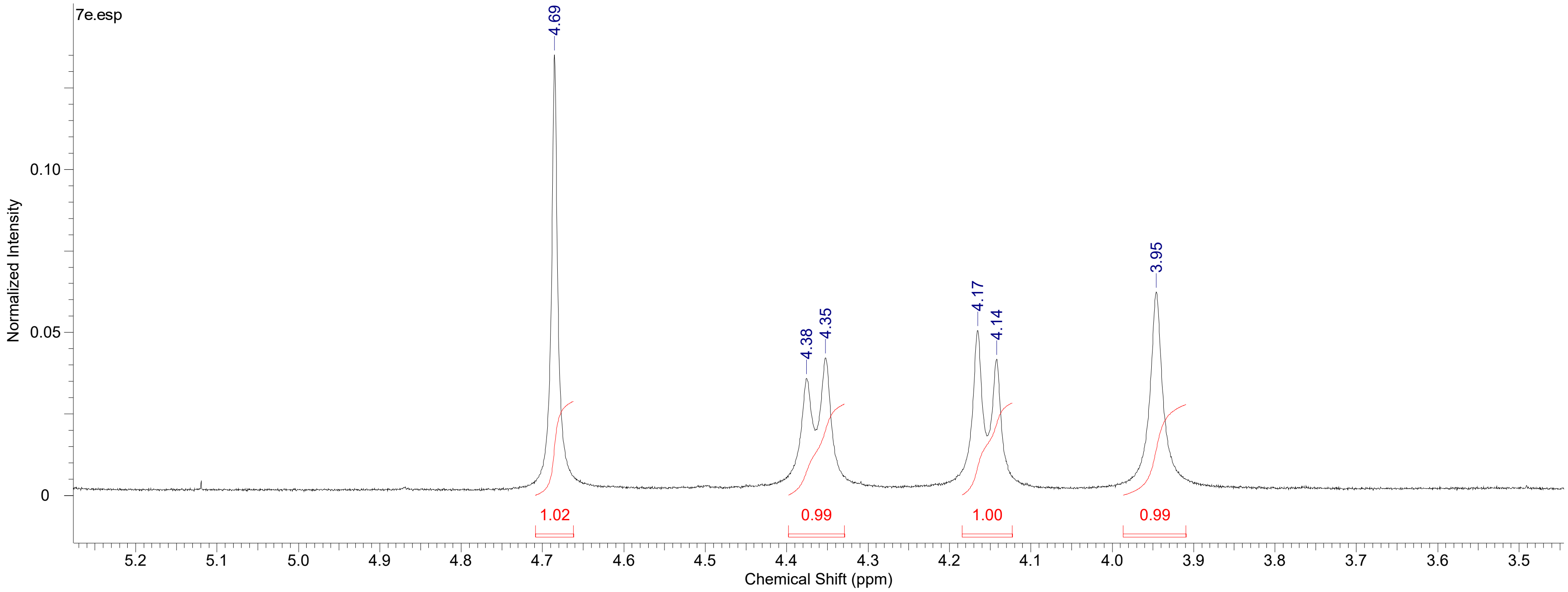
7e.esp
4.69
4.38
4.35
4.17
4.14
3.95
1.02
0.99
1.00
0.99
Normalized Intensity
0.10
0.05
0
5.2
5.1
5.0
4.9
4.8
4.7
4.6
4.5
4.4
4.3
4.2
4.1
4.0
3.9
3.8
3.7
3.6
3.5
Chemical Shift (ppm)

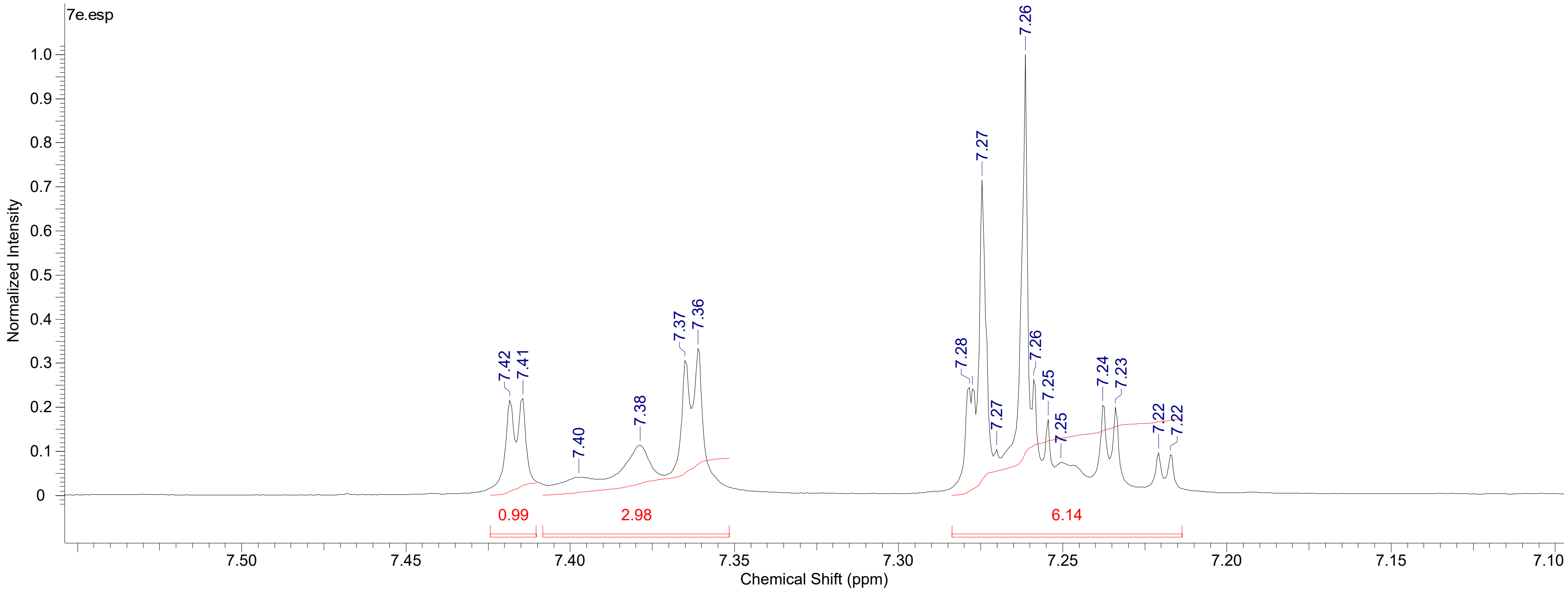
7e.esp
Normalized Intensity
Chemical Shift (ppm)
7.42
7.41
7.40
7.38
7.37
7.36
7.28
7.27
7.27
7.26
7.26
7.25
7.25
7.24
7.23
7.22
7.22
0.99
2.98
6.14

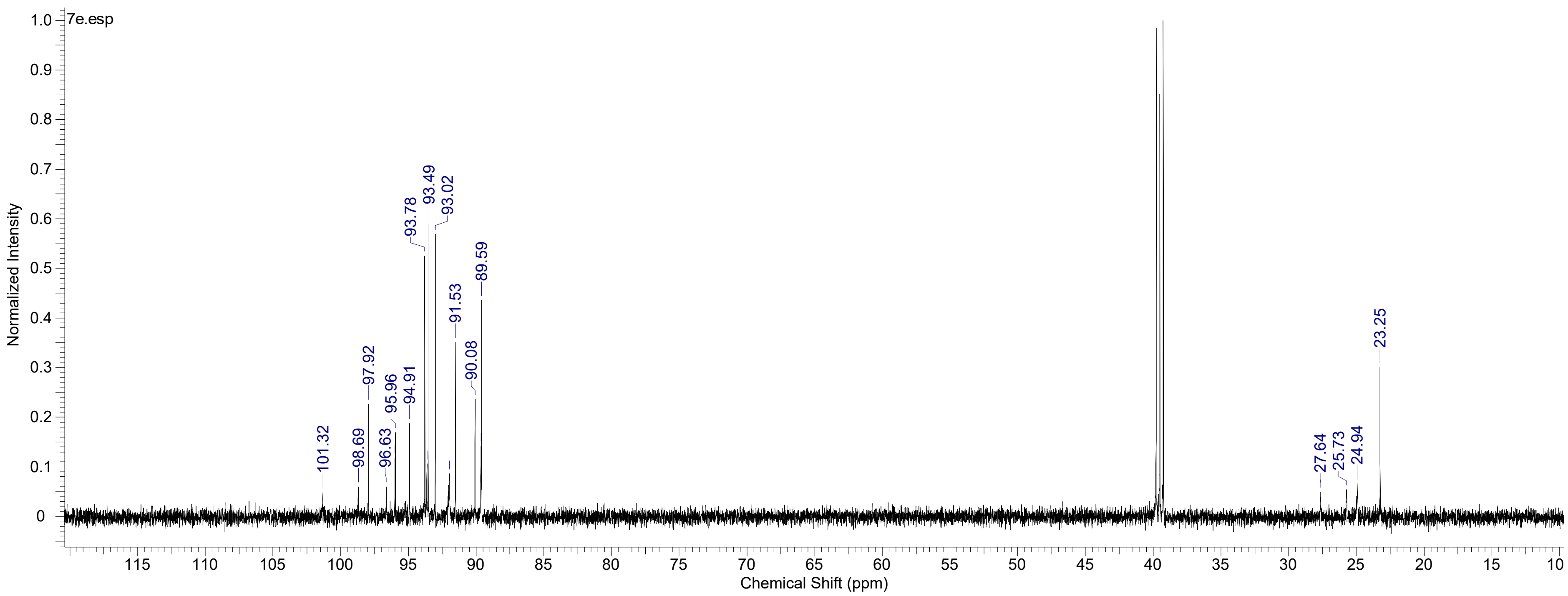

7e.esp
Normalized Intensity
Chemical Shift (ppm)
101.32
98.69
97.92
96.63
95.96
94.91
93.78
93.49
93.02
91.53
90.08
89.59
27.64
25.73
24.94
23.25

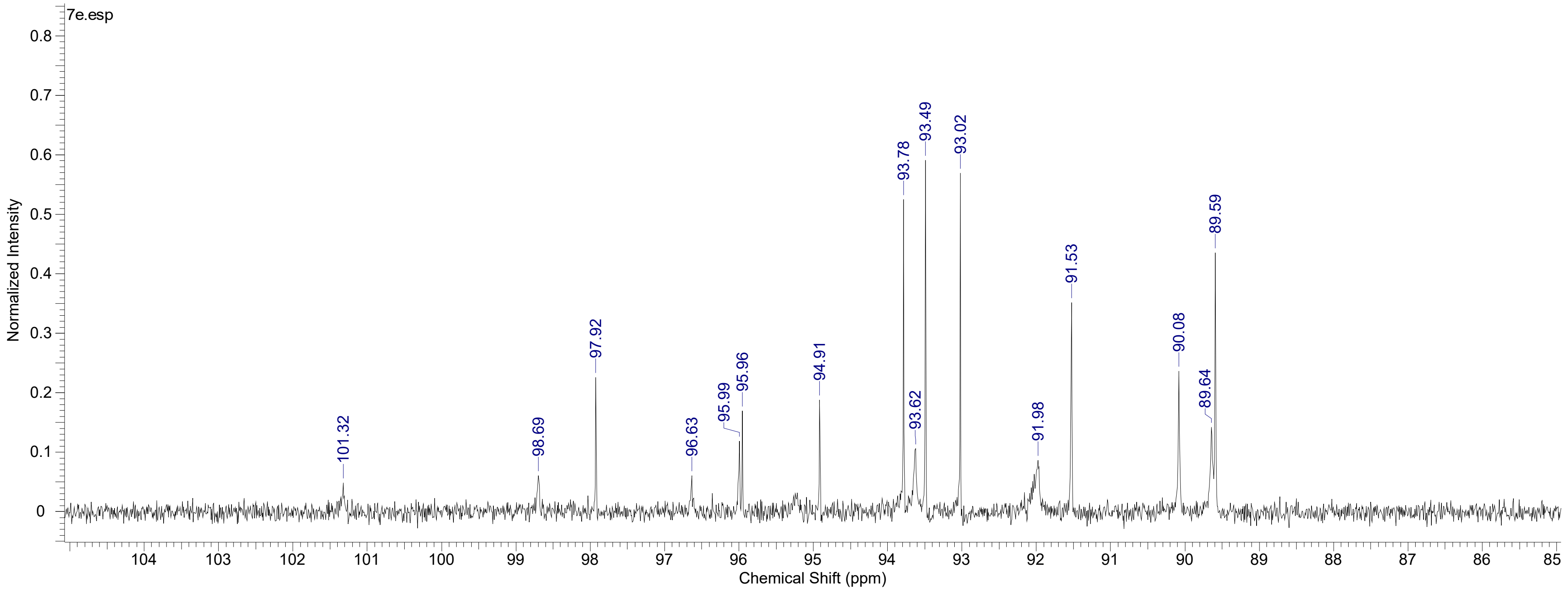
7e.esp
Normalized Intensity
Chemical Shift (ppm)
101.32
98.69
97.92
96.63
95.99
95.96
94.91
93.78
93.62
93.49
93.02
91.98
91.53
90.08
89.64
89.59

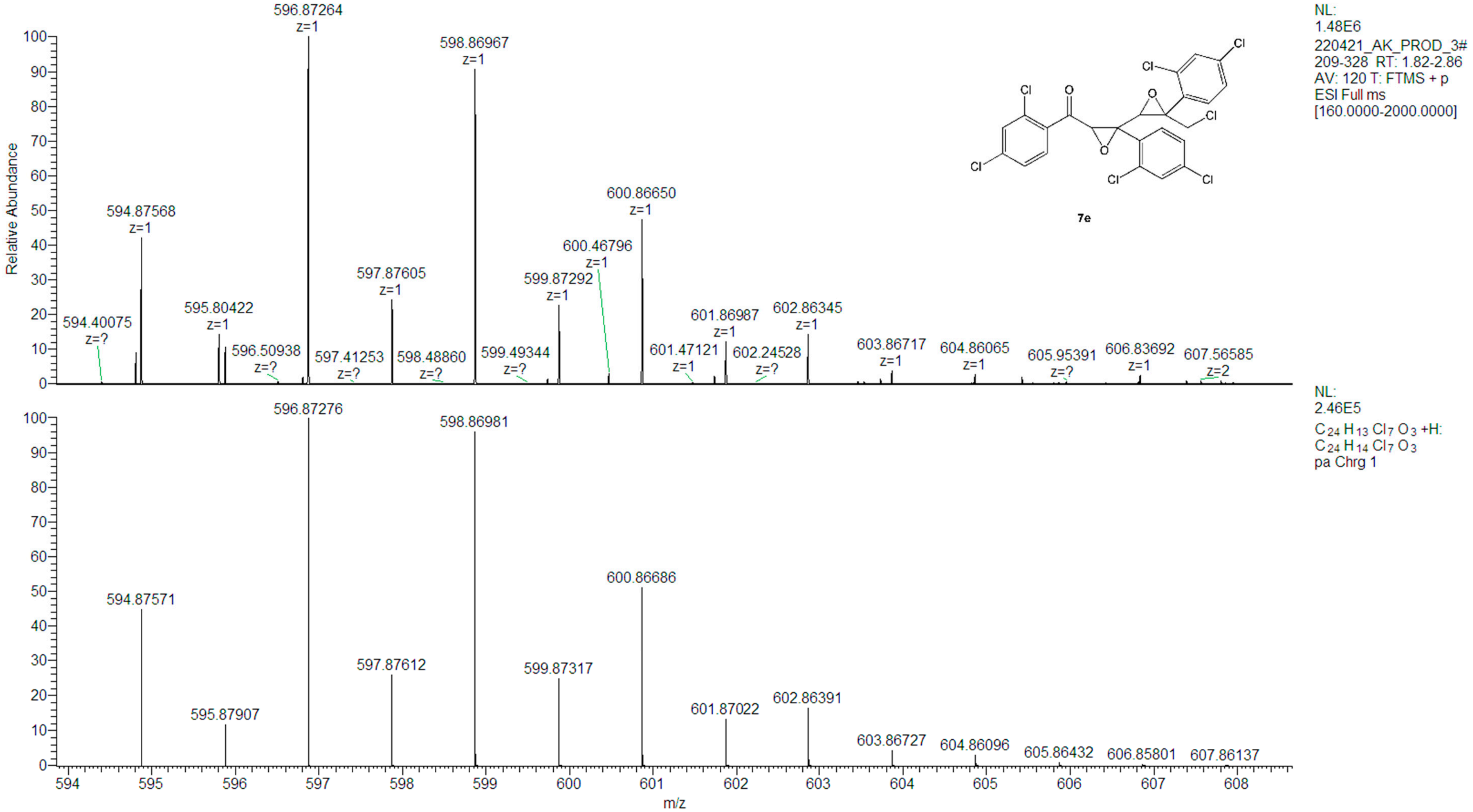
NL:
1.48E6
220421_AK_PROD_3#
209-328 RT: 1.82-2.86
AV: 120 T: FTMS + p
ESI Full ms
[160.0000-2000.0000]
7e
Relative Abundance
596.87264 z=1
598.86967 z=1
600.86650 z=1
600.46796 z=1
594.87568 z=1
597.87605 z=1
599.87292 z=1
595.80422 z=1
594.40075 z=?
601.86987 z=1
602.86345 z=1
596.50938 z=?
597.41253 z=?
598.48860 z=?
599.49344 z=?
601.47121 z=1
602.24528 z=?
603.86717 z=1
604.86065 z=1
605.95391 z=?
606.83692 z=1
607.56585 z=2
NL:
2.46E5
$C_{24}H_{13}Cl_7O_3$ +H:
$C_{24}H_{14}Cl_7O_3$
pa Chrg 1
596.87276
598.86981
600.86686
594.87571
597.87612
599.87317
595.87907
601.87022
602.86391
603.86727
604.86096
605.86432
606.85801
607.86137
m/z

Compound 8e

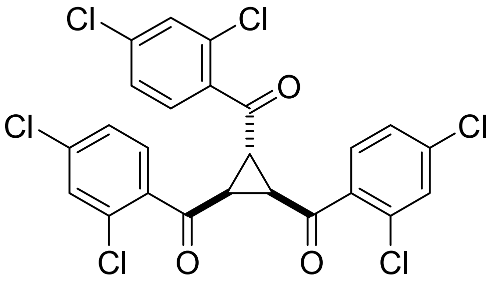

8e

White solid, m.p. 122-123°C; R_f 0.30; ^{1}H NMR (500 MHz, $CDCl_3$) δ = 3.73 (d, *J* = 5.62 Hz, 2H, C_3*HH*H *cis*), 3.87 (dd, *J* = 5.87 Hz, *J* = 5.38 Hz, 1H, C_3HH*H*), 7.34 (dd, *J* = 8.56 Hz, *J* = 1.96 Hz, 2H, C_6H_2), 7.39 (dd, *J* = 8.56 Hz, *J* = 1.96 Hz, 1H, C_6H_2), 7.44 (d, *J* = 1.96 Hz, 2H, C_6H_2), 7.49 (d, *J* = 1.71 Hz, 1H, C_6H_2), 7.58 (d, *J* = 8.31 Hz, 2H, C_6H_2), 7.63 (d, *J* = 8.31 Hz, 2H, C_6H_2); ^{13}C NMR (125 MHz, $CDCl_3$) δ = 36.76, 41.46, 127.60, 127.62, 130.51, 130.80, 121.17, 131.69, 132.89, 133.13, 135.87136.03, 138.64, 138.77, 193.96, 196.15; HRMS: calculated for $C_{24}H_{13}Cl_6O_3$ $[M+H]^+$: 562.89314. Found: 562.89302.

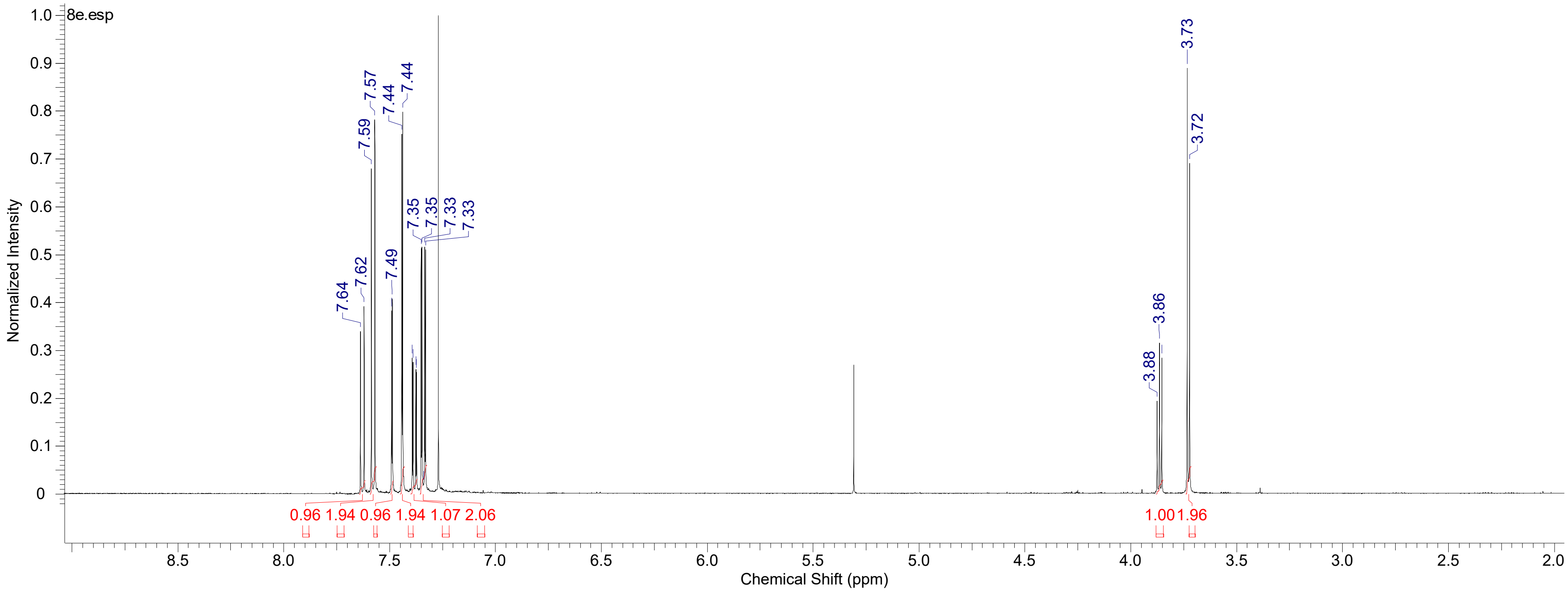
8e.esp
Normalized Intensity
Chemical Shift (ppm)
7.64
7.62
7.59
7.57
7.49
7.44
7.44
7.35
7.35
7.33
7.33
3.88
3.86
3.73
3.72
0.96 1.94 0.96 1.94 1.07 2.06
1.00 1.96

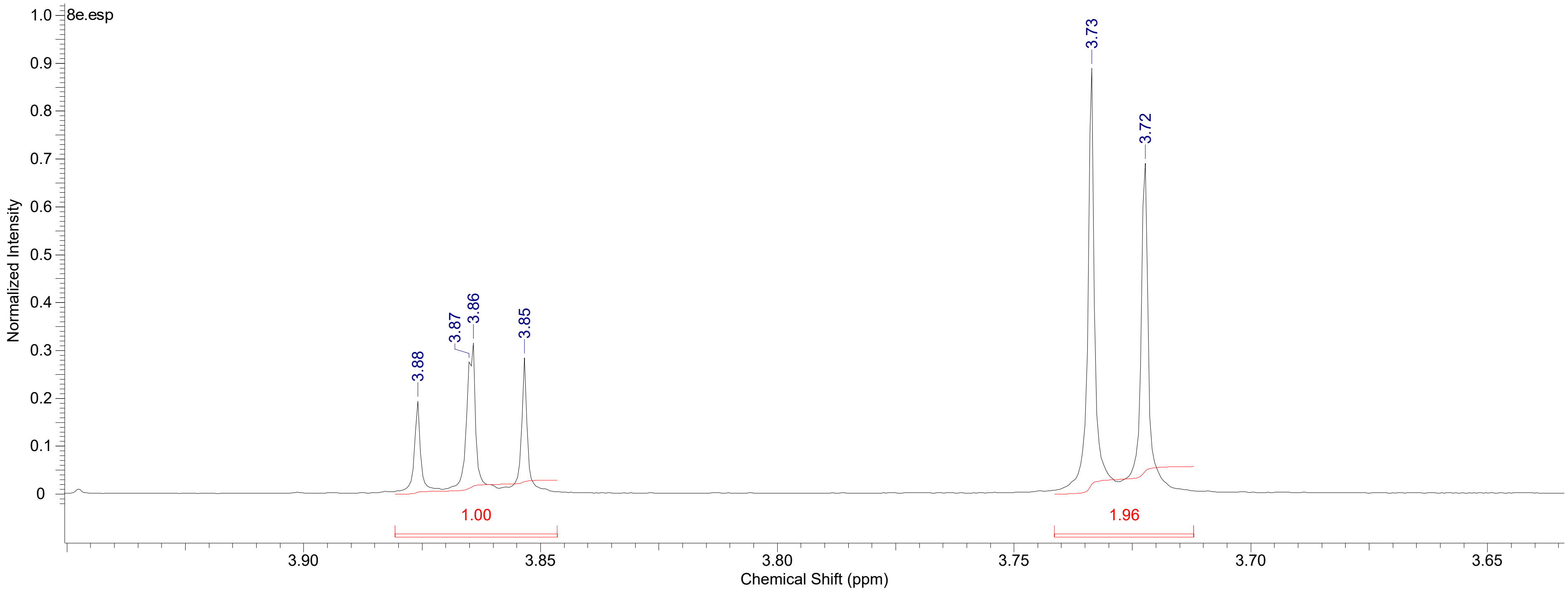

8e.esp
3.88
3.87
3.86
3.85
3.73
3.72
1.00
1.96
Normalized Intensity
Chemical Shift (ppm)

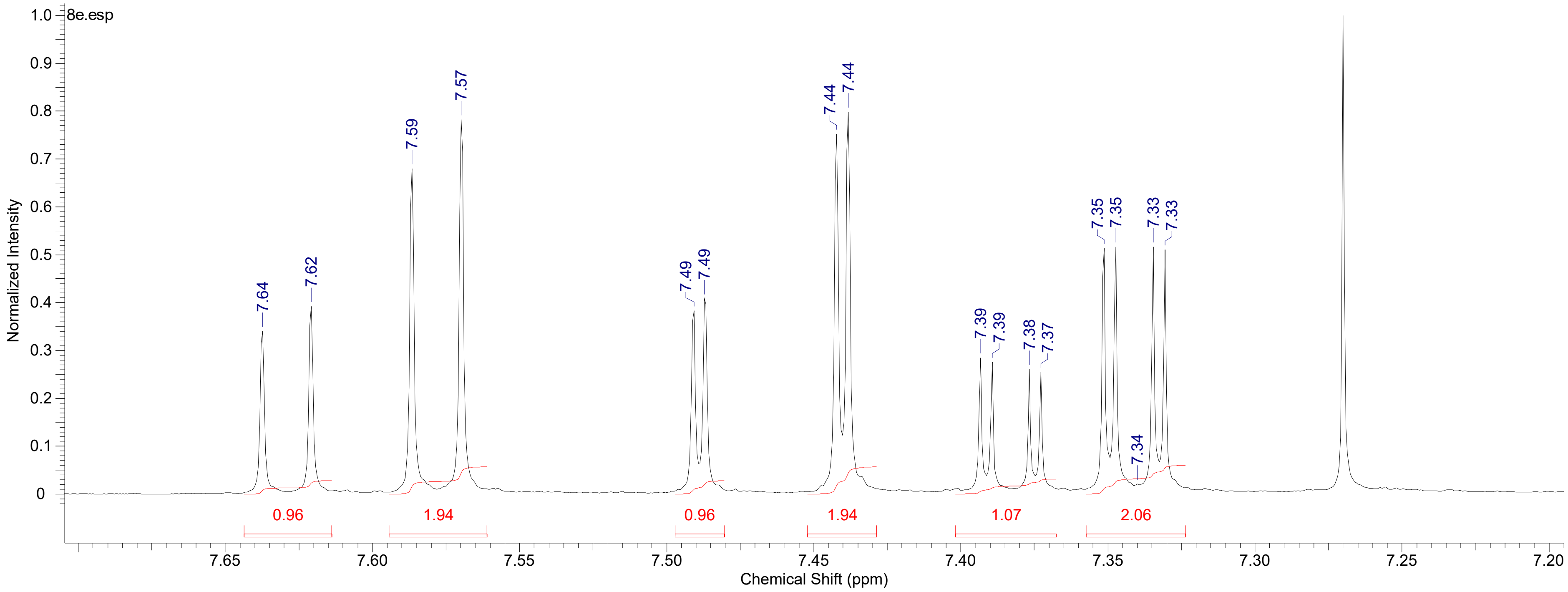

8e.esp
Normalized Intensity
Chemical Shift (ppm)
7.64
7.62
7.59
7.57
7.49
7.49
7.44
7.44
7.39
7.39
7.38
7.37
7.35
7.35
7.34
7.33
7.33
0.96
1.94
0.96
1.94
1.07
2.06

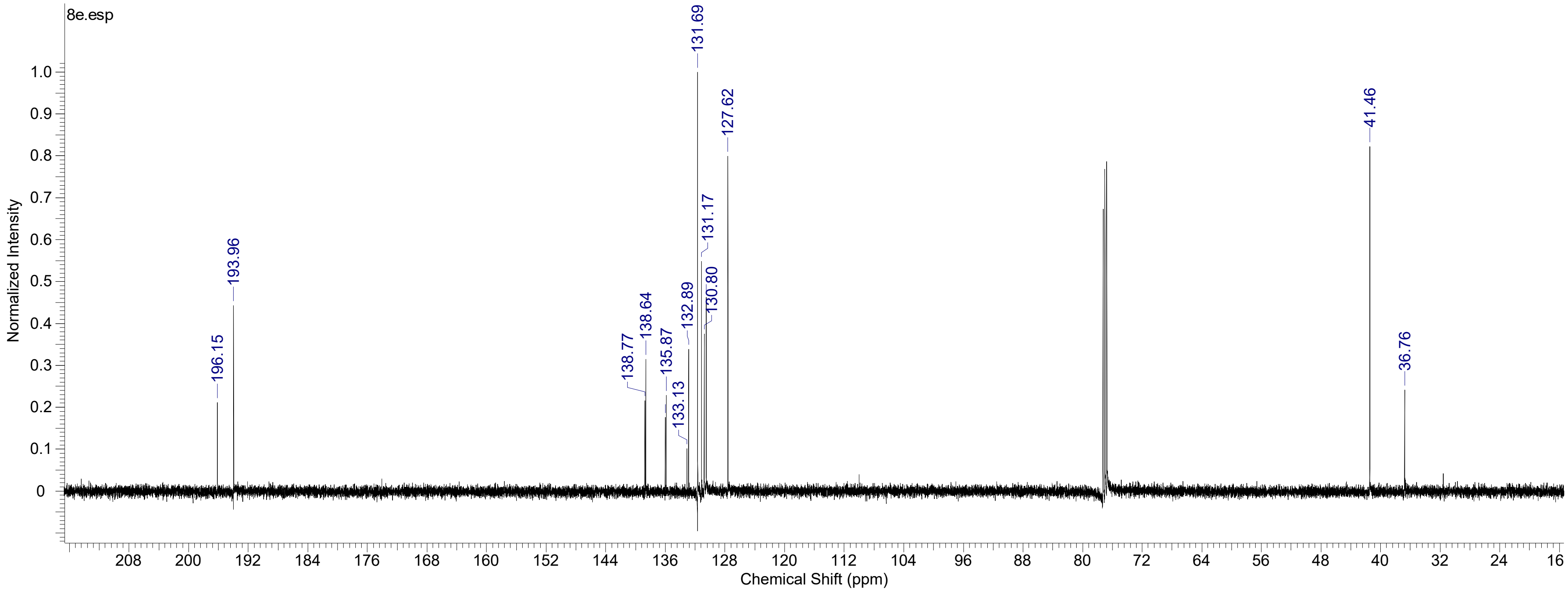
8e.esp
196.15
193.96
138.77
138.64
135.87
133.13
132.89
131.69
131.17
130.80
127.62
41.46
36.76
Normalized Intensity
Chemical Shift (ppm)

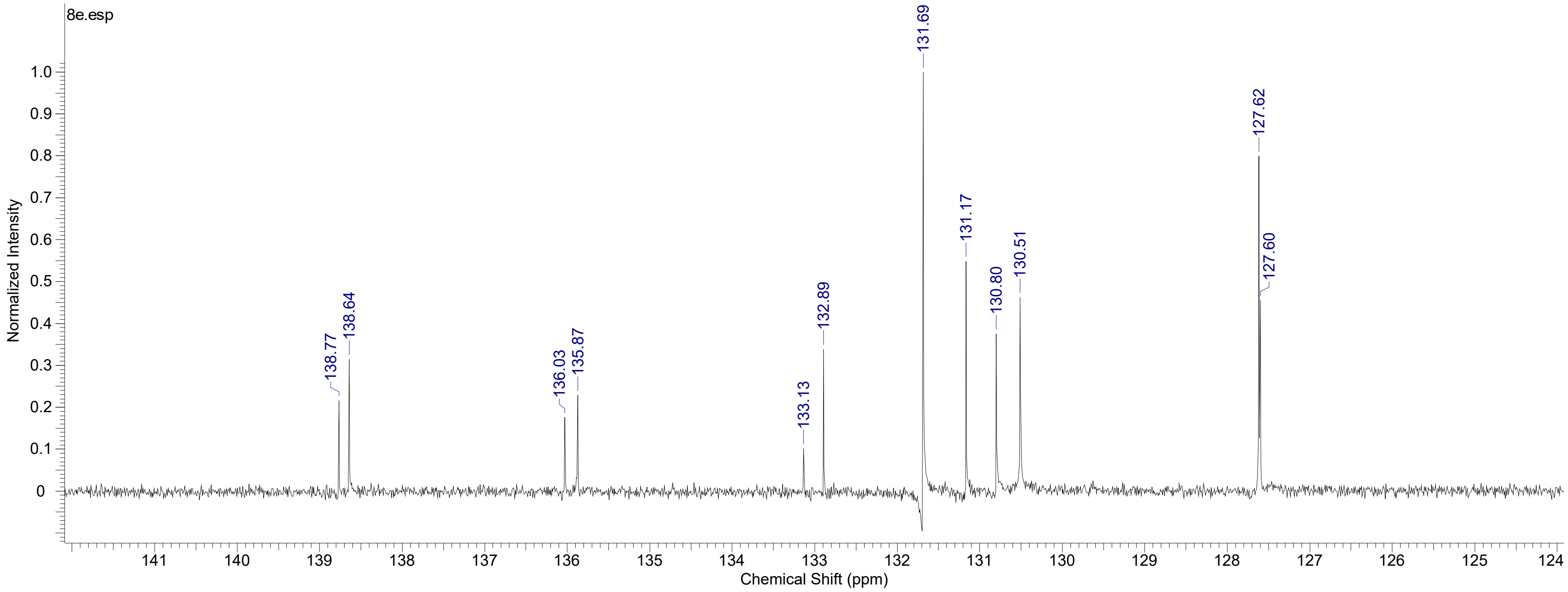
8e.esp
Normalized Intensity
Chemical Shift (ppm)
138.77
138.64
136.03
135.87
133.13
132.89
131.69
131.17
130.80
130.51
127.62
127.60

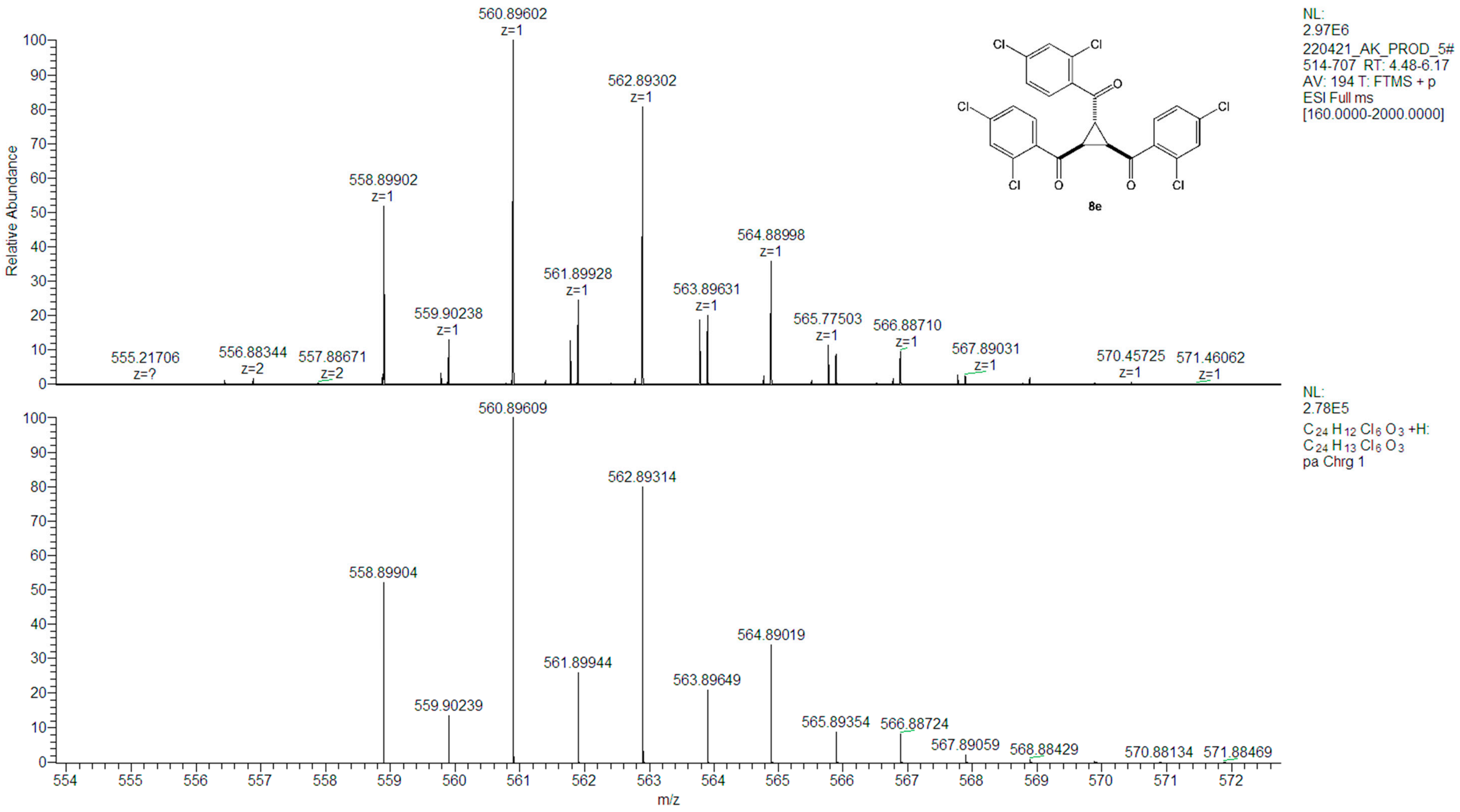

560.89602
z=1
562.89302
z=1
558.89902
z=1
564.88998
z=1
561.89928
z=1
563.89631
z=1
559.90238
z=1
565.77503
z=1
566.88710
z=1
567.89031
z=1
555.21706
z=?
556.88344
z=2
557.88671
z=2
570.45725
z=1
571.46062
z=1
Relative Abundance
8e
NL:
2.97E6
220421_AK_PROD_5#
514-707 RT: 4.48-6.17
AV: 194 T: FTMS + p
ESI Full ms
[160.0000-2000.0000]
560.89609
562.89314
558.89904
564.89019
561.89944
563.89649
559.90239
565.89354
566.88724
567.89059
568.88429
570.88134
571.88469
m/z
NL:
2.78E5
C 24 H 12 Cl 6 O 3 +H:
C 24 H 13 Cl 6 O 3
pa Chrg 1

6. X-ray analysis of compound 5d

X-ray experiment

Clear colorless crystal of 1-(4-bromophenyl)-2-(4,6-dibromo-1*H*-benzimidazol-1-yl)ethanone was placed on an Xcalibur R four circle diffractometer from Oxford Diffraction. After an initial crystal checking the approximate unit cell parameters, crystal system and symmetry were determined using the monochromatic Cu Kα radiation. Consecutively 44722 reflections were collected up to 2θ=142.52°. After initial corrections the data were used to solve and refine the crystal and molecular structure using SHELXS-97 and SHELXL-97 software, respectively (G.M. Sheldrick, Crystal structure refinement with SHELXL, Acta Crystallographica Section C: Structural Chemistry 71(1) (2015) 3-8). The compound crystallizes in the monoclinic P2$_1$/c space group with a=12.88814(8) Å, b=7.73580(5) Å, c=15.92832(13) Å and β=102.1558(7)°. The number of independent molecules in the unit cell Z=4. The conformation of the molecule is shown in Figure S1.

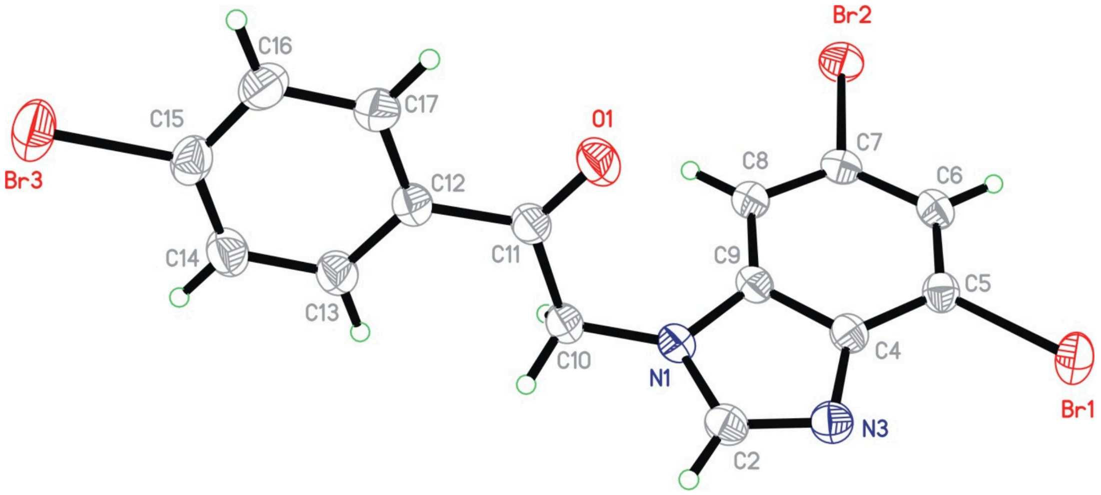

Figure S1. The ORTEP view of the molecule of 1-(4-bromophenyl)-2-(4,6-dibromo-1*H*-benzimidazol-1-yl)ethenone **5d**. The non hydrogen atoms are shown as 30% probability ellipsoids.

The crystal structure parameters have been deposited at the Cambridge Crystallographic Data Centre (deposition number CCDC 2171992). These data can be obtained free of charge via http://www.ccdc.cam.ac.uk/conts/retrieving.html.

Table S1. Crystal data and structure refinement for 1-(4-bromophenacyl)-4,6-dibromobenzimidazole (**5d**).

Identification code	CCDC 2171992
Empirical formula	$C_{15}H_9Br_3N_2O$
Formula weight	472.97
Temperature	293(2) K
Wavelength	1.54184 Å
Crystal system	Monoclinic

Space group	P 1 21/c 1
Unit cell dimensions	a = 12.88814(8) Å, α= 90°.
	b = 7.73580(5) Å, β= 102.1558(7)°.
	c = 15.92832(13) Å, γ = 90°.
Volume	1552.448(19) $Å^3$
Z	4
Density (calculated)	2.024 Mg/m^3
Absorption coefficient	9.638 mm^{-1}
F(000)	904
Crystal size	0.447 x 0.384 x 0.175 mm^3
Theta range for data collection	3.51 to 71.26°.
Index ranges	-15≤h≤15, -9≤k≤9, -18≤l≤19
Reflections collected	44722
Independent reflections	3015 [R(int) = 0.0529]
Completeness to theta = 71.26°	99.5 %
Absorption correction	Analytical
Max. and min. transmission	0.327 and 0.076
Refinement method	Full-matrix least-squares on F^2
Data / restraints / parameters	3015 / 0 / 191
Goodness-of-fit on F^2	1.072
Final R indices [I>2sigma(I)]	R1 = 0.0288, wR2 = 0.0751
R indices (all data)	R1 = 0.0330, wR2 = 0.0786
Extinction coefficient	0.00209(11)
Largest diff. peak and hole	0.407 and -0.448 e·$Å^{-3}$

7. Biological tests

MTT-based viability assay

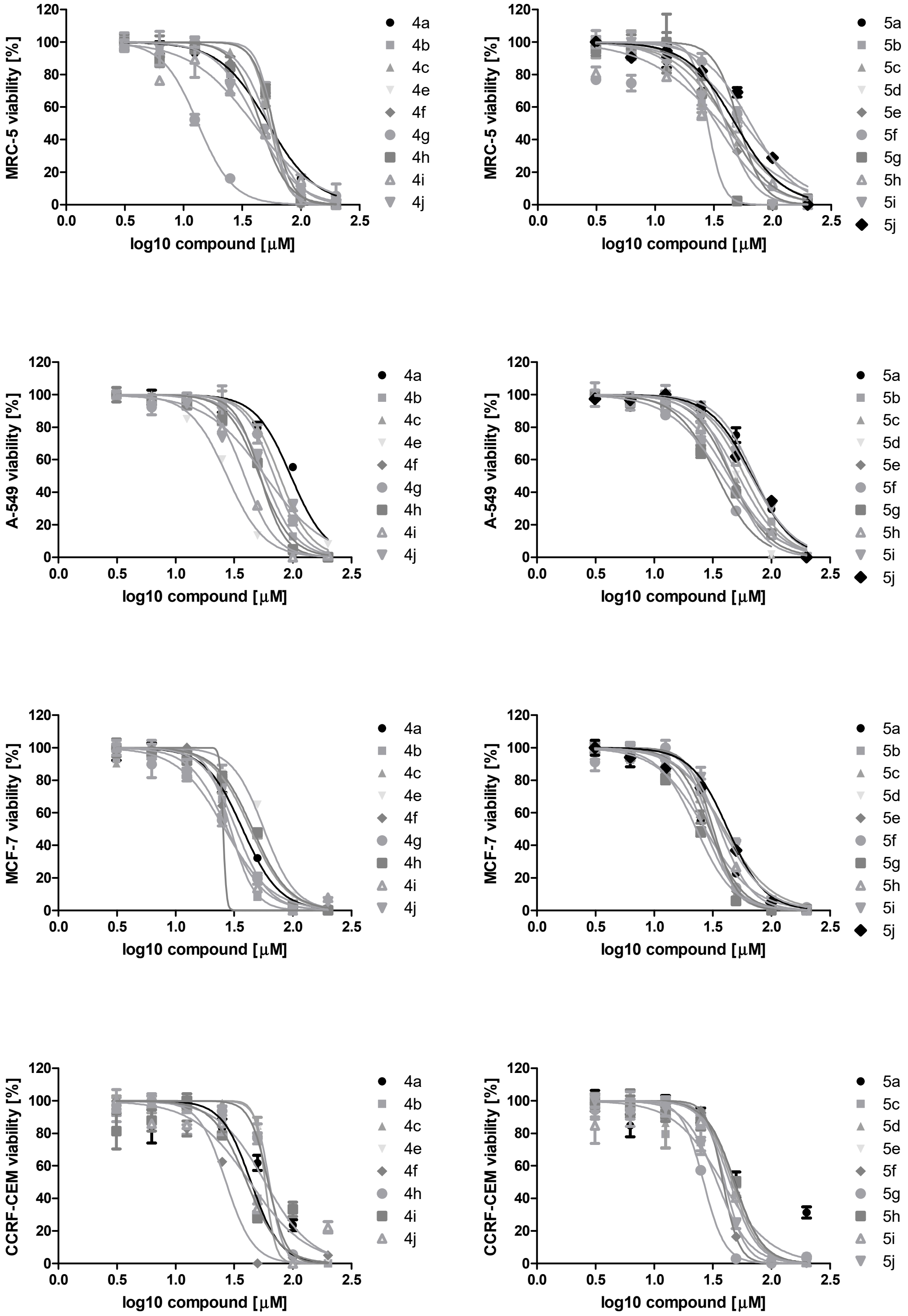

Figure S2. Representative sigmoidal dose-response curves for compounds **4a-4c**, **4e-4j** and **5a-5j** determined for MRC-5, A-549, MCF-7 and CCRF-CEM cell lines after 48 h of treatment. Plots were generated by GraphPad Prism after fitting the MTT data to sigmoidal dose response equation $Y=100/(1+10^{((LogIC_{50}-X)*HillSlope)})$.

Cytometry

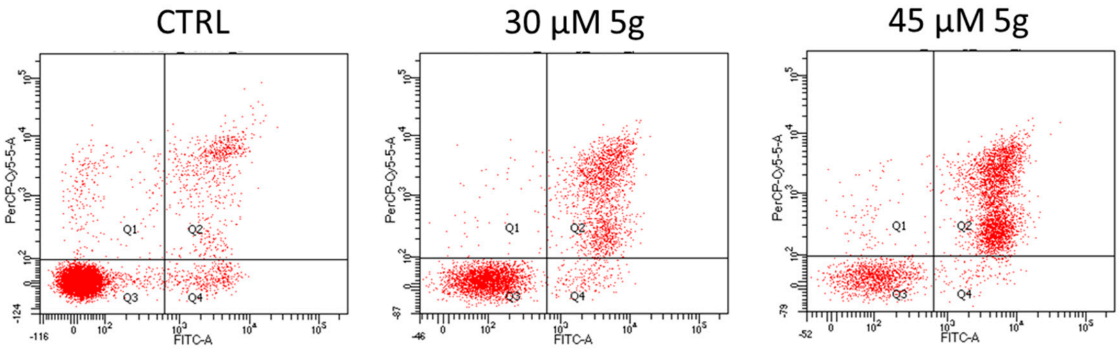

Figure S3. Representative cytograms for CCRF-CEM cells treated with compound **5g** for 48 h. Cells were stained with annexin V-FITC and PI (propidium iodide). Flow cytometry analyses were run on a FACSCanto II flow cytometer (BD Biosciences, San Diego, CA, USA) and analysed using BD FACSDiva software.
